# Supplementary material for: Patterns of multimorbidity and their effects on adverse outcomes in rheumatoid arthritis: a study of 5658 UK Biobank participants
Source: BMJ Open. 2020 Nov 23;10(11):e038829. doi: 10.1136/bmjopen-2020-038829 (PMC7684828; doi:10.1136/bmjopen-2020-038829)
Supplement: Supplementary data [file bmjopen-2020-038829supp001.pdf]

Tables

Supplementary table 1 – Proportion of long term conditions in participants with and without RA. P value determined using χ2 testing.

**Condition**	**Prevalence in RA participants (%)**	**Prevalence in non-RA participants (%)**	**p value**
**Hypertension**	**35·6**	**26·4**	**<0·01**
**Asthma**	**15·4**	**11·6**	**<0·01**
**Dyspepsia**	**11·3**	**7·7**	**<0·01**
**Thyroid disorder**	**9·5**	**5·8**	**<0·01**
**Cancer**	**8·7**	**7·7**	**<0·01**
**Coronary heart disease**	**8·2**	**4·5**	**<0·01**
**Diabetes**	**7·6**	**5·0**	**<0·01**
**Depression**	**7·0**	**5·6**	**<0·01**
**Osteoporosis**	**4·9**	**1·5**	**<0·01**
**Chronic obstructive pulmonary disease**	**4·4**	**1·6**	**<0·01**
Psoriasis/eczema	4·1	3·5	0·03
**IBS**	**3·3**	**2·3**	**<0·01**
Migraine	3·2	2·9	0·04
**Stroke/TIA**	**3·1**	**1·7**	**<0·01**
**Diverticular disease**	**2·2**	**1·1**	**<0·01**
Anxiety	1·7	1·8	0·47
**IBD**	**1·4**	**0·8**	**<0·01**
Prostate disease	1·3	1·6	0·06
**Pernicious anaemia**	**1·2**	**0·3**	**<0·01**
Glaucoma	1·2	1·1	0·26
Epilepsy	1·2	0·8	0·38
Endometriosis	0·9	0·8	0·39
Atrial fibrillation	0·9	0·7	0·14
**Peripheral vascular disease**	**0·9**	**0·3**	**<0·01**
**Chronic bronchitis**	**0·8**	**0·3**	**<0·01**
Chronic sinusitis	0·8	0·6	0·34
**Meniere's disease**	**0·7**	**0·3**	**<0·01**
Chronic kidney disease	0·5	0·3	0·01
**Chronic liver disease**	**0·4**	**0·2**	**<0·01**
Schizophrenia	0·4	0·4	0·68
Chronic fatigue syndrome	0·4	0·4	0·42
Alcohol problems	0·4	0·2	0·02
Viral hepatitis	0·3	0·3	0·91
Heart failure	0·3	0·2	0·18
Polycystic ovary syndrome	0·2	0·1	0·08
Multiple sclerosis	0·2	0·4	0·03
Parkinson's disease	0·1	0·2	0·71
Constipation	0·1	0·1	0·81
Dementia	0·1	0·02	0·17
Anorexia/bulimia	0·1	0·1	0·80

Psychoactive substance misuse	0·03	0·02	0·30

Supplementary table 2 –Medications, primary care read codes and hospitalisation codes used for RA self-report verification

Medications	Primary care read codes	Hospitalisation ICD-10 codes
Depomedrone	14G1	M05
Triamcinilone	F3712	M06
Methylprednisolone	F3964	
Prednisolone	G5yA.	
Prednisone	G5y8.	
Auranofin	H570.	
Azathioprine	N04..	
Hydroxychloroquine	N040.	
leflunomide	N0400	
Methotrexate	N0401	
Myocrisin	N0402	
Penicillamine	N0403	
Sulfasalazine	N0404	
Abatacept	N0405	
Adalimumab	N0406	
Certolizumab	N0407	
Etanercept	N0408	
Golimumab	N0409	
Infliximab	N040A	
Rituximab	N040B	
Tocilizumab	N040C	
	N040D	
	N040E	
	N040F	
	N040G	
	N040H	
	N040J	
	N040K	
	N040L	
	N040M	
	N040N	
	N040P	
	N040Q	
	N040R	
	N040S	
	N040T	
	N041.	
	N042.	
	N0421	
	N0422	
	N042z	
	N043.	
	N0430	

N0431
N0432
N0433
N043z
N047.
N04X.
N04y2
N0455
Nyu10
Nyu11
Nyu12
Nyu1G

Supplementary table 3 – Proportion of rheumatoid arthritis related hospitalisation, medication or primary care read code in participants who self-report rheumatoid arthritis.

Rheumatoid arthritis self-report	*Any rheumatoid arthritis hospitalisation, medication or primary care read code*		***Total***
	No	Yes	
No	141152<br>74.4 %	48634<br>25.6 %	189786<br>100 %
Yes	513<br>12.2 %	3683<br>87.8 %	4196<br>100 %
Total	141665<br>73 %	52317<br>27 %	193982<br>100 %

Supplementary table 4 – Demographic factors, lifestyle factors, number of long-term conditions and rheumatoid factor status in patients with and without RA. Unless indicated, p<0·01. Chi squared test used for categorical variables, Kruskal-Wallis test used for continuous variables. SD = standard deviation. RA defined here as RA self-report plus hospitalisation, medication or primary care read code related to rheumatoid arthritis.

	**Participants with RA (%) (N=3683)**	**Participants without RA (%) (N=498857)**
**Mean Age (years (SD)); missing values =0 (0%)**	59.2 (7.1)	56.5 (8.1)
**Age (years); missing values = 0 (0%)**		
37-49	413<br>11.2 %	117470<br>23.5 %
50-59	1161<br>31.5 %	165992<br>33.3 %
60-73	2109<br>57.3 %	215388<br>43.2 %
**Sex; missing values = 0 (0%)**		
Female	2672<br>72.5 %	270729<br>54.3 %
Male	1011<br>27.5 %	228121<br>45.7 %
**Townsend score; missing values = 623 (0·12%)**		
0-20	672<br>18.3 %	99991<br>20.1 %
20-40	666<br>18.1 %	99430<br>20 %
40-60	735<br>20 %	99663<br>20 %
60-80	760<br>20.7 %	99615<br>20 %
80-100	847<br>23 %	99531<br>20 %
**Smoking status; missing values = 2950 (0·59%)**		
Never	1679<br>46 %	271857<br>54.8 %
Current or Previous	1973<br>54 %	224074<br>45.2 %
**Frequency of alcohol intake; missing values = 1502 (0·30%)**		
Never or special occasions only	1218<br>33.1 %	97442<br>19.6 %
One to three times a month	453<br>12.3 %	55405<br>11.1 %
One to four times a week	1504<br>40.9 %	243237<br>48.9 %
Daily or almost daily	504<br>13.7 %	101268<br>20.4 %
**BMI (kg/m^2); missing values = 5820 (1·15%)**		
underweight <18.5	34<br>0.9 %	2592<br>0.5 %

normal weight 18.5-24.9	1084<br>30 %	156353<br>31.7 %
overweight 25-29.9	1425<br>39.5 %	212799<br>43.2 %
obese >=30s	1067<br>29.6 %	121359<br>24.6 %
**Physical activity; missing values = 7156 (1·42%)**		
none	595<br>16.6 %	32254<br>6.6 %
low	286<br>8 %	18652<br>3.8 %
medium	2596<br>72.4 %	390922<br>79.5 %
high	107<br>3 %	49965<br>10.2 %
**Number of long-term conditions; missing values = 1845 (0·36%)**		
0	922<br>25.2 %	174293<br>35.1 %
1	1103<br>30.1 %	163244<br>32.8 %
2-3	1255<br>34.3 %	135091<br>27.2 %
≥4	379<br>10.4 %	24401<br>4.9 %
**Rheumatoid Factor (IU/ml); missing values = 33,066 (6·6%)**		
<20	1801<br>52.4 %	449067<br>96.4 %
≥20	1639<br>47.6 %	16960<br>3.6 %

Supplementary Table 5 – Relationship between long term conditions and all-cause mortality in participants with and without RA using age-adjusted multivariate Cox's proportional hazards regression analysis. Unless otherwise shown, Cox's proportional hazards p<0·01. RA defined here as RA self-report plus hospitalisation, medication or primary care read code related to rheumatoid arthritis.

**Risk of all-cause mortality**			
**Comorbidity status (reference: *No RA and no other long-term conditions*)**		**Adjusted for sex, Townsend score, alcohol status, smoking status, BMI, physical activity and rheumatoid factor status HR (95% CI)**	**Number of deaths (%)**
**No other long-term conditions**	**RA**	1.50 (1.09 – 2.07)	44 (4.8%)
**1 other long-term condition**	**No RA**	1.39 (1.33 - 1.46)	5810 (3.6%)
	**RA**	1.42 (1.07 - 1.88)	66 (5.9%)
**2-3 other long-term conditions**	**No RA**	1.83 (1.75 - 1.91)	7966 (5.9%)
	**RA**	2.75 (2.29 - 3.30)	142 (11.3%)
**≥4 other long-term conditions**	**No RA**	2.70 (2.55 - 2.86)	2461 (10.8%)
	**RA**	2.98 (2.19 - 4.04)	54 (14.2%)

Supplementary Table 6 – Relationship between long term conditions and major adverse cardiovascular events in participants with and without RA using age-adjusted multivariate Cox's proportional hazards regression analysis. Unless otherwise shown, Cox's proportional hazards p<0·01. RA defined here as RA self-report plus hospitalisation, medication or primary care read code related to rheumatoid arthritis.

**Risk of MACE**			
**Comorbidity status (reference: *No RA and no other long-term conditions*)**		**Adjusted for sex, Townsend score, alcohol status, smoking status, BMI, physical activity and rheumatoid factor status HR (95% CI)**	**Number of MACE (%)**
**No other long-term conditions**	**RA**	1.63 (1.13 - 2.36)	32 (3.5%)
**1 other long-term condition**	**No RA**	1.24 (1.18 - 1.30)	4530 (2.8%)
	**RA**	1.95 (1.46 - 2.59)	60 (5.4%)
**2-3 other long-term conditions**	**No RA**	1.66 (1.58 - 1.74)	6244 (4.6%)
	**RA**	2.50 (2.00 - 3.12)	95 (7.6%)
**≥4 other long-term conditions**	**No RA**	2.38 (2.23 - 2.54)	2007 (8.2%)
	**RA**	3.30 (2.36 - 4.61)	46 (12.1%)

Supplementary Table 7 – Table 4 Risk of all-cause mortality for individual index conditions in patients with RA and no index condition, RA with index condition, RA with no index condition or RA and index condition. Age-adjusted Cox's proportional hazards models were adjusted for sex, Townsend score, smoking status, alcohol intake frequency, BMI, physical activity level and level of rheumatoid factor. Unless otherwise shown, Cox's proportional hazards p<0·01. Index conditions labelled * have interaction term p>0.01. RA defined here as RA self-report plus hospitalisation, medication or primary care read code related to rheumatoid arthritis.

	**Risk of all-cause mortality**			
	**No RA, no index condition HR, (95% CI), p**	**No RA, with index condition HR, (95% CI), p**	**RA, no index condition HR, (95% CI), p**	**RA and index condition HR, (95% CI), p**
**Index condition**				
Hypertension	1	1.24 1.20-1.28	1.27 1.07-1.52	1.69 1.41-2.02
Coronary heart disease	1	1.58 1.50-1.65	1.30 1.13-1.50	2.08 1.55-2.79
Diabetes	1	1.68 1.60-1.76	1.37 1.20-1.57	1.76 1.22-2.54
Asthma	1	1.10 1.05-1.15	1.32 1.14-1.52	1.48 1.10-2.00
Dyspepsia	1	1.02 0.97-1.07 p=0.42	1.31 1.15-1.50	1.46 1.04-2.06
Cancer	1	2.50 2.41-2.60	1.43 1.25-1.65	2.72 1.99-3.70
Depression	1	1.28 1.20-1.35	1.32 1.16-1.51	1.79 1.17-2.75
Thyroid disorder	1	1.05 0.99-1.12 p=0.12	1.36 1.19-1.55	1.14 0.76-1.72 p=0.53
COPD	1	2.12 1.98-2.26	1.32 1.15-1.50	2.53 1.77-3.63
Epilepsy	1	1.62 1.43-1.84	1.33 1.17-1.51	2.15 0.80-5.72 p=0.13
Migraine	1	0.85 0.76-0.94	1.33 1.17-1.51	1.02 0.38-2.71 p=0.97
Psoriasis /Eczema	1	1.06 0.94-1.14 p=0.15	1.30 1.14-1.49	2.08 1.23-3.50
Prostate disease	1	0.83 0.75-0.90	1.32 1.16-1.51	1.33 0.55-3.19 p=0.52
Osteoporosis	1	1.27 1.16-1.40	1.29 1.13-1.48	2.09 1.38-3.14
Atrial fibrillation	1	1.40 1.25-1.58	1.34 1.18-1.52	0.99 0.25-3.98 p=0.99
Anxiety	1	1.23 1.11-1.36	1.34 1.18-1.53	0.72 0.18-2.89 p=0.64
Inflammatory bowel disease	1	1.38 1.21-1.58	1.35 1.18-1.53	0.63 0.16-2.51 p=0.51
Heart failure	1	2.71 2.25-3.28	1.32 1.16-1.51	4.34 1.39-13.43

Supplementary Table 8 – Risk of MACE for individual index conditions in patients with RA and no index condition, RA with index condition, RA with no index condition or RA and index condition. Age-adjusted Cox's proportional hazards models were adjusted for sex, Townsend score, smoking status, alcohol intake frequency, BMI, physical activity level and level of rheumatoid factor. Unless otherwise shown, p<0·01. Index conditions labelled * have interaction term p>0.01. RA defined here as RA self-report plus hospitalisation, medication or primary care read code related to rheumatoid arthritis.

	Risk of MACE			
**Index condition**	**No RA, no index condition HR, (95% CI), p**	**No RA, with index condition HR, (95% CI), p**	**RA, no index condition HR, (95% CI), p**	**RA and index condition HR, (95% CI), p**
Hypertension	1	1.49 1.44-1.55	1.55 1.26-1.90	2.26 1.85-2.76
Coronary heart disease	1	1.89 1.80-1.98	1.60 1.37-1.88	2.31 1.65-3.22
Diabetes	1	1.66 1.58-1.75	1.62 1.39-1.90	1.66 1.58-1.75
Asthma	1	1.12 1.06-1.17	1.57 1.34-1.84	1.67 1.19-2.36
Dyspepsia	1	1.14 1.08-1.20	1.55 1.33-1.82	1.80 1.23-2.64
Cancer	1	1.11 1.05-1.17	1.59 1.37-1.85	1.42 0.87-2.33 p=0.16
Depression	1	1.25 1.17-1.34	1.53 1.31-1.78	2.38 1.52-3.74
Thyroid disorder	1	1.14 1.06-1.23	1.50 1.28-1.75	2.32 1.59-3.36
COPD	1	1.50 1.38-1.63	1.58 1.36-1.84	1.81 1.09-3.00
Epilepsy	1	1.50 1.31-1.74	1.56 1.35-1.81	1.74 0.44-6.97 p=0.43
Migraine	1	1.00 0.90-1.12 p=0.96	1.54 1.33-1.79	2.41 1.08-5.37
Psoriasis /Eczema	1	1.05 0.96-1.14 p=0.29	1.56 1.34-1.80	1.72 0.86-3.44 p=0.12
Prostate disease	1	0.91 0.83-1.00 p=0.05	1.53 1.32-1.78	2.53 1.20-5.31 p=0.01
Osteoporosis*	1	1.27 1.12-1.43	1.48 1.28-1.73	3.15 2.03-4.90
Atrial fibrillation	1	1.72 1.53-1.93	1.56 1.35-1.81	2.78 1.04-7.43 p=0.04
Anxiety	1	1.29 1.15-1.44	1.56 1.35-1.81	2.29 0.86-6.10 p=0.09
Inflammatory bowel disease	1	1.09 0.92-1.29 p=0.30	1.57 1.36-1.82	0.90 0.23-3.63 p=0.89
Heart failure	1	2.67 2.18-3.28	1.57 1.35-1.81	1.71 1.35-12.17 p=0.59
