# Supplementary material for: Patterns of multimorbidity and their effects on adverse outcomes in rheumatoid arthritis: a study of 5658 UK Biobank participants
Source: BMJ Open. 2020 Nov 23;10(11):e038829. doi: 10.1136/bmjopen-2020-038829 (PMC7684828; doi:10.1136/bmjopen-2020-038829)
Supplement: Supplementary data [file bmjopen-2020-038829supp002.pdf]

Supplementary figure 1 – Kaplan-Meier plot of proportion of all-cause mortality during the follow-up period (median 108 months) for participants with RA and no LTCS (black line), RA and 1 LTC (red line), RA and 2-3 LTCs (green line) and RA and ≥4 LTCs (blue line).

Supplementary figure 2 – Kaplan-Meier plot of proportion of MACE during the follow-up period (median 108 months) for participants with RA and no LTCS (black line), RA and 1 LTC (red line), RA and 2-3 LTCs (green line) and RA and ≥4 LTCs (blue line).

Supplementary figure 3 – Kaplan-Meier plot of proportion of all-cause mortality during the follow-up period (median 108 months) for participants no RA and no LTCS (black line), RA no RA and 1 LTC (red line), no RA and 2-3 LTCs (green line) and no RA and ≥4 LTCs (blue line).

Supplementary figure 4 – Kaplan-Meier plot of proportion of MACE during the follow-up period (median 108 months) for participants no RA and no LTCS (black line), RA no RA and 1 LTC (red line), no RA and 2-3 LTCs (green line) and no RA and ≥4 LTCs (blue line).
