# Supplementary figures and images for: Patterns of multimorbidity and their effects on adverse outcomes in rheumatoid arthritis: a study of 5658 UK Biobank participants
Source: BMJ Open. 2020 Nov 23;10(11):e038829. doi: 10.1136/bmjopen-2020-038829 (PMC7684828; doi:10.1136/bmjopen-2020-038829)

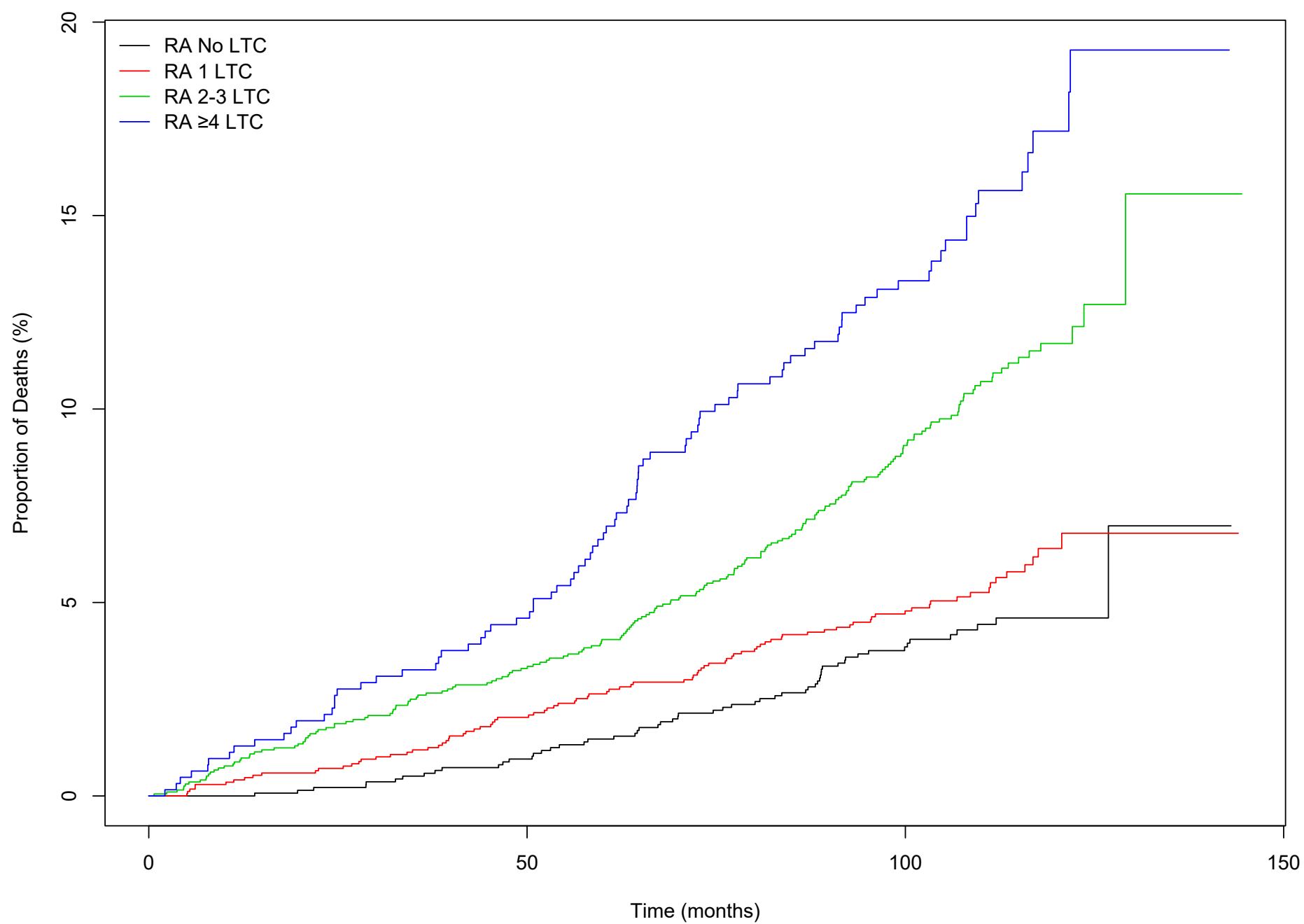

Supplement: Supplementary data [file bmjopen-2020-038829supp003.pdf]

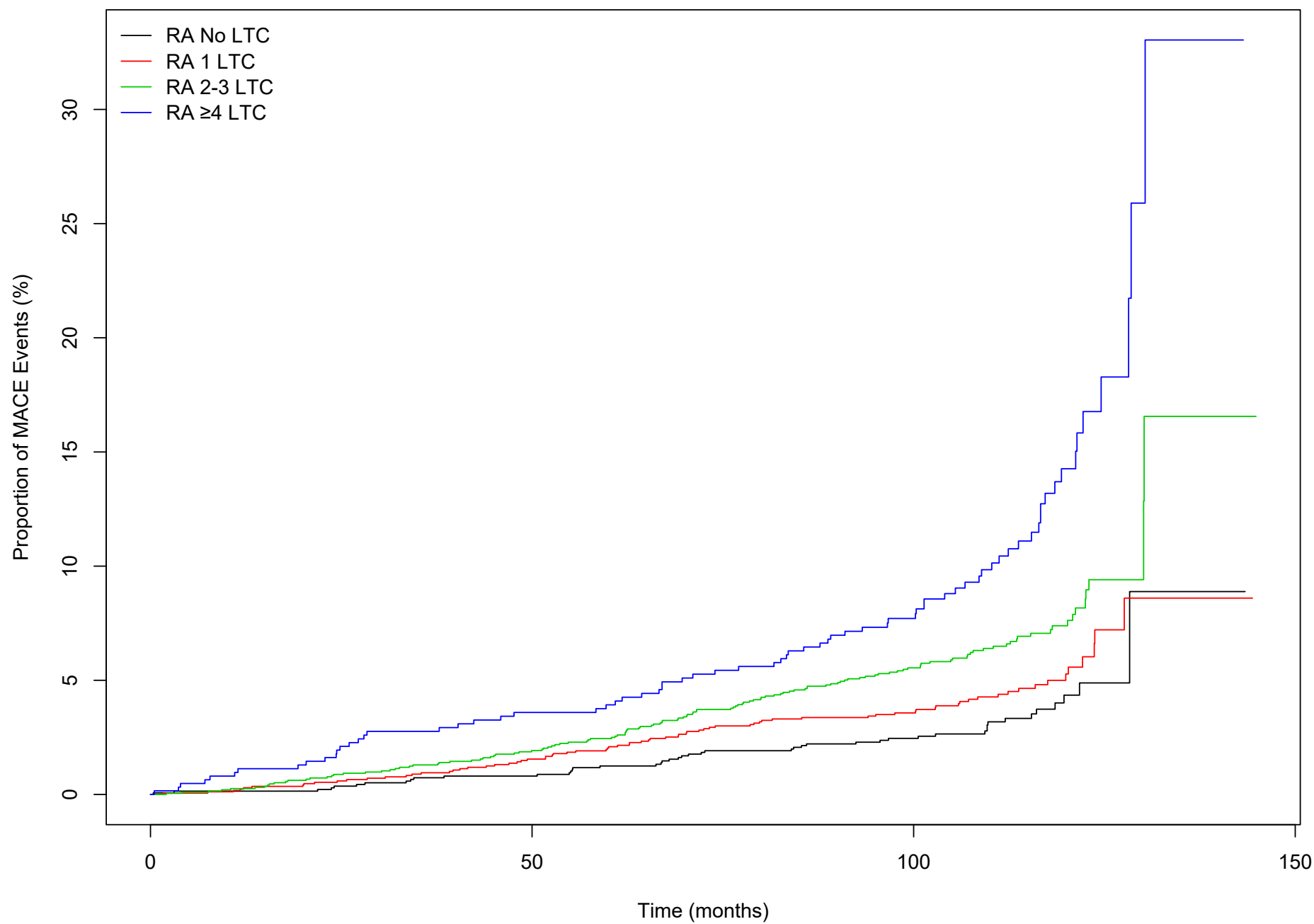

Supplement: Supplementary data [file bmjopen-2020-038829supp004.pdf]

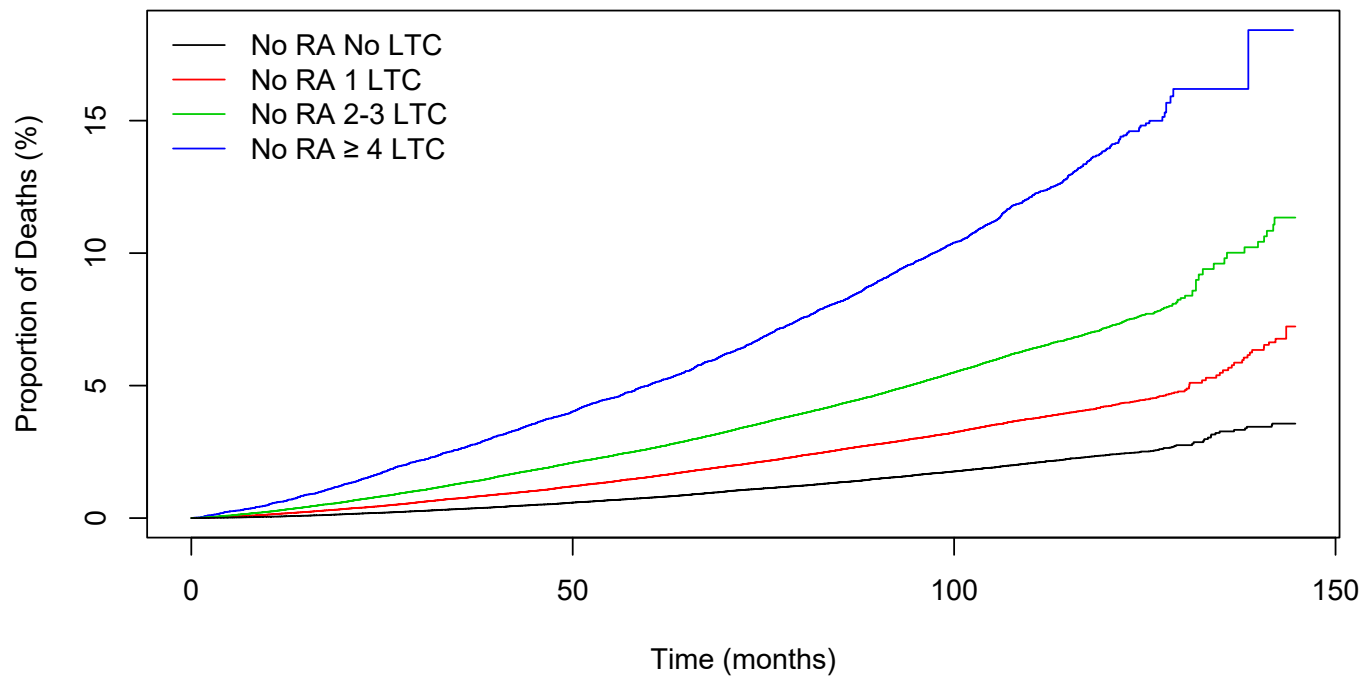

Supplement: Supplementary data [file bmjopen-2020-038829supp005.pdf]

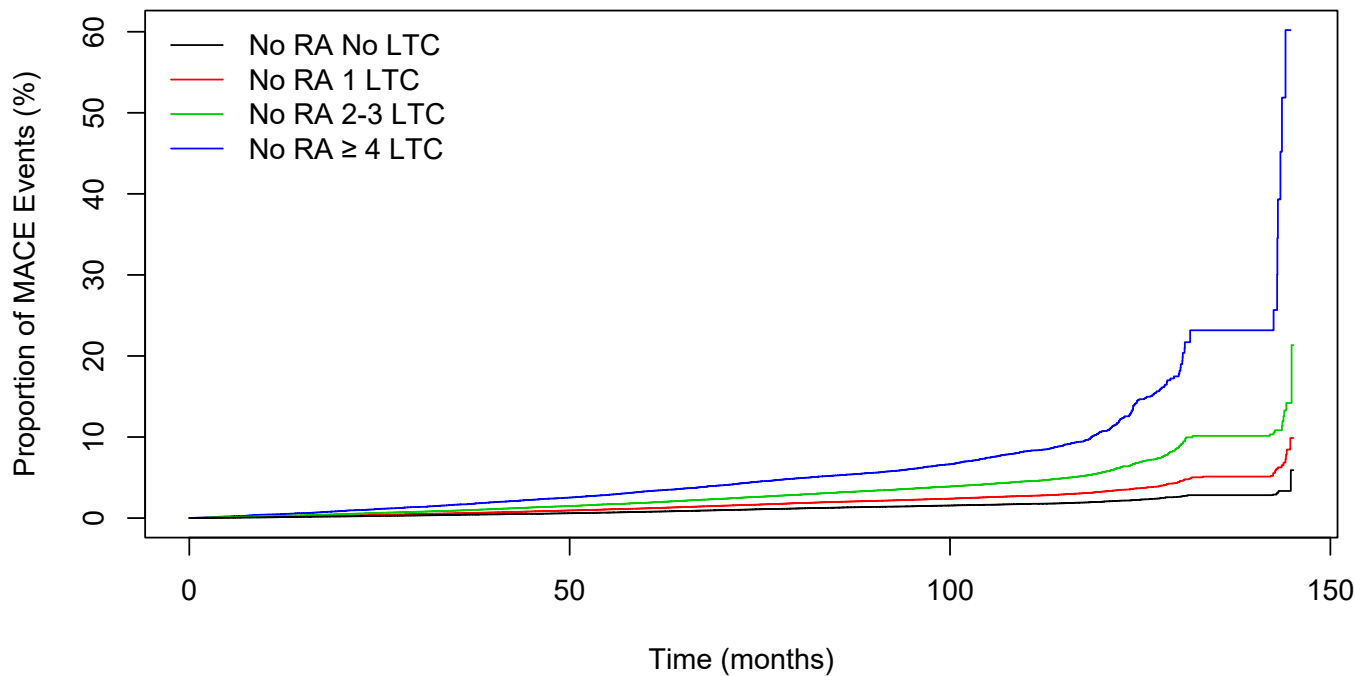

Supplement: Supplementary data [file bmjopen-2020-038829supp006.pdf]
