# Supplementary material for: Patterns of multimorbidity and their effects on adverse outcomes in rheumatoid arthritis: a study of 5658 UK Biobank participants
Source: BMJ Open. 2020 Nov 23;10(11):e038829. doi: 10.1136/bmjopen-2020-038829 (PMC7684828; doi:10.1136/bmjopen-2020-038829)
Supplement: Author's manuscript [file bmjopen-2020-038829.draft_revisions.pdf]

BMJ Open is committed to open peer review. As part of this commitment we make the peer review history of every article we publish publicly available.

When an article is published we post the peer reviewers' comments and the authors' responses online. We also post the versions of the paper that were used during peer review. These are the versions that the peer review comments apply to.

The versions of the paper that follow are the versions that were submitted during the peer review process. They are not the versions of record or the final published versions. They should not be cited or distributed as the published version of this manuscript.

BMJ Open is an open access journal and the full, final, typeset and author-corrected version of record of the manuscript is available on our site with no access controls, subscription charges or pay-per-view fees (http://bmjopen.bmj.com).

If you have any questions on BMJ Open's open peer review process please email info.bmjopen@bmj.com

BMJ Open

# Patterns of multimorbidity and their effects on adverse outcomes in rheumatoid arthritis: a study of 5658 UK Biobank participants

| | |
|---|---|
| Journal: | *BMJ Open* |
| Manuscript ID | bmjopen-2020-038829 |
| Article Type: | Original research |
| Date Submitted by the Author: | 27-Mar-2020 |
| Complete List of Authors: | McQueenie, Ross; University of Glasgow, GPPC, School of Medicine, Dentistry and Nursing, MVLS<br>Nicholl, Barbara; University of Glasgow, General Practice and Primary Care<br>Jani, Bhautesh; University of Glasgow, General Practice and Primary Care<br>Canning, Jordan; University of Glasgow, General Practice and Primary Care<br>MacDonald, Sara; University of Glasgow,<br>McCowan, Colin ; University of St. Andrews, School of Medicine<br>Neary, Joanne; University of Glasgow, General Practice and Primary Care<br>Browne, Susan; University of Glasgow, General Practice and Primary Care<br>Mair, Frances; University of Glasgow, General Practice and Primary Care<br>Siebert, Stefan; University of Glasgow, Institute of Infection, Immunity and Inflammation; NHS Greater Glasgow and Clyde, |
| Keywords: | RHEUMATOLOGY, CARDIOLOGY, EPIDEMIOLOGY |
| | |

SCHOLARONE™
Manuscripts

*I, the Submitting Author has the right to grant and does grant on behalf of all authors of the Work (as defined in the below author licence), an exclusive licence and/or a non-exclusive licence for contributions from authors who are: i) UK Crown employees; ii) where BMJ has agreed a CC-BY licence shall apply, and/or iii) in accordance with the terms applicable for US Federal Government officers or employees acting as part of their official duties; on a worldwide, perpetual, irrevocable, royalty-free basis to BMJ Publishing Group Ltd ("BMJ") its licensees and where the relevant Journal is co-owned by BMJ to the co-owners of the Journal, to publish the Work in this journal and any other BMJ products and to exploit all rights, as set out in our licence.*

*The Submitting Author accepts and understands that any supply made under these terms is made by BMJ to the Submitting Author unless you are acting as an employee on behalf of your employer or a postgraduate student of an affiliated institution which is paying any applicable article publishing charge ("APC") for Open Access articles. Where the Submitting Author wishes to make the Work available on an Open Access basis (and intends to pay the relevant APC), the terms of reuse of such Open Access shall be governed by a Creative Commons licence – details of these licences and which Creative Commons licence will apply to this Work are set out in our licence referred to above.*

*Other than as permitted in any relevant BMJ Author's Self Archiving Policies, I confirm this Work has not been accepted for publication elsewhere, is not being considered for publication elsewhere and does not duplicate material already published. I confirm all authors consent to publication of this Work and authorise the granting of this licence.*

For peer review only

*Patterns of multimorbidity and their effects on adverse outcomes in rheumatoid arthritis: a study of 5658 UK Biobank participants*

Ross McQueenie[1]

Barbara I Nicholl[1]

Bhautesh Dinesh Jani[1]

Jordan Canning[1]

Sara Macdonald[1]

Colin McCowan[3]

Joanne Neary[1]

Susan Browne[1]

Frances S Mair[1*]

Stefan Siebert[2*]

[1] Ross McQueenie BSc (Hons), PhD; Barbara Nicholl BSc, PhD; Bhautesh Dinesh Jani MRCGP, PhD; Jordan Canning BSc (Hons), MRes; Sara Maconald PhD; Joanne Neary BA (Hons), MRes, PhD; Susan Browne MA(Hons), Mphil, PhD; Frances Mair MD, DRCOG, FRCGP: General Practice and Primary Care, Institute of Health and Wellbeing, MVLS, University of Glasgow, UK

[2] Stefan Siebert MD, FRCP, PhD: Institute of Infection, Immunity and Inflammation, MVLS, University of Glasgow, UK

[3] Colin McCowan PhD: School of Medicine, University of St Andrews, UK

*Joint Senior author

Stefan Siebert (corresponding author)

Institute of Infection, Immunity and Inflammation University of Glasgow

G12 9LX

For peer review only

email: stefan.siebert@glasgow.ac.uk

No author received financial support for the work reported in this manuscript, and all others report no other financial interests that would be considered a conflict of interest.

*Abstract*

*Objective*

To investigate how type and number of long-term conditions (LTCs) impact on all-cause mortality and major adverse cardiovascular events (MACE) in people with RA.

*Design*

Population-based cross-sectional cohort study.

*Setting*

UK Biobank.

*Participants*

UK Biobank participants (N=502,533) aged between 37 and 73 years old.

*Primary outcome measures*

Primary outcome measures were risk of all-cause mortality and MACE.

*Methods*

We examined the relationship between LTC count and individual comorbid LTCs (N=42) on adverse clinical outcomes in participants with self-reported RA (N=5658). Risk of all-cause mortality and MACE were compared using Cox's proportional hazard models adjusted for

For peer review only

lifestyle factors (smoking, alcohol intake, physical activity), demographic factors (sex, age, socioeconomic status), and rheumatoid factor.

*Results*

75.7% of participants with RA had multimorbidity and these individuals were at increased risk of all-cause mortality and MACE. RA and ≥4LTCs showed a three-fold increased risk of all-cause mortality (hazard ratio (HR) 3.30, 95% confidence interval (CI) 2.61-4.16), and MACE (HR 3.45, 95% CI 2.66-4.49) compared to those without LTCs. Of the comorbid LTCs studied, osteoporosis was most strongly associated with adverse outcomes in participants with RA compared to those without RA or LTCs: two-fold increased risk of all-cause mortality (HR 2.20, 95% CI 1.55-3.12) and three-fold increased risk of MACE (HR 3.17, 95% CI 2.27-4.64). These findings remained in a subset (N=3683) with RA diagnosis validated from clinical records or medication reports.

*Conclusion*

Those with RA and other LTCs, particularly comorbid osteoporosis, are at increased risk of adverse outcomes. These results are clinically relevant for the monitoring and management of RA across the healthcare system, and future clinical guidelines for RA should acknowledge the importance of multimorbidity.

*Keywords*

Rheumatoid arthritis, mortality, multimorbidity, comorbidity, cardiovascular

For peer review only

*Strengths and limitations*

- This is the first study to examine both comorbidity and multimorbidity in RA and the associations with mortality and major adverse cardiovascular events (MACE).
- We used data from 5658 participants in UK Biobank with RA, including detailed information on participant demographics, lifestyle factors and rheumatoid factor status to examine multimorbidity and comorbidity using 42 non-RA LTCs.
- These results provide crucial new information which should be incorporated into clinical guidelines and used to influence management of peoples with RA.
- This study was limited by lack of information on RA disease severity which may play a role in both outcomes measured.

For peer review only

*Introduction*

Rheumatoid arthritis (RA) is a debilitating, chronic autoimmune disease characterised by inflammation of the synovial joints. RA is associated with physical and socio-economic issues, including increased pain levels, reduced physical functioning, and early mortality[1-3]. Globally, whilst disability adjusted life years for RA have improved since 1990, age-standardised prevalence and incidence rates are increasing [4].

Between 60% and 75% of those with RA are reported to have multimorbidity – two or more long-term conditions (LTCs) - with higher number of LTCs reported with increasing age and disease activity [5 6]. Common comorbidities include cardiovascular conditions[7] such as coronary artery disease [8] and cardiac failure [9], as well as mental health conditions such as depression [10]. Cardiovascular disease (CVD) accounts for the majority of the excess mortality observed in RA, with raised inflammatory markers and shared risk factors implicated [11]. However, the effects of comorbidities in RA have generally been studied in isolation and less is known regarding the risks posed by multimorbidity when RA co-occurs with more than one other long-term physical or mental health LTC.

Through analysis of UK Biobank data, this paper aims to explore the effect of multimorbidity and a wide range of comorbid LTCs on all-cause mortality and major adverse cardiovascular events (MACE) in people with RA. Our objectives were to:

1. Compare the effect of LTC count on all-cause mortality in those with and without self-reported RA.

2. Compare the effect of LTC count on MACE in those with and without self-reported RA.

3. Evaluate the effect of individual co-morbid LTCs on the risk of all-cause mortality and MACE in participants with self-reported RA.

For peer review only

*Patients and Methods*

*Study design and data collection*

This study utilised data from UK Biobank, a longitudinal population-based cohort of 502533 participants, aged 37-73 years in Great Britain.[12] Data was collected between 2006-10 from recruitment centres in Scotland, England and Wales, and subsequently linked to mortality and hospitalisation outcomes. A subset of primary care data was available for 230105 participants. This study was covered by the generic ethics approval for UK Biobank studies from the NHS National Research Ethics Service (16/NW/0274).

*Variables and outcome measures*

UK Biobank collected information on a wide range of demographic, health-based lifestyle and self-reported LTC questions through self-administered touch screen questionnaire and nurse-led interview. These include age, sex, socioeconomic status (measured using Townsend score, a UK area-based measure of deprivation) [13], smoking status, frequency of alcohol intake, body mass index (BMI), level of physical activity and number of LTCs.

Age was categorised into bands of 37-49, 50-59 and 60-73 years. Sex was a binary categorical variable. Smoking status was categorised into "never" or "current or previous". Frequency of alcohol intake was categorised into four groups, "Never or special occasions only", "One to three times a month", "One to four times a week" or "Daily or almost daily". BMI was categorised into four groups based on WHO BMI guidelines [14]: "underweight <18.5", "normal weight 18.5-24.9", "overweight 25-29.9" and "obese ≥30". Level of physical activity was defined as "none", "low", "medium", or "high" using Metabolic Equivalent Task (MET) scores data based on International Physical Activity Questionnaire (IPAQ) scoring protocol (available from https://sites.google.com/site/theipaq/scoring-protocol).

For peer review only

Rheumatoid factor was ascertained through participant blood samples and categorised into positive and negative status, with rheumatoid factor <20IU/ml considered negative, and values above this considered positive (by manufacturer specification, available at https://www.beckmancoulter.com/wsrportal/techdocs?docname=/cis/988646/%%/RF_988646-%25%25_English.pdf). Participants whose rheumatoid factor was labelled as "not reportable at assay (too low)" were considered to be rheumatoid factor negative. Similarly, those labelled "not reportable at assay (too high)" were considered rheumatoid factor positive.

The list of 42 LTCs considered was based on previous work in UK Biobank [15,16], the number of LTCs reported, apart from RA, were summed and then categorised as 0, 1, 2-3 and ≥4 LTCs. RA and all LTCs in UK Biobank are based on self-report using a questionnaire and nurse-led interview asking for existing diagnoses.

All-cause mortality was calculated using data linkage to national mortality registers. MACE were calculated using stroke and myocardial infarction (MI) hospitalisation event data from UK Biobank, and using ICD-10 mortality codes: "I00-I78", "G45", "G451-G454", "G456", "G458", "G459", and "G460-G468". The median follow-up time of both outcome measures was nine years.

A sensitivity analysis of self-report RA by participants was performed by examining four other indicators of RA: any primary care RA Read code, any secondary care RA hospitalisation code, self-reporting of any common RA drugs or any primary care prescription record of RA drugs (as shown in Supplementary Table 2). Both prospective and retrospective data were used: primary care Read codes were available for a maximum period of January 1991 and December 2017, and primary care prescriptions were between January 1991 and December 2016; the time period for each participant varied, depending on records held. Participants were considered to have confirmed RA if they had a positive record for one or more of these indicators. This

For peer review only

analysis was performed on a subset (74%) of participants who self-reported RA for whom primary care data in UK Biobank was available (N=4196/5658).

*Statistical methods*

In line with previous UK Biobank studies, $\chi 2$ tests were utilised for both categorical data and ordinal data. Kruskal–Wallis tests were used for continuous data [17]. Similarly, we used $\chi 2$ testing to examine differences in proportion of individual LTCs between those with and without RA. Age-adjusted Cox's proportional hazards tests were used to examine the relationship between LTC count / type of LTCs with all-cause mortality and MACE as outcome variables in those with and without RA. The model was further adjusted for demographic and lifestyle factors as described above. Among those with RA, cumulative hazards-based Kaplan-Meier plots were used to display proportion of events (all-cause mortality or MACE) in participants with 0, 1, 2-3 and ≥4 co-morbid LTCs. To measure the contribution of individual index LTCs towards all-cause mortality and MACE in those with and without RA, we created a categorical variable that assigned participants to one of four groups: those with neither RA nor the index condition (reference group), those with RA but not the index LTC (RA only), those with no RA with the index LTC (index LTC only), and those with both RA and the index LTC. This variable was used as an outcome measure in an age-adjusted Cox's proportional hazards model controlling for demographic factors, lifestyle factors and rheumatoid factor status. To calculate the interaction between RA and each index LTC, we used an ANOVA to measure p values between two Cox's proportional hazards models: the first containing RA and the index LTC, and the second containing RA, the index LTC and an interaction term between RA and the index LTC. Interaction terms were considered significant when $p<0.01$.

*Results*

For peer review only

5658 UK Biobank participants (1.1%) reported having RA. Lifestyle and demographic characteristics of participants with and without self-reported RA are shown in Table 1. Participants with RA were significantly more likely to be older, female, have lower socioeconomic status, be current or previous smokers, have a lower frequency of alcohol intake, have a BMI ≥30, have lower levels of physical activity, and have larger numbers of co-morbid LTCs. $\chi 2$ testing showed participants with self-reported RA were significantly more likely to have rheumatoid factor positive status: 35.6% had rheumatoid factor levels of over 20 IU/ml – compared with 3.6 % in those without RA.

*Prevalence of LTCs in people with RA*

Proportions of number of LTCs in participants with and without RA are shown in Table 1. Reporting multiple long-term conditions was more common in those with RA: 34.5% had 2-3 LTCs (27.1% in those without RA), and 11.1% had ≥ 4 LTCs (4.9% in those without RA). Overall, 75.7% of people with RA were noted to be multimorbid. The difference in comorbidity experienced by those with and without RA is shown in Supplementary Table 1. Those with RA reported proportionately higher numbers of physical and mental health-based LTCs, namely: cardiovascular LTCs including hypertension, coronary heart disease, and stroke or transient ischemic attack; pulmonary LTCs including asthma, COPD and chronic bronchitis; digestive system LTCs including dyspepsia, irritable bowel syndrome and inflammatory bowel disease; musculoskeletal conditions including osteoporosis; and mental-health based LTCs including depression.

*All-cause mortality and LTCs in people with RA*

We examined the outcomes associated with different LTC counts in participants with RA using a Kaplan Meier plot (Supplementary Figure 1). There was an increased proportion of all-cause mortality in participants with RA concurrent with increasing multimorbidity counts: 4.2%

For peer review only

(N=58) in those with no additional LTCs, 5.3% (N=91) in those with 1 additional LTC, 9.9% (N=194) in those with 2-3 additional LTCs and 14.4% (N=90) in those with ≥4 additional LTCs during the follow up period (median 9 years).

To quantify the effect of LTC count on all-cause mortality, we performed a Cox's proportional hazards test controlling for lifestyle factors, demographic factors and rheumatoid factor in participants with and without self-reported RA using a stepwise model adjustment (Table 2). Participants with RA and no additional LTCs had a significant increase in all-cause mortality when using an age-adjusted Cox's proportional hazards model fully adjusting for additional lifestyle and demographic factors (Hazard Ratio (HR) 1.59, 95% confidence intervals (CI) 1.21-2.08) compared to those without RA or any LTCs. Whilst controlling additionally for rheumatoid factor status appeared to show some attenuation of all-cause mortality risk, a statistically significant risk for this group remained (HR 1.39, 95% CI 1.05-1.84) when compared to those without RA or any LTCs. When examining additional co-morbid LTCs alongside RA, there appeared to be a dose-based response all-cause mortality risk, with a 44% increased risk of all-cause mortality in those with RA and one other LTC (HR 1.44, 95% CI 1.14-1.81), an approximately two-and-a-half-fold increased risk for RA with 2-3 other LTCs (HR 2.48, 95% CI 2.12-2.90) and an over three-fold increased risk associated for RA with ≥4 other LTCs (HR 3.30, 95% CI 2.61-4.16) compared to those without RA or any LTCs in the fully adjusted models, which included rheumatoid factor. A dose-based response was also observed in the non-RA population: those with 1 LTC had a 39% increased risk of death (HR 1.39, 95% CI 1.33-1.46), and those with ≥4 were at a two-and-a-half-fold increased risk (HR 2.69 95% CI 2.54-2.85) compared with participants without RA or any LTCs.

*MACE and LTCs in people with RA*

For peer review only

We next investigated the effect of LTC count on MACE in participants with RA using a Kaplan Meier plot (Supplementary Figure 2). For RA and no additional LTCs, 3.3% (N=46) of participants had a recorded MACE event, compared with 4.6% of participants with RA and one additional LTC (N=78), 6.7% those with RA and 2-3 additional LTCS (N=131), and almost four times as many proportionately in participants with RA and ≥4 LTCs (11.7%, N=73 events) over the follow-up period.

Table 3 shows the risk of MACE for participants with and without RA using age-adjusted multivariate Cox's proportional hazards regression models. There was a 63% increased hazard of MACE for participants with RA and no other LTCs compared with participants without RA or any LTCs (HR 1.63, 95% CI 1.21-2.21) in a fully adjusted model including demographic factors, lifestyle factors and rheumatoid factor status. This remained significant for people with RA with increasing LTCs count, with a 86% increased risk of MACE in participants with one other co-occurring LTC (HR 1.86, 95% CI 1.31-2.15), an over two-fold increase in those with 2-3 co-occurring LTCs (HR 2.09, 95% CI 1.73-2.54) and an almost three-and-a-half-fold increase in MACE for those with ≥4 LTCs (HR 3.39, 95% CI 2.61-4.40), compared to those without RA or any LTCs. This relationship was similar but to a lesser degree for participants without RA, with those with 1 LTC at 24% increased risk (HR 1.24, 95% CI 1.19-1.31), those with 2-3 LTCs at a 66% increased risk (HR 1.66, 95% CI 1.59-1.74) and those with ≥4 LTCs at over two times risk (HR 2.37 95% CI 2.23-2.53) of MACE compared with those without LTCs.

*Contribution of individual LTCs to all-cause mortality and MACE in people with RA*

Using an age-adjusted Cox's proportional hazards model, adjusting for demographic factors, lifestyle factors and rheumatoid factor status, we investigated the role individual LTCs play in

For peer review only

risk of all-cause mortality and MACE, using participants with no RA and no index condition as the reference group (Table 4 and 5).

The presence of cardiovascular-based LTCs appeared to be a risk factor in those with RA for both all-cause mortality and MACE. Compared to those with no RA and no hypertension, RA with hypertension showing an over one-and-a-half-fold increased risk of all-cause mortality (HR 1.59, 95% CI 1.37-1.86) and an approximately two-fold increased risk of MACE (HR 2.07, 95% CI 1.64-2.33).

Similarly, heart disease was associated with an over two-fold increase for both all-cause mortality (HR 2.07, 95% CI 1.63-2.63) and MACE (HR 2.28 95% CI 1.76-2.98) in those with RA compared to those with no RA and no heart disease. However, there was no evidence of interaction between RA and either cardiovascular condition. Whilst thyroid disorders showed no significant increased risk of all-cause mortality, they displayed an over two-fold increased risk of MACE (HR 2.10, 95% CI 1.50-2.93) in those with RA compared to those without RA or thyroid disease but again there was no significant interaction between RA and thyroid disease and MACE event.

The co-occurrence of osteoporosis in participants with RA appeared to strongly influence both mortality and MACE; more than doubling all-cause mortality (HR 2.20, 95% CI 1.55-3.12), and resulting in an over three times higher risk of MACE (HR 3.17, 95% CI 2.17-4.64) compared to those without RA or osteoporosis. This increased risk in those with both RA and osteoporosis was greater than in those with RA but no osteoporosis or those with osteoporosis but no RA. Interaction terms for RA and osteoporosis showed no significant interaction with all-cause mortality ($p=0.10$) but displayed a significant interaction with MACE ($p<0.01$), suggesting a multiplicative effect in the association with MACE.

*Sensitivity analysis of RA self-report*

For peer review only

To investigate sensitivity of self-report by participants with RA, was examined the proportion of people with any primary care RA Read code, any secondary care RA hospitalisation code, self-reporting of any common RA drugs and any primary care prescription record of RA drugs (see supplementary table 2) for participants who had self-reported RA and had available primary care data available in UK Biobank (N=4196). Medications used here were previously reported by Siebert et al.[17] Using this method, we were able to identify RA medications, hospitalisations or primary care Read code in 3683 (87.8%) participants (Supplementary Table 3). Analysis performed in this study was repeated in these participants and showed the same relationships as those reported above in N=5658 with self-report RA, with only small changes in HR observed (Supplementary Tables 4-8).

*Discussion*

Within UK Biobank, multiple LTCs was common in participants with RA, with approximately 75.7% reporting multimorbidity and 45% of participants reporting two or more additional LTCs alongside RA. In our fully adjusted modes, increasing LTC count was associated with increased mortality and MACE in people with RA. When examining individual LTCs, we observed hypertension, heart disease, osteoporosis and thyroid disorders to increase risk of adverse outcomes. Of these, osteoporosis was associated with one of the largest increases in both adverse outcomes measured: participants with both RA and osteoporosis were at over three times the risk of all-cause mortality and two times the risk of compared to those with neither LTC. The negative effect of having both RA and osteoporosis was particularly evident in MACE outcomes, for which there was a significant interaction between RA and osteoporosis, suggesting a multiplicative effect on MACE of having both these conditions together. The presence of hypertension or heart disease alongside RA increased the risk of mortality and MACE, in keeping with previous literature [18 19], but there was no evidence of statistical interaction.

For peer review only - http://bmjopen.bmj.com/site/about/guidelines.xhtml

To the best of our knowledge, this paper is the first to compare LTC count and type of comorbid LTCs and their association with all-cause mortality and MACE in men and women with RA after adjusting for a wide range of sociodemographic and lifestyle variables along with rheumatoid factor status. In our study, increasing LTC count resulted in adverse outcomes in participants with RA, with an increased rate of all-cause mortality and MACE.

We have shown that multimorbidity is common in participants with RA, with around 75% of participants with RA reporting one or more additional LTCs. This is in agreement with reported comorbidity rates of between 60% and 75% in those with RA [5 6], although these studies typically examined a smaller number of LTCs than in this study. We have shown participants with RA and 2-3 other LTCs were at over twice the risk of all-cause mortality, whilst those with ≥ 4 more were over three times the risk compared to participants with no LTCs. This data provides evidence for the first time the increased risk of all-cause mortality in men and women with RA and multimorbidity. While previous work has highlighted an increased risk of mortality in RA patients [20 21], or specific comorbidities alongside RA – for example in COPD [22] and depression [10] – these studies did not examine the effect of LTC count. One matched cohort study used a multimorbidity weighted index to study the effect of multimorbidity on mortality, but only examined effects in women [23]. Another examined LTCs using the Charlson comorbidity index [24] , however this measure uses only 19 LTCs and the study examined only all-cause mortality outcomes. Our study is the first study of its type to link multimorbidity in RA with MACE outcomes. Existing research has highlighted that RA increases the risk of cardiovascular events, and that individual LTCs such as diabetes and hypertension are risk factors [25], however, to date, no study has shown an association between multimorbidity and MACE outcomes in people with RA. Collectively, the results presented here report for the first time the magnitude of adverse outcomes associated with multimorbidity in those with RA.

For peer review only

326 In keeping with previous studies,[26] we have shown that osteoporosis prevalence is increased in those with RA compared to those without RA. The results presented in this paper, however, are the first to link osteoporosis in those with RA to increased risk of adverse outcomes and the first to show significant interaction between both conditions and MACE outcomes. The reasons for this association are not clear and cannot be extrapolated from the available data, which does not include factors such as disease severity or duration. One possibility may be that corticosteroids and RA disease activity play a role: corticosteroids are associated with increased prevalence of osteoporosis; people with RA with higher levels of disease activity are more likely to receive corticosteroids; both corticosteroid use and increased RA disease activity are reported to be associated with worse outcomes in mortality and MACE [27 28].

336 Our study therefore has several strong clinical implications. Current NICE guidelines for RA suggest annual checks for the development of hypertension, ischemic heart disease, osteoporosis and depression in RA [29], but do not highlight the increased risk of the co-occurrence of these LTCs with RA nor the risk posed by multimorbidity in general. In addition, we have shown a greatly increased risk of adverse outcomes in people with osteoporosis and RA that merits further investigation.

342 Our study has several key strengths: UK Biobank is a large population-based study with several thousand participants reporting RA; the study setting encompasses three countries within the UK (Scotland, England and Wales); it includes details of participant demographic and lifestyle factors as well as rheumatoid factor levels, which allowed us to adjust for variables, which have not been explored in previous studies.

347 Our study is limited by self-reporting of RA and LTCs by these participants; however, recent studies have shown that self-report is a reliable method for reporting RA [30] and in this study we additionally used four RA indicators (any primary care RA Read code, any secondary care

For peer review only

RA hospitalisation code, self-reporting of any common RA drugs and any primary care prescription record of RA drugs) to validate self-reported RA. We performed a sensitivity analysis using the subset of participants who had validated RA. Using this validation approach, we found a positive verification rate (participants self-reporting RA with further RA indicators) of 87.8% (N=3683). Re-analysis of the subset of participants with RA (Supplementary Tables 4-8) who had a validated RA report (N=3683) showed only small changes to Cox's proportional hazards models, and observed effects were in agreement with the population who self-reported RA. This provides confidence in our findings that we are examining a true RA population. Furthermore, we were unable to determine the severity or duration of RA in participants, or their previous medications.

Rheumatoid factor positive status in those self-reporting RA (35.6%) was lower than expected, however still a significantly higher proportion than in the UK Biobank population who did not report RA (3.6%). Analysis of rheumatoid factor in those who had RA primary care Read codes, prescriptions or hospitalisations (described above) showed an increased proportion of positive rheumatoid factor (47.6%), but this level remained below previously reported proportions in RA populations. However, our validation of self-report RA suggests that we can be confident that we have a high level of true RA included, regardless of rheumatoid factor levels.

Participants in UK Biobank are known to be less deprived than the wider UK population [31], suggesting that the level of multimorbidity reported here; and resulting associations are likely to be conservative in nature.

*Conclusions*

Multimorbidity is common in people with RA and is associated with increased risk of all-cause mortality and MACE. Certain comorbidities such as osteoporosis merit specific attention, in

For peer review only

view of their association with adverse outcomes; it will be important to test whether this association is replicated in other datasets and if so, to explore the underpinning mechanisms. As multimorbidity has been shown here to influence outcomes for those with RA, forthcoming work will examine which clusters of LTCs most strongly drive this increased risk of poor outcomes. Future clinical guidelines for RA should acknowledge the importance of multimorbidity when considering management planning and patient outcomes.

Word count: 3683 words

For peer review only

*Acknowledgements*

This research has been conducted using the UK Biobank Resource, approved project number 14151; we are like to thank the participants and those managing the data. We would like to acknowledge Versus Arthritis for funding this project.

*Ethical approval*

All participants gave informed consent for data provision and linkage. UK Biobank has full ethical approval from the NHS National Research Ethics Service (16/NW/0274).

*Competing interests*

None declared.

*Funding*

This study was funded by Versus Arthritis (grant number 21970)

*Data sharing statement*

The data used in this study are available via a direct application to UK Biobank.

*Author contributions*

This study was conceived by BN, FSM, SS, BJ and CM. The analysis was conducted by RM, BN and BJ. All authors (RM, BJ, BN, JC, SM, CM, JN, SB, FSM, SS) contributed to design, interpretation and discussion of all analysis. RM wrote this manuscript. All authors (RM, BJ, BN, JC, SM, CM, JN, SB, FSM, SS) edited, reviewed and commented on all versions of this manuscript. All authors read the manuscript draft and approved the final submission.

*Patient and Public Involvement*

The study was supported by a patient advisory group which provided input to the programme of research. This patient advisory group met on a regular basis for the duration of the study. Patients partnered with us and helped design research questions.

*References*

1. Andrews JS, Trupin L, Yelin EH, et al. Frailty and reduced physical function go hand in hand in adults with rheumatoid arthritis: a US observational cohort study. *Clinical rheumatology* 2017;36(5):1031-39. doi: 10.1007/s10067-017-3541-9 [published Online First: 2017/01/25]
2. Sokka T, Abelson B, Pincus T. Mortality in rheumatoid arthritis: 2008 update. *Clinical and experimental rheumatology* 2008;26(5 Suppl 51):S35-61. [published Online First: 2008/12/17]
3. Humphreys JH, Warner A, Chipping J, et al. Mortality trends in patients with early rheumatoid arthritis over 20 years: results from the Norfolk Arthritis Register. *Arthritis care & research* 2014;66(9):1296-301. doi: 10.1002/acr.22296 [published Online First: 2014/02/06]
4. Safiri S, Kolahi AA, Hoy D, et al. Global, regional and national burden of rheumatoid arthritis 1990–2017: a systematic analysis of the Global Burden of Disease study 2017. *Annals of the Rheumatic Diseases* 2019;78(11):1463. doi: 10.1136/annrheumdis-2019-215920
5. Espino-Lorenzo P, Manrique-Arija S, Urena I, et al. Baseline comorbidities in patients with rheumatoid arthritis who have been prescribed biological therapy: a case control study. *Reumatologia clinica* 2013;9(1):18-23. doi: 10.1016/j.reuma.2012.05.012 [published Online First: 2012/09/04]
6. Gron KL, Ornbjerg LM, Hetland ML, et al. The association of fatigue, comorbidity burden, disease activity, disability and gross domestic product in patients with rheumatoid arthritis. Results from 34 countries participating in the Quest-RA program. *Clinical and experimental rheumatology* 2014;32(6):869-77. [published Online First: 2014/10/21]
7. Nicola PJ, Crowson CS, Maradit-Kremers H, et al. Contribution of congestive heart failure and ischemic heart disease to excess mortality in rheumatoid arthritis. *Arthritis and rheumatism* 2006;54(1):60-7. doi: 10.1002/art.21560 [published Online First: 2005/12/31]
8. Solomon DH, Goodson NJ, Katz JN, et al. Patterns of cardiovascular risk in rheumatoid arthritis. *Ann Rheum Dis* 2006;65(12):1608-12. doi: 10.1136/ard.2005.050377 [published Online First: 2006/06/24]
9. Nicola PJ, Maradit-Kremers H, Roger VL, et al. The risk of congestive heart failure in rheumatoid arthritis: a population-based study over 46 years. *Arthritis and rheumatism* 2005;52(2):412-20. doi: 10.1002/art.20855 [published Online First: 2005/02/05]

For peer review only

10. Ang DC, Choi H, Kroenke K, et al. Comorbid depression is an independent risk factor for mortality in patients with rheumatoid arthritis. *The Journal of rheumatology* 2005;32(6):1013-9. [published Online First: 2005/06/09]

11. Solomon DH, Kremer J, Curtis JR, et al. Explaining the cardiovascular risk associated with rheumatoid arthritis: traditional risk factors versus markers of rheumatoid arthritis severity. *Ann Rheum Dis* 2010;69(11):1920-5. doi: 10.1136/ard.2009.122226 [published Online First: 2010/05/07]

12. Sudlow C, Gallacher J, Allen N, et al. UK biobank: an open access resource for identifying the causes of a wide range of complex diseases of middle and old age. *PLoS medicine* 2015;12(3):e1001779. doi: 10.1371/journal.pmed.1001779 [published Online First: 2015/04/01]

13. Townsend PJJosp. Deprivation. 1987;16(2):125-46.

14. Wolfe F. Pain extent and diagnosis: development and validation of the regional pain scale in 12,799 patients with rheumatic disease. *The Journal of rheumatology* 2003;30(2):369-78. [published Online First: 2003/02/04]

15. Jani BD, Nicholl BI, McQueenie R, et al. Multimorbidity and co-morbidity in atrial fibrillation and effects on survival: findings from UK Biobank cohort. *EP Europace* 2017;20(FI_3):f329-f36. doi: 10.1093/europace/eux322

16. Jani BD, Hanlon P, Nicholl BI, et al. Relationship between multimorbidity, demographic factors and mortality: findings from the UK Biobank cohort. *BMC Medicine* 2019;17(1):74. doi: 10.1186/s12916-019-1305-x

17. Siebert S, Lyall DM, Mackay DF, et al. Characteristics of rheumatoid arthritis and its association with major comorbid conditions: cross-sectional study of 502 649 UK Biobank participants. *RMD open* 2016;2(1):e000267.

18. Cooksey R, Brophy S, Kennedy J, et al. Cardiovascular risk factors predicting cardiac events are different in patients with rheumatoid arthritis, psoriatic arthritis, and psoriasis. *Seminars in arthritis and rheumatism* 2018;48(3):367-73. doi: 10.1016/j.semarthrit.2018.03.005 [published Online First: 2018/04/17]

19. Lauper K, Courvoisier DS, Chevallier P, et al. Incidence and Prevalence of Major Adverse Cardiovascular Events in Rheumatoid Arthritis, Psoriatic Arthritis, and Axial Spondyloarthritis. *Arthritis care & research* 2018;70(12):1756-63. doi: 10.1002/acr.23567 [published Online First: 2018/04/03]

20. van den Hoek J, Boshuizen HC, Roorda LD, et al. Mortality in patients with rheumatoid arthritis: a 15-year prospective cohort study. *Rheumatol Int* 2017;37(4):487-93. doi: 10.1007/s00296-016-3638-5 [published Online First: 2016/12/30]

21. Gabriel SE, Crowson CS, Kremers HM, et al. Survival in rheumatoid arthritis: a population-based analysis of trends over 40 years. *Arthritis and rheumatism* 2003;48(1):54-8. doi: 10.1002/art.10705 [published Online First: 2003/01/16]

22. Hyldgaard C, Løkke A, Pedersen AB, et al. Prognosis in rheumatoid arthritis patients with COPD: A nationwide, registry-based study. *European Respiratory Journal* 2017;50(suppl 61):OA1786. doi: 10.1183/1393003.congress-2017.OA1786

23. Yoshida K, Lin T-C, Wei M, et al. The roles of post-diagnosis accumulation of morbidities and lifestyle changes on excess total and cause-specific mortality risk in rheumatoid arthritis. *Arthritis care & research*;n/a(n/a) doi: 10.1002/acr.24120

24. Nikiphorou E, de Lusignan S, Mallen C, et al. Prognostic value of comorbidity indices and lung diseases in early rheumatoid arthritis: a UK population-based study. *Rheumatology* 2019 doi: 10.1093/rheumatology/kez409

25. Innala L, Moller B, Ljung L, et al. Cardiovascular events in early RA are a result of inflammatory burden and traditional risk factors: a five year prospective study. *Arthritis research & therapy* 2011;13(4):R131. doi: 10.1186/ar3442 [published Online First: 2011/08/17]

For peer review only

26. Haugeberg G, Uhlig T, Falch JA, et al. Bone mineral density and frequency of osteoporosis in female patients with rheumatoid arthritis: results from 394 patients in the Oslo County Rheumatoid Arthritis register. *Arthritis and rheumatism* 2000;43(3):522-30. doi: 10.1002/1529-0131(200003)43:3<522::Aid-anr7>3.0.Co;2-y [published Online First: 2000/03/23]

27. del Rincon I, Battafarano DF, Restrepo JF, et al. Glucocorticoid dose thresholds associated with all-cause and cardiovascular mortality in rheumatoid arthritis. *Arthritis & rheumatology (Hoboken, NJ)* 2014;66(2):264-72. doi: 10.1002/art.38210 [published Online First: 2014/02/08]

28. Listing J, Kekow J, Manger B, et al. Mortality in rheumatoid arthritis: the impact of disease activity, treatment with glucocorticoids, TNFalpha inhibitors and rituximab. *Ann Rheum Dis* 2015;74(2):415-21. doi: 10.1136/annrheumdis-2013-204021 [published Online First: 2013/12/03]

29. National Institute for Health and Care Excellence. Rheumatoid arthritis in adults: management: National Insttute for Health and Care Excellence, 2018.

30. Peeters GM, Alshurafa M, Schaap L, et al. Diagnostic accuracy of self-reported arthritis in the general adult population is acceptable. *J Clin Epidemiol* 2015;68(4):452-9. doi: 10.1016/j.jclinepi.2014.09.019 [published Online First: 2014/12/03]

31. Fry A, Littlejohns TJ, Sudlow C, et al. Comparison of Sociodemographic and Health-Related Characteristics of UK Biobank Participants With Those of the General Population. *American Journal of Epidemiology* 2017;186(9):1026-34. doi: 10.1093/aje/kwx246

*Figure legends*

Supplementary figure 1 – Kaplan-Meier plot of proportion of all-cause mortality during the follow-up period (median 108 months) for participants with RA and no LTCS (black line), RA and 1 LTC (red line), RA and 2-3 LTCs (green line) and RA and ≥4 LTCs (blue line).

Supplementary figure 1 – Kaplan-Meier plot of proportion of MACE during the follow-up period (median 108 months) for participants with RA and no LTCS (black line), RA and 1 LTC (red line), RA and 2-3 LTCs (green line) and RA and ≥4 LTCs (blue line).

For peer review only

For peer review only - http://bmjopen.bmj.com/site/about/guidelines.xhtml

522 *Tables*

523 Table 1 – Demographic factors, lifestyle factors, number of long-term conditions and rheumatoid factor status in
524 patients with and without rheumatoid arthritis. Unless indicated, p<0.01. $\chi 2$ test was used for categorical variables,
525 Kruskal-Wallis test was used for continuous variables. SD = standard deviation.

| | Participants with RA (%) (N=5658) | Participants without RA (%) (N=496882) |
|---|---|---|
| **Mean Age (years (SD)); missing values =0 (0%)** | 59.3 (7.1) | 56.5 (8.1) |
| **Age (years); missing values = 0 (0%)** | | |
| 37-49 | 675 (11.9%) | 117209 (23.6%) |
| 50-59 | 1800 (31.8%) | 165359 (33.3%) |
| 60-73 | 3183 (56.3%) | 214314 (43.1%) |
| **Sex; missing values = 0 (0%)** | | |
| Female | 3952 (69.8%) | 269452 (54.2%) |
| Male | 1706 (30.2%) | 227430 (45.8%) |
| **Townsend score; missing values = 623 (0.12%)** | | |
| 0-20 (least deprived) | 998 (17.7%) | 99665 (20.1%) |
| 20-40 | 980 (17.4%) | 99117 (20%) |
| 40-60 | 1087 (19.2%) | 99311 (20%) |
| 60-80 | 1154 (20.4%) | 99224 (20%) |
| 80-100 (most deprived) | 1429 (25.3%) | 98952 (19.9%) |
| **Smoking status; missing values = 2950 (0.59 %)** | | |
| Never | 2625 (46.8%) | 270916 (54.8%) |
| Current or Previous | 2983 (53.2%) | 223066 (45.2%) |
| **Frequency of alcohol intake; missing values = 1502 (0.30 %)** | | |
| Never or special occasions only | 1830 (32.4%) | 96832 (19.5%) |
| One to three times a month | 690 (12.2%) | 55170 (11.1%) |
| One to four times a week | 2315 | 242428 |

For peer review only

| | | |
|---|---|---|
| | (41%) | (48.9%) |
| Daily or almost daily | 811 (14.4%) | 100962 (20.4%) |
| **BMI (kg/m²); missing values = 5820 (1.15 %)** | | |
| underweight <18.5 | 50 (0.9%) | 2576 (0.5%) |
| normal weight 18.5-24.9 | 1543 (27.9%) | 155896 (31.7%) |
| overweight 25-29.9 | 2194 (39.6%) | 212032 (43.2%) |
| obese ≥30 | 1750 (31.6%) | 120679 (24.6%) |
| **Physical activity; missing values = 7156 (1.42 %)** | | |
| none | 814 (14.8%) | 32035 (6.5%) |
| low | 409 (7.4%) | 18531 (3.8%) |
| medium | 4111 (74.5%) | 389412 (79.5%) |
| high | 182 (3.3%) | 49890 (10.2%) |
| **Number of long-term conditions; missing values = 1845 (0.36 %)** | | |
| 0 | 1369 (24.3%) | 173846 (35.1%) |
| 1 | 1690 (30.0%) | 162657 (32.9%) |
| 2-3 | 1943 (34.5%) | 134403 (27.1%) |
| ≥4 | 623 (11.1%) | 24157 (4.9%) |
| **Rheumatoid Factor (IU/ml); missing values = 33,066 (6.6 %)** | | |
| <20 | 3396 (64.4%) | 447472 (96.4%) |
| >20 | 1879 (35.6%) | 16720 (3.6%) |

For peer review only

Table 2 Relationship between long term conditions and all-cause mortality in participants with and without self-reported RA using age-adjusted multivariate Cox's proportional hazards regression analysis. Unless otherwise shown, Cox's proportional hazards $p<0.01$.

**Risk of all-cause mortality**

| **Comorbidity status (reference: *No RA and no other long-term conditions*)** | | **Adjusted for sex and Townsend score HR (95% CI)** | **Adjusted for sex, Townsend score, alcohol status and smoking status HR (95% CI)** | **Adjusted for sex, Townsend score, alcohol status, smoking status, BMI and physical activity HR (95% CI)** | **Adjusted for sex, Townsend score, alcohol status, smoking status, BMI, physical activity and rheumatoid factor status HR (95% CI)** | **Number of deaths (%)** |
|---|---|---|---|---|---|---|
| **No other long-term conditions** | **RA** | 1.84 (1.42-2.38) | 1.72 (1.32-2.2) | 1.59 (1.21-2.08) | 1.39 (1.05-1.84) | 58 (4.2%) |
| **1 other long-term condition** | **No RA** | 1.45 (1.39-1.51) | 1.42 (1.36-1.48) | 1.40 (1.34-1.47) | 1.39 (1.33-1.46) | 5785 (3.6%) |
| | **RA** | 2.01 (1.64-2.48) | 1.88 (1.53-2.32) | 1.72 (1.38-2.14) | 1.44 (1.14-1.81) | 91 (5.4%) |
| **2-3 other long-term conditions** | **No RA** | 2.03 (1.95-2.11) | 1.92 (1.84-2.00) | 1.84 (1.77-1.92) | 1.83 (1.75-1.91) | 7914 (5.9%) |
| | **RA** | 3.32 (2.87-3.84) | 2.99 (2.59-3.46) | 2.79 (2.40-3.24) | 2.48 (2.12-2.90) | 194 (10.0%) |
| **≥4 other long-term conditions** | **No RA** | 3.39 (3.22-3.57) | 3.04 (2.88-3.20) | 2.71 (2.56-2.86) | 2.69 (2.54-2.85) | 2605 (10.8%) |
| | **RA** | 4.68 (3.80-5.78) | 3.95 (3.19-4.89) | 3.52 (2.81-4.40) | 3.30 (2.61-4.16) | 90 (14.4%) |

For peer review only

Table 3 Relationship between long term conditions and major adverse cardiovascular events in participants with and without self-reported RA using age-adjusted multivariate Cox's proportional hazards regression analysis. Unless otherwise shown, Cox's proportional hazards p<0.01.

**Risk of MACE**

| **Comorbidity status (reference: *No RA and no other long-term conditions*)** | | **Adjusted for sex and Townsend score HR (95% CI)** | **Adjusted for sex, Townsend score, alcohol status and smoking status HR (95% CI)** | **Adjusted for sex, Townsend score, alcohol status, smoking status, BMI, and physical activity HR (95% CI)** | **Adjusted for sex, Townsend score, alcohol status, smoking status, BMI, physical activity and rheumatoid factor status HR (95% CI)** | **Number of MACE (%)** |
|---|---|---|---|---|---|---|
| **No other long-term conditions** | **RA** | 1.79 (1.33-2.39) | 1.69 (1.26-2.27) | 1.64 (1.21-2.20) | 1.63 (1.21-2.21) | 46 (3.4%) |
| **1 other long-term condition** | **No RA** | 1.30 (1.24-1.36) | 1.28 (1.22-1.34) | 1.26 (1.20-1.320 | 1.24 (1.19-1.31) | 4512 (2.8%) |
| | **RA** | 2.08 (1.66-2.61) | 1.91 (1.52-2.41) | 1.87 (1.48-2.35) | 1.68 (1.31-2.15) | 78 (4.6%) |
| **2-3 other long-term conditions** | **No RA** | 1.86 (1.78-1.94) | 1.78 (1.70-1.86) | 1.67 (1.60-1.75) | 1.66 (1.59-1.74) | 6208 (4.6%) |
| | **RA** | 2.72 (2.28-3.24) | 2.49 (2.09-2.98) | 2.19 (1.82-2.64) | 2.09 (1.73-2.54) | 131 (6.7%) |
| **≥4 other long-term conditions** | **No RA** | 3.04 (2.87-3.22) | 2.76 (2.60-2.93) | 2.40 (2.26-2.56) | 2.37 (2.23-2.53) | 1980 (8.2%) |
| | **RA** | 4.79 (3.79-6.04) | 4.07 (3.21-5.16) | 3.52 (2.73-4.52) | 3.39 (2.61-4.40) | 73 (11.7%) |

For peer review only

Table 4 Risk of all-cause mortality for individual index conditions in patients with RA and no index condition, RA with index condition, RA with no index condition and RA and index condition. Age-adjusted Cox's proportional hazards models were adjusted for sex, Townsend score, smoking status, alcohol intake frequency, BMI, physical activity level and rheumatoid factor status. Unless otherwise shown, Cox's proportional hazards p<0.01. Index conditions labelled * have interaction term p>0.01

| | **Risk of all-cause mortality** | | | |
|---|---|---|---|---|
| | **No RA, no index condition HR, (95% CI), p** | **No RA, with index condition HR, (95% CI), p** | **RA, no index condition HR, (95% CI), p** | **RA and index condition HR, (95% CI), p** |
| **Index condition** | | | | |
| Hypertension | 1 | 1.24 1.21-1.28 | 1.29 1.11-1.48 | 1.59 1.37-1.86 |
| Coronary heart disease | 1 | 1.57 1.50-1.65 | 1.26 1.12-1.42 | 2.07 1.63-2.63 |
| Diabetes | 1 | 1.68 1.60-1.75 | 1.33 1.18-1.48 | 1.83 1.37-2.44 |
| Asthma | 1 | 1.10 1.05-1.15 | 1.27 1.13-1.42 | 1.56 1.22-2.00 |
| Dyspepsia | 1 | 1.01 0.97-1.06 p=0.47 | 1.27 1.14-1.43 | 1.45 1.10-1.90 |
| Cancer | 1 | 2.50 2.41-2.59 | 1.35 1.20-1.52 | 3.04 2.39-3.86 |
| Depression | 1 | 1.27 1.20-1.35 | 1.29 1.15-1.44 | 1.71 1.21-2.42 |
| Thyroid disorder | 1 | 1.05 0.98-1.12 p=0.11 | 1.32 1.18-1.47 | 1.14 0.80-1.62 p=0.46 |
| COPD | 1 | 2.11 1.98-2.49 | 1.26 1.13-1.42 | 2.68 2.00-3.58 |
| Epilepsy | 1 | 1.81 1.42-1.82 | 1.29 1.15-1.43 | 2.86 1.43-5.73 |
| Migraine | 1 | 0.85 0.76-0.94 | 1.29 1.16-1.44 | 1.09 0.55-2.19 p=0.79 |
| Psoriasis /Eczema | 1 | 1.05 0.98-1.14 p=0.16 | 1.27 1.14-1.42 | 1.88 1.20-2.95 |
| Prostate disease | 1 | 0.83 0.76-0.90 | 1.30 1.17-1.45 | 0.90 0.43-1.90 p=0.79 |
| Osteoporosis | 1 | 1.26 1.14-1.39 | 1.25 1.12-1.40 | 2.20 1.55-3.12 |
| Atrial fibrillation | 1 | 1.40 1.45-1.57 | 1.30 1.17-1.45 | 1.32 0.50-3.52 p=0.58 |
| Anxiety | 1 | 1.22 1.10-1.35 | 1.30 1.16-1.44 | 1.48 0.67-3.30 p=0.34 |
| Inflammatory bowel disease | 1 | 1.37 1.20-1.57 | 1.30 1.17-1.44 | 1.30 0.54-3.11 p=0.56 |
| Heart failure | 1 | 2.69 2.22-3.25 | 1.29 1.16-1.43 | 5.14 2.14-12.38 |

For peer review only

Table 5 Risk of MACE for individual index conditions in patients with RA and no index condition, RA with index condition, RA with no index condition and RA and index condition. Age-adjusted Cox's proportional hazards models were adjusted for sex, Townsend score, smoking status, alcohol intake frequency, BMI, physical activity level and rheumatoid factor status. Unless otherwise shown, p<0.01. Index conditions labelled * have interaction term p>0.01

| | Risk of MACE | | | |
|---|---|---|---|---|
| | **No RA, no index condition HR, (95% CI), p** | **No RA, with index condition HR, (95% CI), p** | **RA, no index condition HR, (95% CI), p** | **RA and index condition HR, (95% CI), p** |
| **Index condition** | | | | |
| Hypertension | 1 | 1.50 1.44-1.55 | 1.48 1.25-1.75 | 1.97 1.66-2.33 |
| Coronary heart disease | 1 | 1.89 1.80-1.98 | 1.43 1.45-1.63 | 2.28 1.76-2.98 |
| Diabetes | 1 | 1.67 1.58-1.75 | 1.49 1.31-1.69 | 1.69 1.19-2.39 |
| Asthma | 1 | 1.12 1.06-1.18 | 1.43 1.25-1.63 | 1.47 1.09-1.98 |
| Dyspepsia | 1 | 1.14 1.08-1.20 | 1.39 1.22- 1.58 | 1.85 1.30-2.34 |
| Cancer | 1 | 1.11 1.04-1.17 | 1.43 1.26-1.62 | 1.44 0.98-2.11 p=0.07 |
| Depression | 1 | 1.25 1.17-1.34 | 1.39 1.22-1.58 | 2.06 1.41-3.00 |
| Thyroid disorder | 1 | 1.14 1.03-1.23 | 1.37 1.20-1.55 | 2.10 1.50-2.93 |
| COPD | 1 | 1.49 1.37-1.62 | 1.40 1.24-1.59 | 1.97 1.33-2.92 |
| Epilepsy | 1 | 1.50 1.30-1.73 | 1.41 1.21-1.60 | 2.21 0.83-5.88 p=0.11 |
| Migraine | 1 | 0.99 0.89-1.12 p=0.97 | 1.40 1.23-1.58 | 2.08 1.12-3.87 |
| Psoriasis /Eczema | 1 | 1.05 0.96-1.14 p=0.25 | 1.42 1.26-1.61 | 1.23 0.64-2.37 p=0.53 |
| Prostate disease | 1 | 0.92 0.83-1.00 p=0.07 | 1.41 1.25-1.60 | 1.27 0.64-2.54 p=0.50 |
| Osteoporosis* | 1 | 1.34 1.18-1.53 | 1.25 1.10-1.41 | 3.17 2.17-4.64 |
| Atrial fibrillation | 1 | 1.41 1.25-1.60 | 1.72 1.53-1.93 | 2.67 1.99-5.95 |
| Anxiety | 1 | 1.28 1.14-1.43 | 1.40 1.24-1.59 | 2.73 1.30-5.72 |
| Inflammatory bowel disease | 1 | 1.09 0.92-1.29 p=0.32 | 1.42 1.26-1.60 | 1.11 0.36-3.44 p=0.85 |
| Heart failure | 1 | 2.64 2.15-3.24 | 1.41 1.25-1.59 | 3.45 1.11-10.70 p=0.03 |

For peer review only

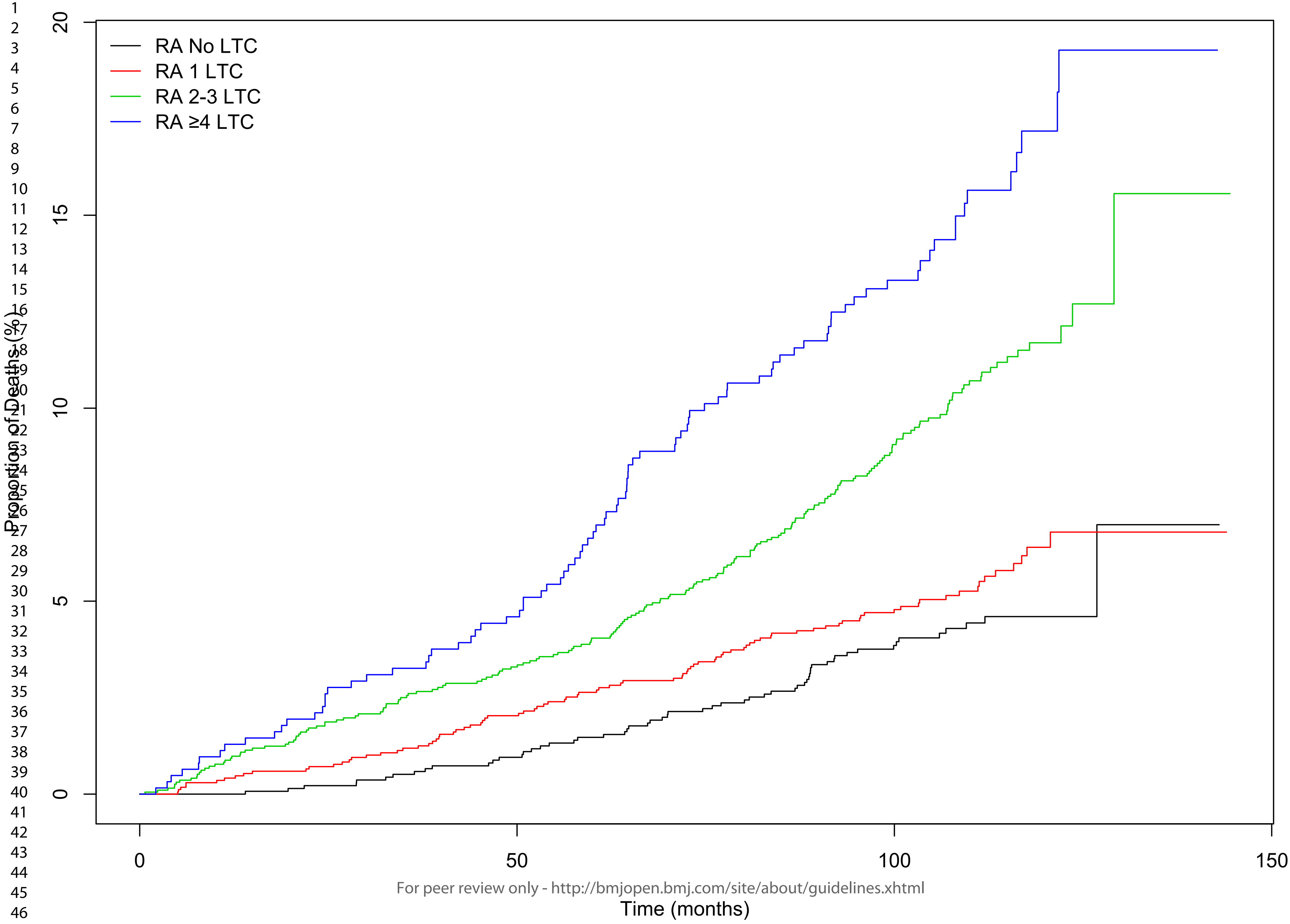
RA No LTC
RA 1 LTC
RA 2-3 LTC
RA ≥4 LTC
Proportion of Deaths (%)
0
5
10
15
20
0
50
100
150
Time (months)

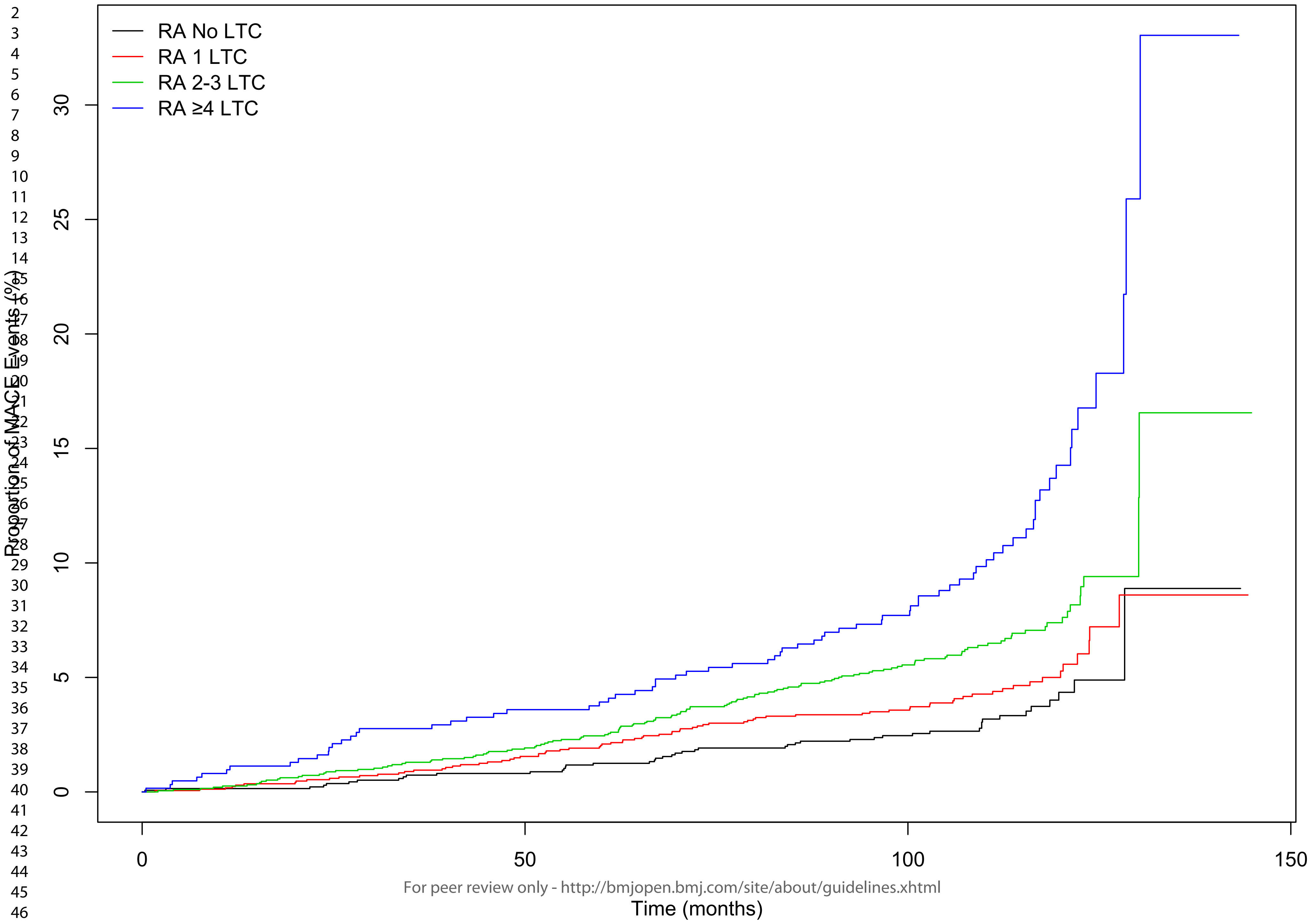
RA No LTC
RA 1 LTC
RA 2-3 LTC
RA ≥4 LTC
Proportion of MACE Events (%)
0
5
10
15
20
25
30
0
50
100
150
Time (months)

*Tables*

Supplementary table 1 – Proportion of long term conditions in participants with and without RA. P value determined using $\chi 2$ testing.

| Condition | Prevalence in RA participants (%) | Prevalence in non-RA participants (%) | p value |
|---|---|---|---|
| **Hypertension** | **35·6** | **26·4** | **<0·01** |
| **Asthma** | **15·4** | **11·6** | **<0·01** |
| **Dyspepsia** | **11·3** | **7·7** | **<0·01** |
| **Thyroid disorder** | **9·5** | **5·8** | **<0·01** |
| **Cancer** | **8·7** | **7·7** | **<0·01** |
| **Coronary heart disease** | **8·2** | **4·5** | **<0·01** |
| **Diabetes** | **7·6** | **5·0** | **<0·01** |
| **Depression** | **7·0** | **5·6** | **<0·01** |
| **Osteoporosis** | **4·9** | **1·5** | **<0·01** |
| **Chronic obstructive pulmonary disease** | **4·4** | **1·6** | **<0·01** |
| Psoriasis/eczema | 4·1 | 3·5 | 0·03 |
| **IBS** | **3·3** | **2·3** | **<0·01** |
| Migraine | 3·2 | 2·9 | 0·04 |
| **Stroke/TIA** | **3·1** | **1·7** | **<0·01** |
| **Diverticular disease** | **2·2** | **1·1** | **<0·01** |
| Anxiety | 1·7 | 1·8 | 0·47 |
| **IBD** | **1·4** | **0·8** | **<0·01** |
| Prostate disease | 1·3 | 1·6 | 0·06 |
| **Pernicious anaemia** | **1·2** | **0·3** | **<0·01** |
| Glaucoma | 1·2 | 1·1 | 0·26 |
| Epilepsy | 1·2 | 0·8 | 0·38 |
| Endometriosis | 0·9 | 0·8 | 0·39 |
| Atrial fibrillation | 0·9 | 0·7 | 0·14 |
| **Peripheral vascular disease** | **0·9** | **0·3** | **<0·01** |
| **Chronic bronchitis** | **0·8** | **0·3** | **<0·01** |
| Chronic sinusitis | 0·8 | 0·6 | 0·34 |
| **Meniere's disease** | **0·7** | **0·3** | **<0·01** |
| Chronic kidney disease | 0·5 | 0·3 | 0·01 |
| **Chronic liver disease** | **0·4** | **0·2** | **<0·01** |
| Schizophrenia | 0·4 | 0·4 | 0·68 |
| Chronic fatigue syndrome | 0·4 | 0·4 | 0·42 |
| Alcohol problems | 0·4 | 0·2 | 0·02 |
| Viral hepatitis | 0·3 | 0·3 | 0·91 |
| Heart failure | 0·3 | 0·2 | 0·18 |
| Polycystic ovary syndrome | 0·2 | 0·1 | 0·08 |
| Multiple sclerosis | 0·2 | 0·4 | 0·03 |
| Parkinson's disease | 0·1 | 0·2 | 0·71 |
| Constipation | 0·1 | 0·1 | 0·81 |
| Dementia | 0·1 | 0·02 | 0·17 |
| Anorexia/bulimia | 0·1 | 0·1 | 0·80 |

For peer review only

| | | | |
|---|---|---|---|
| Psychoactive substance misuse | 0·03 | 0·02 | 0·30 |

For peer review only

Supplementary table 2 –Medications, primary care read codes and hospitalisation codes used for RA self-report verification

| Medications | Primary care read codes | Hospitalisation ICD-10 codes |
|---|---|---|
| Depomedrone | 14G1 | M05 |
| Triamcinilone | F3712 | M06 |
| Methylprednisolone | F3964 | |
| Prednisolone | G5yA. | |
| Prednisone | G5y8. | |
| Auranofin | H570. | |
| Azathioprine | N04.. | |
| Hydroxychloroquine | N040. | |
| leflunomide | N0400 | |
| Methotrexate | N0401 | |
| Myocrisin | N0402 | |
| Penicillamine | N0403 | |
| Sulfasalazine | N0404 | |
| Abatacept | N0405 | |
| Adalimumab | N0406 | |
| Certolizumab | N0407 | |
| Etanercept | N0408 | |
| Golimumab | N0409 | |
| Infliximab | N040A | |
| Rituximab | N040B | |
| Tocilizumab | N040C | |
| | N040D | |
| | N040E | |
| | N040F | |
| | N040G | |
| | N040H | |
| | N040J | |
| | N040K | |
| | N040L | |
| | N040M | |
| | N040N | |
| | N040P | |
| | N040Q | |
| | N040R | |
| | N040S | |
| | N040T | |
| | N041. | |
| | N042. | |
| | N0421 | |
| | N0422 | |
| | N042z | |
| | N043. | |
| | N0430 | |

For peer review only

N0431
N0432
N0433
N043z
N047.
N04X.
N04y2
N0455
Nyu10
Nyu11
Nyu12
Nyu1G

For peer review only

Supplementary table 3 – Proportion of rheumatoid arthritis related hospitalisation, medication or primary care read code in participants who self-report rheumatoid arthritis.

| *Rheumatoid arthritis self-report* | *Any rheumatoid arthritis hospitalisation, medication or primary care read code* | | ***Total*** |
|---|---|---|---|
| | No | Yes | |
| No | 141152<br>74.4 % | 48634<br>25.6 % | 189786<br>100 % |
| Yes | 513<br>12.2 % | 3683<br>87.8 % | 4196<br>100 % |
| ***Total*** | 141665<br>73 % | 52317<br>27 % | 193982<br>100 % |

For peer review only

Supplementary table 4 – Demographic factors, lifestyle factors, number of long-term conditions and rheumatoid factor status in patients with and without RA. Unless indicated, p<0·01. Chi squared test used for categorical variables, Kruskal-Wallis test used for continuous variables. SD = standard deviation. RA defined here as RA self-report plus hospitalisation, medication or primary care read code related to rheumatoid arthritis.

| | **Participants with RA (%) (N=3683)** | **Participants without RA (%) (N=498857)** |
|---|---|---|
| **Mean Age (years (SD)); missing values =0 (0%)** | 59.2 (7.1) | 56.5 (8.1) |
| **Age (years); missing values = 0 (0%)** | | |
| 37-49 | 413<br>11.2 % | 117470<br>23.5 % |
| 50-59 | 1161<br>31.5 % | 165992<br>33.3 % |
| 60-73 | 2109<br>57.3 % | 215388<br>43.2 % |
| **Sex; missing values = 0 (0%)** | | |
| Female | 2672<br>72.5 % | 270729<br>54.3 % |
| Male | 1011<br>27.5 % | 228121<br>45.7 % |
| **Townsend score; missing values = 623 (0·12%)** | | |
| 0-20 | 672<br>18.3 % | 99991<br>20.1 % |
| 20-40 | 666<br>18.1 % | 99430<br>20 % |
| 40-60 | 735<br>20 % | 99663<br>20 % |
| 60-80 | 760<br>20.7 % | 99615<br>20 % |
| 80-100 | 847<br>23 % | 99531<br>20 % |
| **Smoking status; missing values = 2950 (0·59%)** | | |
| Never | 1679<br>46 % | 271857<br>54.8 % |
| Current or Previous | 1973<br>54 % | 224074<br>45.2 % |
| **Frequency of alcohol intake; missing values = 1502 (0·30%)** | | |
| Never or special occasions only | 1218<br>33.1 % | 97442<br>19.6 % |
| One to three times a month | 453<br>12.3 % | 55405<br>11.1 % |
| One to four times a week | 1504<br>40.9 % | 243237<br>48.9 % |
| Daily or almost daily | 504<br>13.7 % | 101268<br>20.4 % |
| **BMI ($kg/m^2$); missing values = 5820 (1·15%)** | | |
| underweight <18.5 | 34<br>0.9 % | 2592<br>0.5 % |

For peer review only

| | | |
|---|---|---|
| normal weight 18.5-24.9 | 1084<br>30 % | 156353<br>31.7 % |
| overweight 25-29.9 | 1425<br>39.5 % | 212799<br>43.2 % |
| obese >=30s | 1067<br>29.6 % | 121359<br>24.6 % |
| **Physical activity; missing values = 7156 (1·42%)** | | |
| none | 595<br>16.6 % | 32254<br>6.6 % |
| low | 286<br>8 % | 18652<br>3.8 % |
| medium | 2596<br>72.4 % | 390922<br>79.5 % |
| high | 107<br>3 % | 49965<br>10.2 % |
| **Number of long-term conditions; missing values = 1845 (0·36%)** | | |
| 0 | 922<br>25.2 % | 174293<br>35.1 % |
| 1 | 1103<br>30.1 % | 163244<br>32.8 % |
| 2-3 | 1255<br>34.3 % | 135091<br>27.2 % |
| ≥4 | 379<br>10.4 % | 24401<br>4.9 % |
| **Rheumatoid Factor (IU/ml); missing values = 33,066 (6·6%)** | | |
| <20 | 1801<br>52.4 % | 449067<br>96.4 % |
| ≥20 | 1639<br>47.6 % | 16960<br>3.6 % |

For peer review only

Supplementary Table 5 – Relationship between long term conditions and all-cause mortality in participants with and without RA using age-adjusted multivariate Cox's proportional hazards regression analysis. Unless otherwise shown, Cox's proportional hazards p<0·01. RA defined here as RA self-report plus hospitalisation, medication or primary care read code related to rheumatoid arthritis.

| Risk of all-cause mortality | | | |
|---|---|---|---|
| **Comorbidity status (reference: *No RA and no other long-term conditions*)** | | **Adjusted for sex, Townsend score, alcohol status, smoking status, BMI, physical activity and rheumatoid factor status HR (95% CI)** | **Number of deaths (%)** |
| **No other long-term conditions** | **RA** | 1.50 (1.09 – 2.07) | 44 (4.8%) |
| **1 other long-term condition** | **No RA** | 1.39 (1.33 - 1.46) | 5810 (3.6%) |
| | **RA** | 1.42 (1.07 - 1.88) | 66 (5.9%) |
| **2-3 other long-term conditions** | **No RA** | 1.83 (1.75 - 1.91) | 7966 (5.9%) |
| | **RA** | 2.75 (2.29 - 3.30) | 142 (11.3%) |
| **≥4 other long-term conditions** | **No RA** | 2.70 (2.55 - 2.86) | 2461 (10.8%) |
| | **RA** | 2.98 (2.19 - 4.04) | 54 (14.2%) |

For peer review only

Supplementary Table 6 – Relationship between long term conditions and major adverse cardiovascular events in participants with and without RA using age-adjusted multivariate Cox's proportional hazards regression analysis. Unless otherwise shown, Cox's proportional hazards p<0·01. RA defined here as RA self-report plus hospitalisation, medication or primary care read code related to rheumatoid arthritis.

| Risk of MACE | | | |
|---|---|---|---|
| **Comorbidity status (reference: *No RA and no other long-term conditions*)** | | **Adjusted for sex, Townsend score, alcohol status, smoking status, BMI, physical activity and rheumatoid factor status HR (95% CI)** | **Number of MACE (%)** |
| **No other long-term conditions** | **RA** | 1.63 (1.13 - 2.36) | 32 (3.5%) |
| **1 other long-term condition** | **No RA** | 1.24 (1.18 - 1.30) | 4530 (2.8%) |
| | **RA** | 1.95 (1.46 - 2.59) | 60 (5.4%) |
| **2-3 other long-term conditions** | **No RA** | 1.66 (1.58 - 1.74) | 6244 (4.6%) |
| | **RA** | 2.50 (2.00 - 3.12) | 95 (7.6%) |
| **≥4 other long-term conditions** | **No RA** | 2.38 (2.23 - 2.54) | 2007 (8.2%) |
| | **RA** | 3.30 (2.36 - 4.61) | 46 (12.1%) |

For peer review only

Supplementary Table 7 – Table 4 Risk of all-cause mortality for individual index conditions in patients with RA and no index condition, RA with index condition, RA with no index condition or RA and index condition. Age-adjusted Cox's proportional hazards models were adjusted for sex, Townsend score, smoking status, alcohol intake frequency, BMI, physical activity level and level of rheumatoid factor. Unless otherwise shown, Cox's proportional hazards $p<0{\cdot}01$. Index conditions labelled * have interaction term $p>0.01$. RA defined here as RA self-report plus hospitalisation, medication or primary care read code related to rheumatoid arthritis.

| | **Risk of all-cause mortality** | | | |
|---|---|---|---|---|
| | **No RA, no index condition HR, (95% CI), p** | **No RA, with index condition HR, (95% CI), p** | **RA, no index condition HR, (95% CI), p** | **RA and index condition HR, (95% CI), p** |
| **Index condition** | | | | |
| Hypertension | 1 | 1.24 1.20-1.28 | 1.27 1.07-1.52 | 1.69 1.41-2.02 |
| Coronary heart disease | 1 | 1.58 1.50-1.65 | 1.30 1.13-1.50 | 2.08 1.55-2.79 |
| Diabetes | 1 | 1.68 1.60-1.76 | 1.37 1.20-1.57 | 1.76 1.22-2.54 |
| Asthma | 1 | 1.10 1.05-1.15 | 1.32 1.14-1.52 | 1.48 1.10-2.00 |
| Dyspepsia | 1 | 1.02 0.97-1.07 p=0.42 | 1.31 1.15-1.50 | 1.46 1.04-2.06 |
| Cancer | 1 | 2.50 2.41-2.60 | 1.43 1.25-1.65 | 2.72 1.99-3.70 |
| Depression | 1 | 1.28 1.20-1.35 | 1.32 1.16-1.51 | 1.79 1.17-2.75 |
| Thyroid disorder | 1 | 1.05 0.99-1.12 p=0.12 | 1.36 1.19-1.55 | 1.14 0.76-1.72 p=0.53 |
| COPD | 1 | 2.12 1.98-2.26 | 1.32 1.15-1.50 | 2.53 1.77-3.63 |
| Epilepsy | 1 | 1.62 1.43-1.84 | 1.33 1.17-1.51 | 2.15 0.80-5.72 p=0.13 |
| Migraine | 1 | 0.85 0.76-0.94 | 1.33 1.17-1.51 | 1.02 0.38-2.71 p=0.97 |
| Psoriasis /Eczema | 1 | 1.06 0.94-1.14 p=0.15 | 1.30 1.14-1.49 | 2.08 1.23-3.50 |
| Prostate disease | 1 | 0.83 0.75-0.90 | 1.32 1.16-1.51 | 1.33 0.55-3.19 p=0.52 |
| Osteoporosis | 1 | 1.27 1.16-1.40 | 1.29 1.13-1.48 | 2.09 1.38-3.14 |
| Atrial fibrillation | 1 | 1.40 1.25-1.58 | 1.34 1.18-1.52 | 0.99 0.25-3.98 p=0.99 |
| Anxiety | 1 | 1.23 1.11-1.36 | 1.34 1.18-1.53 | 0.72 0.18-2.89 p=0.64 |
| Inflammatory bowel disease | 1 | 1.38 1.21-1.58 | 1.35 1.18-1.53 | 0.63 0.16-2.51 p=0.51 |
| Heart failure | 1 | 2.71 2.25-3.28 | 1.32 1.16-1.51 | 4.34 1.39-13.43 |

For peer review only

Supplementary Table 8 – Risk of MACE for individual index conditions in patients with RA and no index condition, RA with index condition, RA with no index condition or RA and index condition. Age-adjusted Cox's proportional hazards models were adjusted for sex, Townsend score, smoking status, alcohol intake frequency, BMI, physical activity level and level of rheumatoid factor. Unless otherwise shown, p<0·01. Index conditions labelled * have interaction term p>0.01. RA defined here as RA self-report plus hospitalisation, medication or primary care read code related to rheumatoid arthritis.

| | Risk of MACE | | | |
|---|---|---|---|---|
| **Index condition** | **No RA, no index condition HR, (95% CI), p** | **No RA, with index condition HR, (95% CI), p** | **RA, no index condition HR, (95% CI), p** | **RA and index condition HR, (95% CI), p** |
| Hypertension | 1 | 1.49 1.44-1.55 | 1.55 1.26-1.90 | 2.26 1.85-2.76 |
| Coronary heart disease | 1 | 1.89 1.80-1.98 | 1.60 1.37-1.88 | 2.31 1.65-3.22 |
| Diabetes | 1 | 1.66 1.58-1.75 | 1.62 1.39-1.90 | 1.66 1.58-1.75 |
| Asthma | 1 | 1.12 1.06-1.17 | 1.57 1.34-1.84 | 1.67 1.19-2.36 |
| Dyspepsia | 1 | 1.14 1.08-1.20 | 1.55 1.33-1.82 | 1.80 1.23-2.64 |
| Cancer | 1 | 1.11 1.05-1.17 | 1.59 1.37-1.85 | 1.42 0.87-2.33 p=0.16 |
| Depression | 1 | 1.25 1.17-1.34 | 1.53 1.31-1.78 | 2.38 1.52-3.74 |
| Thyroid disorder | 1 | 1.14 1.06-1.23 | 1.50 1.28-1.75 | 2.32 1.59-3.36 |
| COPD | 1 | 1.50 1.38-1.63 | 1.58 1.36-1.84 | 1.81 1.09-3.00 |
| Epilepsy | 1 | 1.50 1.31-1.74 | 1.56 1.35-1.81 | 1.74 0.44-6.97 p=0.43 |
| Migraine | 1 | 1.00 0.90-1.12 p=0.96 | 1.54 1.33-1.79 | 2.41 1.08-5.37 |
| Psoriasis /Eczema | 1 | 1.05 0.96-1.14 p=0.29 | 1.56 1.34-1.80 | 1.72 0.86-3.44 p=0.12 |
| Prostate disease | 1 | 0.91 0.83-1.00 p=0.05 | 1.53 1.32-1.78 | 2.53 1.20-5.31 p=0.01 |
| Osteoporosis* | 1 | 1.27 1.12-1.43 | 1.48 1.28-1.73 | 3.15 2.03-4.90 |
| Atrial fibrillation | 1 | 1.72 1.53-1.93 | 1.56 1.35-1.81 | 2.78 1.04-7.43 p=0.04 |
| Anxiety | 1 | 1.29 1.15-1.44 | 1.56 1.35-1.81 | 2.29 0.86-6.10 p=0.09 |
| Inflammatory bowel disease | 1 | 1.09 0.92-1.29 p=0.30 | 1.57 1.36-1.82 | 0.90 0.23-3.63 p=0.89 |
| Heart failure | 1 | 2.67 2.18-3.28 | 1.57 1.35-1.81 | 1.71 1.35-12.17 p=0.59 |

For peer review only

# Reporting checklist for cross sectional study.

Based on the STROBE cross sectional guidelines.

## Instructions to authors

Complete this checklist by entering the page numbers from your manuscript where readers will find each of the items listed below.

Your article may not currently address all the items on the checklist. Please modify your text to include the missing information. If you are certain that an item does not apply, please write "n/a" and provide a short explanation.

Upload your completed checklist as an extra file when you submit to a journal.

In your methods section, say that you used the STROBE cross sectionalreporting guidelines, and cite them as:

von Elm E, Altman DG, Egger M, Pocock SJ, Gotzsche PC, Vandenbroucke JP. The Strengthening the Reporting of Observational Studies in Epidemiology (STROBE) Statement: guidelines for reporting observational studies.

| | | Reporting Item | Page Number |
|---|---|---|---|
| **Title and abstract** | | | |
| Title | #1a | Indicate the study's design with a commonly used term in the title or the abstract | 1 |
| Abstract | #1b | Provide in the abstract an informative and balanced summary of what was done and what was found | 2 |

For peer review only

## Introduction

| | | | |
|---|---|---|---|
| Background / rationale | #2 | Explain the scientific background and rationale for the investigation being reported | 5 |
| Objectives | #3 | State specific objectives, including any prespecified hypotheses | 5 |

## Methods

| | | | |
|---|---|---|---|
| Study design | #4 | Present key elements of study design early in the paper | 6 |
| Setting | #5 | Describe the setting, locations, and relevant dates, including periods of recruitment, exposure, follow-up, and data collection | 6 |
| Eligibility criteria | #6a | Give the eligibility criteria, and the sources and methods of selection of participants. | n/a (data collected by UK Biobank) |
| | #7 | Clearly define all outcomes, exposures, predictors, potential confounders, and effect modifiers. Give diagnostic criteria, if applicable | 6 |
| Data sources / measurement | #8 | For each variable of interest give sources of data and details of methods of assessment (measurement). Describe comparability of assessment methods if there is more than one group. Give information separately for for exposed and unexposed groups if applicable. | 6-7 |

For peer review only

| | | | |
|---|---|---|---|
| Bias | #9 | Describe any efforts to address potential sources of bias | n/a (data collected by UK Biobank) |
| Study size | #10 | Explain how the study size was arrived at | 6 |
| Quantitative variables | #11 | Explain how quantitative variables were handled in the analyses. If applicable, describe which groupings were chosen, and why | 6-7 |
| Statistical methods | #12a | Describe all statistical methods, including those used to control for confounding | 8 |
| Statistical methods | #12b | Describe any methods used to examine subgroups and interactions | 8 |
| Statistical methods | #12c | Explain how missing data were addressed | 8 |
| Statistical methods | #12d | If applicable, describe analytical methods taking account of sampling strategy | 8 |
| Statistical methods | #12e | Describe any sensitivity analyses | 8 |

**Results**

| | | | |
|---|---|---|---|
| Participants | #13a | Report numbers of individuals at each stage of study—eg numbers potentially eligible, examined for eligibility, confirmed eligible, included in the study, completing | 9 |

For peer review only

| | | | |
|---|---|---|---|
| | | follow-up, and analysed. Give information separately for for exposed and unexposed groups if applicable. | |
| Participants | #13b | Give reasons for non-participation at each stage | 9 |
| Participants | #13c | Consider use of a flow diagram | n/a (not applicable here) |
| Descriptive data | #14a | Give characteristics of study participants (eg demographic, clinical, social) and information on exposures and potential confounders. Give information separately for exposed and unexposed groups if applicable. | 9 |
| Descriptive data | #14b | Indicate number of participants with missing data for each variable of interest | 9 |
| Outcome data | #15 | Report numbers of outcome events or summary measures. Give information separately for exposed and unexposed groups if applicable. | 10-13 |
| Main results | #16a | Give unadjusted estimates and, if applicable, confounder-adjusted estimates and their precision (eg, 95% confidence interval). Make clear which confounders were adjusted for and why they were included | 10-13 |
| Main results | #16b | Report category boundaries when continuous variables were categorized | 10-13 |

For peer review only

| | | | |
|---|---|---|---|
| Main results | #16c | If relevant, consider translating estimates of relative risk into absolute risk for a meaningful time period | 10-13 |
| Other analyses | #17 | Report other analyses done—e.g., analyses of subgroups and interactions, and sensitivity analyses | 13 |
| **Discussion** | | | |
| Key results | #18 | Summarise key results with reference to study objectives | 13 |
| Limitations | #19 | Discuss limitations of the study, taking into account sources of potential bias or imprecision. Discuss both direction and magnitude of any potential bias. | 15 |
| Interpretation | #20 | Give a cautious overall interpretation considering objectives, limitations, multiplicity of analyses, results from similar studies, and other relevant evidence. | 14 |
| Generalisability | #21 | Discuss the generalisability (external validity) of the study results | 16-17 |
| **Other Information** | | | |
| Funding | #22 | Give the source of funding and the role of the funders for the present study and, if applicable, for the original study on which the present article is based | 17 |

Notes:

- 6a: n/a (data collected by UK Biobank)
- 9: n/a (data collected by UK Biobank)

For peer review only

- 13c: n/a (not applicable here) The STROBE checklist is distributed under the terms of the Creative Commons Attribution License CC-BY. This checklist was completed on 25. March 2020 using https://www.goodreports.org/, a tool made by the EQUATOR Network in collaboration with Penelope.ai

For peer review only

BMJ Open

# Patterns of multimorbidity and their effects on adverse outcomes in rheumatoid arthritis: a study of 5658 UK Biobank participants

| | |
|---|---|
| Journal: | *BMJ Open* |
| Manuscript ID | bmjopen-2020-038829.R1 |
| Article Type: | Original research |
| Date Submitted by the Author: | 20-Aug-2020 |
| Complete List of Authors: | McQueenie, Ross; University of Glasgow, GPPC, School of Medicine, Dentistry and Nursing, MVLS; NHS Scotland, Public Health Scotland<br>Nicholl, Barbara; University of Glasgow, General Practice and Primary Care<br>Jani, Bhautesh; University of Glasgow, General Practice and Primary Care<br>Canning, Jordan; University of Glasgow, General Practice and Primary Care<br>MacDonald, Sara; University of Glasgow,<br>McCowan, Colin ; University of St. Andrews, School of Medicine<br>Neary, Joanne; University of Glasgow, General Practice and Primary Care<br>Browne, Susan; University of Glasgow, General Practice and Primary Care<br>Mair, Frances; University of Glasgow, General Practice and Primary Care<br>Siebert, Stefan; University of Glasgow, Institute of Infection, Immunity and Inflammation; NHS Greater Glasgow and Clyde, |
| <b>Primary Subject Heading</b>: | Rheumatology |
| Secondary Subject Heading: | Epidemiology |
| Keywords: | RHEUMATOLOGY, CARDIOLOGY, EPIDEMIOLOGY |
| | |

SCHOLARONE™ Manuscripts

*I, the Submitting Author has the right to grant and does grant on behalf of all authors of the Work (as defined in the below author licence), an exclusive licence and/or a non-exclusive licence for contributions from authors who are: i) UK Crown employees; ii) where BMJ has agreed a CC-BY licence shall apply, and/or iii) in accordance with the terms applicable for US Federal Government officers or employees acting as part of their official duties; on a worldwide, perpetual, irrevocable, royalty-free basis to BMJ Publishing Group Ltd ("BMJ") its licensees and where the relevant Journal is co-owned by BMJ to the co-owners of the Journal, to publish the Work in this journal and any other BMJ products and to exploit all rights, as set out in our licence.*

*The Submitting Author accepts and understands that any supply made under these terms is made by BMJ to the Submitting Author unless you are acting as an employee on behalf of your employer or a postgraduate student of an affiliated institution which is paying any applicable article publishing charge ("APC") for Open Access articles. Where the Submitting Author wishes to make the Work available on an Open Access basis (and intends to pay the relevant APC), the terms of reuse of such Open Access shall be governed by a Creative Commons licence – details of these licences and which Creative Commons licence will apply to this Work are set out in our licence referred to above.*

*Other than as permitted in any relevant BMJ Author's Self Archiving Policies, I confirm this Work has not been accepted for publication elsewhere, is not being considered for publication elsewhere and does not duplicate material already published. I confirm all authors consent to publication of this Work and authorise the granting of this licence.*

For peer review only

*Patterns of multimorbidity and their effects on adverse outcomes in rheumatoid arthritis: a study of 5658 UK Biobank participants*

Ross McQueenie[1,2]

Barbara I Nicholl[1]

Bhautesh Dinesh Jani[1]

Jordan Canning[1]

Sara Macdonald[1]

Colin McCowan[3]

Joanne Neary[1]

Susan Browne[1]

Frances S Mair[1*]

Stefan Siebert[4*]

[1] Ross McQueenie BSc (Hons), PhD; Barbara Nicholl BSc, PhD; Bhautesh Dinesh Jani MRCGP, PhD; Jordan Canning BSc (Hons), MRes; Sara Maconald PhD; Joanne Neary BA (Hons), MRes, PhD; Susan Browne MA(Hons), Mphil, PhD; Frances Mair MD, DRCOG, FRCGP: General Practice and Primary Care, Institute of Health and Wellbeing, MVLS, University of Glasgow, UK

[2] Ross McQueenie BSc (Hons), PhD, NHS Public Health Scotland

[3] Colin McCowan PhD: School of Medicine, University of St Andrews, UK

[4] Stefan Siebert MD, FRCP, PhD: Institute of Infection, Immunity and Inflammation, MVLS, University of Glasgow, UK

*Joint Senior author

Stefan Siebert (corresponding author), Institute of Infection, Immunity and Inflammation University of Glasgow, G12 9LX

For peer review only

email: stefan.siebert@glasgow.ac.uk

No author received financial support for the work reported in this manuscript, and all others report no other financial interests that would be considered a conflict of interest.

*Abstract*

*Objective*

To investigate how type and number of long-term conditions (LTCs) impact on all-cause mortality and major adverse cardiovascular events (MACE) in people with rheumatoid arthritis (RA).

*Design*

Population-based longitudinal cohort study.

*Setting*

UK Biobank.

*Participants*

UK Biobank participants (N=502,533) aged between 37 and 73 years old.

*Primary outcome measures*

Primary outcome measures were risk of all-cause mortality and MACE.

*Methods*

We examined the relationship between LTC count and individual comorbid LTCs (N=42) on adverse clinical outcomes in participants with self-reported RA (N=5658). Risk of all-cause mortality and MACE were compared using Cox's proportional hazard models adjusted for

For peer review only

lifestyle factors (smoking, alcohol intake, physical activity), demographic factors (sex, age, socioeconomic status), and rheumatoid factor.

*Results*

75.7% of participants with RA had multimorbidity and these individuals were at increased risk of all-cause mortality and MACE. RA and $\geq$4LTCs showed a three-fold increased risk of all-cause mortality (hazard ratio (HR) 3.30, 95% confidence interval (CI) 2.61-4.16), and MACE (HR 3.45, 95% CI 2.66-4.49) compared to those without LTCs. Of the comorbid LTCs studied, osteoporosis was most strongly associated with adverse outcomes in participants with RA compared to those without RA or LTCs: two-fold increased risk of all-cause mortality (HR 2.20, 95% CI 1.55-3.12) and three-fold increased risk of MACE (HR 3.17, 95% CI 2.27-4.64). These findings remained in a subset (N=3683) with RA diagnosis validated from clinical records or medication reports.

*Conclusion*

Those with RA and other LTCs, particularly comorbid osteoporosis, are at increased risk of adverse outcomes, although the role of corticosteroids could not be evaluated in this study. These results are clinically relevant for the monitoring and management of RA across the healthcare system, and future clinical guidelines for RA should acknowledge the importance of multimorbidity.

*Keywords*

Rheumatoid arthritis, mortality, multimorbidity, comorbidity, cardiovascular

For peer review only

For peer review only - http://bmjopen.bmj.com/site/about/guidelines.xhtml

*Strengths and limitations*

- This is the first study to examine both comorbidity and multimorbidity in RA and the associations with mortality and major adverse cardiovascular events (MACE).
- We used data from 5658 participants in UK Biobank with RA, including detailed information on participant demographics, lifestyle factors and rheumatoid factor status to examine multimorbidity and comorbidity using 42 non-RA LTCs.
- These results provide crucial new information which should be incorporated into clinical guidelines and used to influence management of peoples with RA.
- This study was limited by lack of information on RA disease severity which may play a role in both outcomes measured.

For peer review only

*Introduction*

Rheumatoid arthritis (RA) is a debilitating, chronic autoimmune disease characterised by inflammation of the synovial joints. RA is associated with physical and socio-economic issues, including increased pain levels, reduced physical functioning, and early mortality.[1-4] Globally, whilst disability adjusted life years for RA have improved since 1990, age-standardised prevalence and incidence rates are increasing.[5]

Between 60% and 75% of those with RA are reported to have multimorbidity – two or more long-term conditions (LTCs) - with higher number of LTCs reported with increasing age and disease activity.[6-8] Common comorbidities include cardiovascular conditions[9] such as coronary artery disease[10] and cardiac failure,[11] as well as mental health conditions such as depression.[12] Cardiovascular disease (CVD) accounts for the majority of the excess mortality observed in RA, with raised inflammatory markers and shared risk factors implicated.[13] However, the effects of comorbidities in RA have generally been studied in isolation and less is known regarding the risks posed by multimorbidity when RA co-occurs with more than one other long-term physical or mental health LTC.

Through analysis of UK Biobank data, this paper aims to explore the effect of multimorbidity and a wide range of comorbid LTCs on all-cause mortality and major adverse cardiovascular events (MACE) in people with RA. Our objectives were to:

1. Compare the effect of LTC count on all-cause mortality in those with and without self-reported RA.

2. Compare the effect of LTC count on MACE in those with and without self-reported RA.

For peer review only

3. Evaluate the effect of individual co-morbid LTCs on the risk of all-cause mortality and MACE in participants with self-reported RA.

*Patients and Methods*

*Study design and data collection*

This study utilised data from UK Biobank, a longitudinal population-based cohort of 502533 participants, aged 37-73 years in Great Britain[14]. UK Biobank baseline data was collected between 2006-10 from recruitment centres in Scotland, England and Wales, and subsequently linked to mortality and hospitalisation outcomes from external routine data registries over a median follow-up period of 9 years. A subset of primary care data was available for 230105 participants. This study was covered by the generic ethics approval for UK Biobank studies from the NHS National Research Ethics Service (16/NW/0274).

*Variables and outcome measures*

UK Biobank collected information on a wide range of demographic, health-based lifestyle and self-reported LTC questions through self-administered touch screen questionnaire and nurse-led interview. These include age, sex, socioeconomic status (measured using Townsend score, a UK area-based measure of deprivation),[15] smoking status, frequency of alcohol intake, body mass index (BMI), level of physical activity and number of LTCs.

The age range of the study population was 37-73 years and was categorised into groups: 37-49, 50-59 and 60-73 years. Sex was a binary categorical variable. Smoking status was categorised into "never" or "current or previous". Frequency of alcohol intake was categorised into four groups, "Never or special occasions only", "One to three times a month", "One to four times a week" or "Daily or almost daily". BMI was categorised into four groups based on WHO BMI guidelines [16]: "underweight <18.5", "normal weight 18.5-24.9", "overweight 25-29.9" and "obese ≥30". Level of physical activity was defined as "none",

For peer review only

"low", "medium", or "high" using Metabolic Equivalent Task (MET) scores data based on International Physical Activity Questionnaire (IPAQ) scoring protocol (available from https://sites.google.com/site/theipaq/scoring-protocol) which has shown moderate to good validity and reliability in adults in UK settings.[17, 18]

Rheumatoid factor was ascertained, as part of a predefined biomarker panel, for all participants in UK Biobank, regardless of diagnosis, and categorised into positive and negative status, with rheumatoid factor <20IU/ml considered negative, and values above this considered positive (by manufacturer specification, available at https://www.beckmancoulter.com/wsrportal/techdocs?docname=/cis/988646/%%/RF_988646-%25%25_English.pdf). Participants whose rheumatoid factor was labelled as "not reportable at assay (too low)" were considered to be rheumatoid factor negative. Similarly, those labelled "not reportable at assay (too high)" were considered rheumatoid factor positive.

The list of 42 LTCs considered was based on previous work in UK Biobank,[19, 20] the number of LTCs reported, apart from RA, were summed and then categorised as 0, 1, 2-3 and ≥4 LTCs. RA and all LTCs in UK Biobank are based on self-report using a questionnaire and nurse-led interview asking for existing diagnoses.

All-cause mortality was calculated using data linkage to national mortality registers. MACE were calculated using stroke and myocardial infarction (MI) hospitalisation event data from UK Biobank, and using ICD-10 mortality codes: "I00-I78", "G45", "G451-G454", "G456", "G458", "G459", and "G460-G468". The median follow-up time for both morality and MACE was nine years; the length of follow-up for each participant varied as follow-up continued until an event occurred (death or MACE) or until the mortality the linkage was carried out.

For peer review only

For peer review only - http://bmjopen.bmj.com/site/about/guidelines.xhtml

A sensitivity analysis of self-report RA by participants was performed by examining four other indicators of RA: any primary care RA Read code, any secondary care RA hospitalisation code, self-reporting of any common RA drugs or any primary care prescription record of RA drugs (as shown in Supplementary Table 1). Both prospective and retrospective data were used: primary care Read codes were available for a maximum period of January 1991 and December 2017, and primary care prescriptions were between January 1991 and December 2016; the time period for each participant varied, depending on records held. Participants were considered to have confirmed RA if they had a positive record for one or more of these indicators. This analysis was performed on a subset (74%) of participants who self-reported RA for whom primary care data in UK Biobank was available (N=4196/5658).

*Statistical methods*

In line with previous UK Biobank studies, $\chi 2$ tests were utilised for both categorical data and ordinal data. Kruskal–Wallis tests were used for continuous data.[21] Similarly, we used $\chi 2$ testing to examine differences in proportion of individual LTCs between those with and without RA. Age-adjusted Cox's proportional hazards tests were used to examine the relationship between LTC count / type of LTCs with all-cause mortality and MACE as outcome variables in those with and without RA. The model was further adjusted for demographic, lifestyle and biological factors (sex, Townsend score, alcohol status, smoking status, BMI, physical activity and rheumatoid factor status) as described above. Among those with RA, cumulative hazards-based Kaplan-Meier plots were used to display proportion of events (all-cause mortality or MACE) in participants with 0, 1, 2-3 and ≥4 co-morbid LTCs. To measure the contribution of individual index LTCs towards all-cause mortality and MACE in those with and without RA, we created a categorical variable that assigned participants to one of four groups: those with neither RA nor the index condition (reference

For peer review only

group), those with RA but not the index LTC (RA only), those with no RA with the index LTC (index LTC only), and those with both RA and the index LTC. This variable was used as an outcome measure in an age-adjusted Cox's proportional hazards model controlling for demographic factors, lifestyle factors and rheumatoid factor status. To calculate whether there was a multiplicative or synergistic effect between RA and each index LTC, we used an ANOVA to compare the p-values between two Cox's proportional hazards models: the first contained RA and the index LTC, and the second contained RA, the index LTC and a statistical interaction term between RA and the index LTC. A statistical interaction was considered significant when the ANOVA test has a $p<0.01$.

*Results*

5658 UK Biobank participants (1.1%) reported having RA. Lifestyle and demographic characteristics of participants with and without self-reported RA are shown in Table 1. Participants with RA were significantly more likely to be older, female, have lower socioeconomic status, be current or previous smokers, have a lower frequency of alcohol intake, have a BMI ≥30, have lower levels of physical activity, and have larger numbers of co-morbid LTCs. $\chi2$ testing showed participants with self-reported RA were significantly more likely to have rheumatoid factor positive status: 35.6% had rheumatoid factor levels of over 20 IU/ml – compared with 3.6 % in those without RA.

*Prevalence of LTCs in people with RA*

Proportions of number of LTCs in participants with and without RA are shown in Table 1. Reporting multiple long-term conditions was more common in those with RA: 34.5% had 2-3 LTCs (27.1% in those without RA), and 11.1% had ≥ 4 LTCs (4.9% in those without RA). Overall, 75.7% of people with RA were noted to be multimorbid. The difference in comorbidity experienced by those with and without RA is shown in Supplementary Table 2.

For peer review only

Those with RA reported proportionately higher numbers of physical and mental health-based LTCs, namely: cardiovascular LTCs including hypertension, coronary heart disease, and stroke or transient ischemic attack; pulmonary LTCs including asthma, COPD and chronic bronchitis; digestive system LTCs including dyspepsia, irritable bowel syndrome and inflammatory bowel disease; musculoskeletal conditions including osteoporosis; and mental-health based LTCs including depression.

*All-cause mortality and LTCs in people with RA*

We examined the outcomes associated with different LTC counts in participants with RA using a Kaplan Meier plot (Supplementary Figure 1). There was an increased proportion of all-cause mortality in participants with RA concurrent with increasing multimorbidity counts: 4.2% (N=58) in those with no additional LTCs, 5.3% (N=91) in those with 1 additional LTC, 9.9% (N=194) in those with 2-3 additional LTCs and 14.4% (N=90) in those with ≥4 additional LTCs during the follow up period (median 9 years).

To quantify the effect of LTC count on all-cause mortality, we performed a Cox's proportional hazards test controlling for lifestyle factors, demographic factors and rheumatoid factor in participants with and without self-reported RA using a stepwise model adjustment (Table 2). Participants with RA and no additional LTCs had a significant increase in all-cause mortality when using an age-adjusted Cox's proportional hazards model fully adjusting for additional lifestyle and demographic factors (Hazard Ratio (HR) 1.59, 95% confidence intervals (CI) 1.21-2.08) compared to those without RA or any LTCs. Whilst controlling additionally for rheumatoid factor status appeared to show some attenuation of all-cause mortality risk, a statistically significant risk for this group remained (HR 1.39, 95% CI 1.05-1.84) when compared to those without RA or any LTCs. When examining additional co-morbid LTCs alongside RA, there appeared to be a dose-based response all-cause mortality

For peer review only

risk, with a 44% increased risk of all-cause mortality in those with RA and one other LTC (HR 1.44, 95% CI 1.14-1.81), an approximately two-and-a-half-fold increased risk for RA with 2-3 other LTCs (HR 2.48, 95% CI 2.12-2.90) and an over three-fold increased risk associated for RA with ≥4 other LTCs (HR 3.30, 95% CI 2.61-4.16) compared to those without RA or any LTCs in the fully adjusted models, which included rheumatoid factor. A dose-based response was also observed in the non-RA population: those with 1 LTC had a 39% increased risk of death (HR 1.39, 95% CI 1.33-1.46), and those with ≥4 were at a two-and-a-half-fold increased risk (HR 2.69 95% CI 2.54-2.85) compared with participants without RA or any LTCs.

*MACE and LTCs in people with RA*

We next investigated the effect of LTC count on MACE in participants with RA using a Kaplan Meier plot (Supplementary Figure 2). For RA and no additional LTCs, 3.3% (N=46) of participants had a recorded MACE event, compared with 4.6% of participants with RA and one additional LTC (N=78), 6.7% those with RA and 2-3 additional LTCS (N=131), and almost four times as many proportionately in participants with RA and ≥4 LTCs (11.7%, N=73 events) over the follow-up period.

Table 3 shows the risk of MACE for participants with and without RA using age-adjusted multivariate Cox's proportional hazards regression models. There was a 63% increased hazard of MACE for participants with RA and no other LTCs compared with participants without RA or any LTCs (HR 1.63, 95% CI 1.21-2.21) in a fully adjusted model including demographic factors, lifestyle factors and rheumatoid factor status. This remained significant for people with RA with increasing LTCs count, with a 86% increased risk of MACE in participants with one other co-occurring LTC (HR 1.86, 95% CI 1.31-2.15), an over two-fold increase in those with 2-3 co-occurring LTCs (HR 2.09, 95% CI 1.73-2.54) and an almost three-and-a-

For peer review only

half-fold increase in MACE for those with ≥4 LTCs (HR 3.39, 95% CI 2.61-4.40), compared to those without RA or any LTCs. This relationship was similar but to a lesser degree for participants without RA, with those with 1 LTC at 24% increased risk (HR 1.24, 95% CI 1.19-1.31), those with 2-3 LTCs at a 66% increased risk (HR 1.66, 95% CI 1.59-1.74) and those with ≥4 LTCs at over two times risk (HR 2.37 95% CI 2.23-2.53) of MACE compared with those without LTCs.

A similar pattern was observed for the relationship between LTC count and mortality/MACE for the group without RA (Supplementary Figures 3 and 4).

*Contribution of individual LTCs to all-cause mortality and MACE in people with RA*

Using an age-adjusted Cox's proportional hazards model, adjusting for demographic factors, lifestyle factors and rheumatoid factor status, we investigated the role individual LTCs play in risk of all-cause mortality and MACE, using participants with no RA and no index condition as the reference group (Table 4 and 5).

The presence of cardiovascular-based LTCs appeared to be a risk factor in those with RA for both all-cause mortality and MACE. Compared to those with no RA and no hypertension, RA with hypertension showing an over one-and-a-half-fold increased risk of all-cause mortality (HR 1.59, 95% CI 1.37-1.86) and an approximately two-fold increased risk of MACE (HR 2.07, 95% CI 1.64-2.33).

Similarly, heart disease was associated with an over two-fold increase for both all-cause mortality (HR 2.07, 95% CI 1.63-2.63) and MACE (HR 2.28 95% CI 1.76-2.98) in those with RA compared to those with no RA and no heart disease. However, there was no evidence of interaction between RA and either cardiovascular condition. Whilst thyroid disorders showed no significant increased risk of all-cause mortality, they displayed an over two-fold increased

risk of MACE (HR 2.10, 95% CI 1.50-2.93) in those with RA compared to those without RA or thyroid disease but again there was no significant interaction between RA and thyroid disease and MACE event.

The co-occurrence of osteoporosis in participants with RA appeared to strongly influence both mortality and MACE; more than doubling all-cause mortality (HR 2.20, 95% CI 1.55-3.12), and resulting in an over three times higher risk of MACE (HR 3.17, 95% CI 2.17-4.64) compared to those without RA or osteoporosis. This increased risk in those with both RA and osteoporosis was greater than in those with RA but no osteoporosis or those with osteoporosis but no RA. Interaction terms for RA and osteoporosis showed no significant interaction with all-cause mortality ($p=0.10$), suggesting an additive effect only, but displayed a significant interaction with MACE ($p<0.01$), suggesting a multiplicative or synergistic effect in the association with MACE.

*Sensitivity analysis of RA self-report*

To investigate sensitivity of self-report by participants with RA, we examined the proportion of people with any primary care RA Read code, any secondary care RA hospitalisation code, self-reporting of any common RA drugs and any primary care prescription record of RA drugs (see supplementary table 1) for participants who had self-reported RA and had available primary care data available in UK Biobank (N=4196). Medications used here were previously reported by Siebert et al.[21] Using this method, we were able to identify RA medications, hospitalisations or primary care Read code in 3683 (87.8%) participants (Supplementary Table 3). Analysis performed in this study was repeated in these participants and showed the same relationships as those reported above in N=5658 with self-report RA, with only small changes in HR observed (Supplementary Tables 4-8).

*Discussion*

Within UK Biobank, multiple LTCs was common in participants with RA, with approximately 75.7% reporting multimorbidity and 45% of participants reporting two or more additional LTCs alongside RA. In our fully adjusted modes, increasing LTC count was associated with increased mortality and MACE in people with RA. When examining individual LTCs, we observed hypertension, heart disease, osteoporosis and thyroid disorders to increase risk of adverse outcomes. Of these, osteoporosis was associated with one of the largest increases in both adverse outcomes measured: participants with both RA and osteoporosis were at over three times the risk of all-cause mortality and two times the risk of compared to those with neither LTC. The negative effect of having both RA and osteoporosis was particularly evident in MACE outcomes, for which there was a significant interaction between RA and osteoporosis, suggesting a multiplicative or synergistic effect on MACE of having both these conditions together. The presence of hypertension or heart disease alongside RA increased the risk of mortality and MACE, in keeping with previous literature,[22, 23] but there was no evidence of a synergistic effect.

To the best of our knowledge, this paper is the first to compare LTC count and type of comorbid LTCs and their association with all-cause mortality and MACE in men and women with RA after adjusting for a wide range of sociodemographic and lifestyle variables along with rheumatoid factor status. In our study, increasing LTC count resulted in adverse outcomes in participants with RA, with an increased rate of all-cause mortality and MACE.

We have shown that multimorbidity is common in participants with RA, with around 75% of participants with RA reporting one or more additional LTCs. This is in agreement with reported comorbidity rates of between 60% and 75% in those with RA,[6-8] although these studies typically examined a smaller number of LTCs than in this study. We have shown participants with RA and 2-3 other LTCs were at over twice the risk of all-cause mortality, whilst those with ≥ 4 more were over three times the risk compared to participants with no

For peer review only

LTCs. This data provides evidence for the first time the increased risk of all-cause mortality in men and women with RA and multimorbidity. While previous work has highlighted an increased risk of mortality in RA patients,[24, 25] or specific comorbidities alongside RA – for example in COPD[26] and depression[27] – these studies did not examine the effect of LTC count. One matched cohort study used a multimorbidity weighted index to study the effect of multimorbidity on mortality, but only examined effects in women.[28] Another examined LTCs using the Charlson comorbidity index,[29] however this measure uses only 19 LTCs and the study examined only all-cause mortality outcomes. Our study is the first study of its type to link multimorbidity in RA with MACE outcomes. Existing research has highlighted that RA increases the risk of cardiovascular events, and that individual LTCs such as diabetes and hypertension are risk factors,[30] however, to date, no study has shown an association between multimorbidity and MACE outcomes in people with RA. Collectively, the results presented here report for the first time the magnitude of adverse outcomes associated with multimorbidity in those with RA.

In keeping with previous studies,[8, 31] we have shown that osteoporosis prevalence is increased in those with RA compared to those without RA. The results presented in this paper, however, are the first to link osteoporosis in those with RA to increased risk of adverse outcomes and the first to show significant interaction between both conditions and MACE outcomes. The reasons for this association are not clear and cannot be extrapolated from the available data, which does not include factors such as disease severity or duration. One possibility may be that corticosteroids and RA disease activity play a role: corticosteroids are associated with increased prevalence of osteoporosis[32]; people with RA with higher levels of disease activity are more likely to receive corticosteroids; both corticosteroid use and increased RA disease activity are reported to be associated with worse outcomes in mortality and MACE.[33, 34]

For peer review only

Our study therefore has several strong clinical implications. Current NICE guidelines for RA suggest annual checks for the development of hypertension, ischemic heart disease, osteoporosis and depression in RA,[35] but do not highlight the increased risk of the co-occurrence of these LTCs with RA nor the risk posed by multimorbidity in general. In addition, we have shown a greatly increased risk of adverse outcomes in people with osteoporosis and RA that merits further investigation.

Our study has several key strengths: UK Biobank is a large population-based study with several thousand participants reporting RA; the study setting encompasses three countries within the UK (Scotland, England and Wales); it includes details of participant demographic and lifestyle factors as well as rheumatoid factor levels, which allowed us to adjust for variables, which have not been explored in previous studies.

Our study is limited by self-reporting of RA and LTCs by these participants; however, recent studies have shown that self-report is a reliable method for reporting RA[36]. Inthis study we additionally used four RA indicators (any primary care RA Read code, any secondary care RA hospitalisation code, self-reporting of any common RA drugs and any primary care prescription record of RA drugs) to validate self-reported RA. Using this validation approach, we found a positive verification rate (participants self-reporting RA with further RA indicators) of 87.8% (N=3683). Re-analysis of the subset of participants with RA (Supplementary Tables 4-8) who had a validated RA report showed only small changes to Cox's proportional hazards models, and observed effects were in agreement with the population who self-reported RA. This provides confidence in our findings that we are examining a true RA population. Rheumatoid factor positive status in those self-reporting RA (35.6%) was lower than expected, however still a significantly higher proportion than in the UK Biobank population who did not report RA (3.6%). Analysis of rheumatoid factor in those who had a validated RA report showed an increased proportion of positive rheumatoid

For peer review only

factor (47.6%), but this level remained below previously reported proportions in RA populations. We were unable to determine the severity or duration of RA in participants, or their previous medications. Participants in UK Biobank are known to be less deprived than the wider UK population,[37] suggesting that the level of multimorbidity reported here; and resulting associations are likely to be conservative in nature. Future work will examine potential clusters of LTCs that are associated with poor health-related outcomes in people with RA to try to inform clinical management of patients with RA and multiple LTCs.

*Conclusions*

Multimorbidity is common in people with RA and is associated with increased risk of all-cause mortality and MACE. Certain comorbidities such as osteoporosis merit specific attention, in view of their association with adverse outcomes; it will be important to test whether this association is replicated in other datasets and if so, to explore the underpinning mechanisms. As multimorbidity has been shown here to influence outcomes for those with RA, forthcoming work will examine which clusters of LTCs most strongly drive this increased risk of poor outcomes. Future clinical guidelines for RA should acknowledge the importance of multimorbidity when considering management planning and patient outcomes.

Word count: 3683 words

*Acknowledgements*

This research has been conducted using the UK Biobank Resource, approved project number 14151; we are like to thank the participants and those managing the data. We would like to acknowledge Versus Arthritis for funding this project.

*Ethical approval*

For peer review only

All participants gave informed consent for data provision and linkage. UK Biobank has full ethical approval from the NHS National Research Ethics Service (16/NW/0274).

*Competing interests*

None declared.

*Funding*

This study was funded by Versus Arthritis (grant number 21970)

*Data sharing statement*

The data used in this study are available via a direct application to UK Biobank.

*Author contributions*

This study was conceived by BN, FSM, SS, BJ and CM. The analysis was conducted by RM, BN and BJ. All authors (RM, BJ, BN, JC, SM, CM, JN, SB, FSM, SS) contributed to design, interpretation and discussion of all analysis. RM wrote this manuscript. All authors (RM, BJ, BN, JC, SM, CM, JN, SB, FSM, SS) edited, reviewed and commented on all versions of this manuscript. All authors read the manuscript draft and approved the final submission.

*Patient and Public Involvement*

The study was supported by a patient advisory group which provided input to the programme of research. This patient advisory group met on a regular basis for the duration of the study. Patients partnered with us and helped design research questions.

*References*

1. Andrews JS, Trupin L, Yelin EH, et al. Frailty and reduced physical function go hand in hand in adults with rheumatoid arthritis: a US observational cohort study. *Clinical rheumatology* 2017;**36**:1031-39. doi: 10.1007/s10067-017-3541-9
2. Sokka T, Abelson B, Pincus T. Mortality in rheumatoid arthritis: 2008 update. *Clinical and experimental rheumatology* 2008;**26**:S35-61.

For peer review only

3. Humphreys JH, Warner A, Chipping J, et al. Mortality trends in patients with early rheumatoid arthritis over 20 years: results from the Norfolk Arthritis Register. *Arthritis care & research* 2014;**66**:1296-301. doi: 10.1002/acr.22296

4. Gwinnutt JM, Verstappen SM, Humphreys JH. The impact of lifestyle behaviours, physical activity and smoking on morbidity and mortality in patients with rheumatoid arthritis. *Best practice & research Clinical rheumatology* 2020:101562. doi: 10.1016/j.berh.2020.101562

5. Safiri S, Kolahi AA, Hoy D, et al. Global, regional and national burden of rheumatoid arthritis 1990–2017: a systematic analysis of the Global Burden of Disease study 2017. *Annals of the Rheumatic Diseases* 2019;**78**:1463. doi: 10.1136/annrheumdis-2019-215920

6. Espino-Lorenzo P, Manrique-Arija S, Urena I, et al. Baseline comorbidities in patients with rheumatoid arthritis who have been prescribed biological therapy: a case control study. *Reumatologia clinica* 2013;**9**:18-23. doi: 10.1016/j.reuma.2012.05.012

7. Gron KL, Ornbjerg LM, Hetland ML, et al. The association of fatigue, comorbidity burden, disease activity, disability and gross domestic product in patients with rheumatoid arthritis. Results from 34 countries participating in the Quest-RA program. *Clinical and experimental rheumatology* 2014;**32**:869-77.

8. Luque Ramos A, Redeker I, Hoffmann F, et al. Comorbidities in Patients with Rheumatoid Arthritis and Their Association with Patient-reported Outcomes: Results of Claims Data Linked to Questionnaire Survey. *The Journal of rheumatology* 2019;**46**:564-71. doi: 10.3899/jrheum.180668

9. Løgstrup BB, Olesen KKW, Masic D, et al. Impact of rheumatoid arthritis on major cardiovascular events in patients with and without coronary artery disease. *Ann Rheum Dis* 2020 doi: 10.1136/annrheumdis-2020-217154

10. Solomon DH, Goodson NJ, Katz JN, et al. Patterns of cardiovascular risk in rheumatoid arthritis. *Ann Rheum Dis* 2006;**65**:1608-12. doi: 10.1136/ard.2005.050377

11. Nicola PJ, Maradit-Kremers H, Roger VL, et al. The risk of congestive heart failure in rheumatoid arthritis: a population-based study over 46 years. *Arthritis and rheumatism* 2005;**52**:412-20. doi: 10.1002/art.20855

12. Matcham F, Rayner L, Steer S, et al. The prevalence of depression in rheumatoid arthritis: a systematic review and meta-analysis. *Rheumatology (Oxford)* 2013;**52**:2136-48. doi: 10.1093/rheumatology/ket169

13. Ferguson LD, Siebert S, McInnes IB, et al. Cardiometabolic comorbidities in RA and PsA: lessons learned and future directions. *Nat Rev Rheumatol* 2019;**15**:461-74. doi: 10.1038/s41584-019-0256-0

14. Sudlow C, Gallacher J, Allen N, et al. UK biobank: an open access resource for identifying the causes of a wide range of complex diseases of middle and old age. *PLoS medicine* 2015;**12**:e1001779. doi: 10.1371/journal.pmed.1001779

15. Townsend PJJosp. Deprivation. 1987;**16**:125-46.

16. Wolfe F. Pain extent and diagnosis: development and validation of the regional pain scale in 12,799 patients with rheumatic disease. *The Journal of rheumatology* 2003;**30**:369-78.

17. Cleland C, Ferguson S, Ellis G, et al. Validity of the International Physical Activity Questionnaire (IPAQ) for assessing moderate-to-vigorous physical activity and sedentary behaviour of older adults in the United Kingdom. *BMC Med Res Methodol* 2018;**18**:176. doi: 10.1186/s12874-018-0642-3

18. Craig CL, Marshall AL, Sjostrom M, et al. International physical activity questionnaire: 12-country reliability and validity. *Med Sci Sports Exerc* 2003;**35**:1381-95. doi: 10.1249/01.MSS.0000078924.61453.FB

19. Jani BD, Nicholl BI, McQueenie R, et al. Multimorbidity and co-morbidity in atrial fibrillation and effects on survival: findings from UK Biobank cohort. *EP Europace* 2017;**20**:f329-f36. doi: 10.1093/europace/eux322

For peer review only

20. Jani BD, Hanlon P, Nicholl BI, et al. Relationship between multimorbidity, demographic factors and mortality: findings from the UK Biobank cohort. *BMC Medicine* 2019;**17**:74. doi: 10.1186/s12916-019-1305-x
21. Siebert S, Lyall DM, Mackay DF, et al. Characteristics of rheumatoid arthritis and its association with major comorbid conditions: cross-sectional study of 502 649 UK Biobank participants. *RMD open* 2016;**2**:e000267.
22. Cooksey R, Brophy S, Kennedy J, et al. Cardiovascular risk factors predicting cardiac events are different in patients with rheumatoid arthritis, psoriatic arthritis, and psoriasis. *Seminars in arthritis and rheumatism* 2018;**48**:367-73. doi: 10.1016/j.semarthrit.2018.03.005
23. Lauper K, Courvoisier DS, Chevallier P, et al. Incidence and Prevalence of Major Adverse Cardiovascular Events in Rheumatoid Arthritis, Psoriatic Arthritis, and Axial Spondyloarthritis. *Arthritis care & research* 2018;**70**:1756-63. doi: 10.1002/acr.23567
24. van den Hoek J, Boshuizen HC, Roorda LD, et al. Mortality in patients with rheumatoid arthritis: a 15-year prospective cohort study. *Rheumatol Int* 2017;**37**:487-93. doi: 10.1007/s00296-016-3638-5
25. Gabriel SE, Crowson CS, Kremers HM, et al. Survival in rheumatoid arthritis: a population-based analysis of trends over 40 years. *Arthritis and rheumatism* 2003;**48**:54-8. doi: 10.1002/art.10705
26. Hyldgaard C, Løkke A, Pedersen AB, et al. Prognosis in rheumatoid arthritis patients with COPD: A nationwide, registry-based study. *European Respiratory Journal* 2017;**50**:OA1786. doi: 10.1183/1393003.congress-2017.OA1786
27. van den Hoek J, Boshuizen HC, Roorda LD, et al. Association of Somatic Comorbidities and Comorbid Depression With Mortality in Patients With Rheumatoid Arthritis: A 14-Year Prospective Cohort Study. *Arthritis care & research* 2016;**68**:1055-60. doi: 10.1002/acr.22812
28. Yoshida K, Lin T-C, Wei M, et al. The roles of post-diagnosis accumulation of morbidities and lifestyle changes on excess total and cause-specific mortality risk in rheumatoid arthritis. *Arthritis care & research* 2019 doi: 10.1002/acr.24120
29. Nikiphorou E, de Lusignan S, Mallen C, et al. Prognostic value of comorbidity indices and lung diseases in early rheumatoid arthritis: a UK population-based study. *Rheumatology* 2019 doi: 10.1093/rheumatology/kez409
30. Dalbeni A, Giollo A, Bevilacqua M, et al. Traditional cardiovascular risk factors and residual disease activity are associated with atherosclerosis progression in rheumatoid arthritis patients. *Hypertens Res* 2020;**43**:922-28. doi: 10.1038/s41440-020-0441-1
31. Lindner L, Callhoff J, Alten R, et al. Osteoporosis in patients with rheumatoid arthritis: trends in the German National Database 2007-2017. *Rheumatol Int* 2020 doi: 10.1007/s00296-020-04593-6
32. Wang Y, Zhao R, Gu Z, et al. Effects of glucocorticoids on osteoporosis in rheumatoid arthritis: a systematic review and meta-analysis. *Osteoporos Int* 2020;**31**:1401-09. doi: 10.1007/s00198-020-05360-w
33. del Rincon I, Battafarano DF, Restrepo JF, et al. Glucocorticoid dose thresholds associated with all-cause and cardiovascular mortality in rheumatoid arthritis. *Arthritis & rheumatology (Hoboken, NJ)* 2014;**66**:264-72. doi: 10.1002/art.38210
34. Listing J, Kekow J, Manger B, et al. Mortality in rheumatoid arthritis: the impact of disease activity, treatment with glucocorticoids, TNFalpha inhibitors and rituximab. *Ann Rheum Dis* 2015;**74**:415-21. doi: 10.1136/annrheumdis-2013-204021
35. National Institute for Health and Care Excellence. Rheumatoid arthritis in adults: management: National Insttute for Health and Care Excellence, 2018.
36. Peeters GM, Alshurafa M, Schaap L, et al. Diagnostic accuracy of self-reported arthritis in the general adult population is acceptable. *J Clin Epidemiol* 2015;**68**:452-9. doi: 10.1016/j.jclinepi.2014.09.019


For peer review only

37. Fry A, Littlejohns TJ, Sudlow C, et al. Comparison of Sociodemographic and Health-Related Characteristics of UK Biobank Participants With Those of the General Population. *American Journal of Epidemiology* 2017;**186**:1026-34. doi: 10.1093/aje/kwx246

*Figure legends*

Supplementary figure 1 – Kaplan-Meier plot of proportion of all-cause mortality during the follow-up period (median 108 months) for participants with RA and no LTCS (black line), RA and 1 LTC (red line), RA and 2-3 LTCs (green line) and RA and ≥4 LTCs (blue line).

Supplementary figure 2 – Kaplan-Meier plot of proportion of MACE during the follow-up period (median 108 months) for participants with RA and no LTCS (black line), RA and 1 LTC (red line), RA and 2-3 LTCs (green line) and RA and ≥4 LTCs (blue line).

Supplementary figure 3 – Kaplan-Meier plot of proportion of all-cause mortality during the follow-up period (median 108 months) for participants no RA and no LTCS (black line), RA no RA and 1 LTC (red line), no RA and 2-3 LTCs (green line) and no RA and ≥4 LTCs (blue line).

Supplementary figure 4 – Kaplan-Meier plot of proportion of MACE during the follow-up period (median 108 months) for participants no RA and no LTCS (black line), RA no RA and 1 LTC (red line), no RA and 2-3 LTCs (green line) and no RA and ≥4 LTCs (blue line).

For peer review only

*Tables*

Table 1 – Demographic factors, lifestyle factors, number of long-term conditions and rheumatoid factor status in patients with and without rheumatoid arthritis. Unless indicated, p<0.01. $\chi2$ test was used for categorical variables, Kruskal-Wallis test was used for continuous variables. SD = standard deviation. Unless otherwise indicated, all results are shown as number (%).

| | Participants with RA (N=5658) | Participants without RA (N=496882) |
|---|---|---|
| **Mean Age (years (SD)); missing values =0 (0%)** | 59.3 (7.1) | 56.5 (8.1) |
| **Age (years); missing values = 0 (0%)** | | |

For peer review only

| | | |
|---|---|---|
| 37-49 | 675 (11.9%) | 117209 (23.6%) |
| 50-59 | 1800 (31.8%) | 165359 (33.3%) |
| 60-73 | 3183 (56.3%) | 214314 (43.1%) |
| **Sex; missing values = 0 (0%)** | | |
| Female | 3952 (69.8%) | 269452 (54.2%) |
| Male | 1706 (30.2%) | 227430 (45.8%) |
| **Townsend score; missing values = 623 (0.12%)** | | |
| 0-20 (least deprived) | 998 (17.7%) | 99665 (20.1%) |
| 20-40 | 980 (17.4%) | 99117 (20%) |
| 40-60 | 1087 (19.2%) | 99311 (20%) |
| 60-80 | 1154 (20.4%) | 99224 (20%) |
| 80-100 (most deprived) | 1429 (25.3%) | 98952 (19.9%) |
| **Smoking status; missing values = 2950 (0.59 %)** | | |
| Never | 2625 (46.8%) | 270916 (54.8%) |
| Current or Previous | 2983 (53.2%) | 223066 (45.2%) |
| **Frequency of alcohol intake; missing values = 1502 (0.30 %)** | | |
| Never or special occasions only | 1830 (32.4%) | 96832 (19.5%) |
| One to three times a month | 690 (12.2%) | 55170 (11.1%) |
| One to four times a week | 2315 (41%) | 242428 (48.9%) |
| Daily or almost daily | 811 (14.4%) | 100962 (20.4%) |
| **BMI (kg/m$^2$); missing values = 5820 (1.15 %)** | | |
| underweight <18.5 | 50 (0.9%) | 2576 (0.5%) |
| normal weight 18.5-24.9 | 1543 (27.9%) | 155896 (31.7%) |
| overweight 25-29.9 | 2194 (39.6%) | 212032 (43.2%) |
| obese ≥30 | 1750 (31.6%) | 120679 (24.6%) |
| **Physical activity; missing values = 7156 (1.42 %)** | | |
| none | 814 (14.8%) | 32035 (6.5%) |
| low | 409 (7.4%) | 18531 (3.8%) |
| medium | 4111 (74.5%) | 389412 (79.5%) |
| high | 182 (3.3%) | 49890 (10.2%) |
| **Number of long-term conditions; missing values = 1845 (0.36 %)** | | |
| 0 | 1369 (24.3%) | 173846 (35.1%) |
| 1 | 1690 (30.0%) | 162657 (32.9%) |
| 2-3 | 1943 (34.5%) | 134403 (27.1%) |
| ≥4 | 623 (11.1%) | 24157 (4.9%) |
| **Rheumatoid Factor (IU/ml); missing values = 33,066 (6.6 %)** | | |
| <20 | 3396 (64.4%) | 447472 (96.4%) |
| >20 | 1879 (35.6%) | 16720 (3.6%) |

For peer review only

For peer review only

Table 2 - Relationship between long term conditions and all-cause mortality in participants with and without self-reported RA using age-adjusted multivariate Cox's proportional hazards regression analysis. Unless otherwise shown, Cox's proportional hazards p<0.01.

| Risk of all-cause mortality | | | | | |
|---|---|---|---|---|---|
| **Comorbidity status (reference: *No RA and no other long-term conditions*)** | **Adjusted for sex and Townsend score HR (95% CI)** | **Adjusted for sex, Townsend score, alcohol status and smoking status** | **Adjusted for sex, Townsend score, alcohol status, smoking status, BMI** | **Adjusted for sex, Townsend score, alcohol status, smoking status, BMI, physical** | **Number of deaths (%)** |

| | | | HR (95% CI) | and physical activity HR (95% CI) | activity and rheumatoid factor status HR (95% CI) | |
|---|---|---|---|---|---|---|
| **No other long-term conditions** | **RA** | 1.84 (1.42-2.38) | 1.72 (1.32-2.2) | 1.59 (1.21-2.08) | 1.39 (1.05-1.84) | 58 (4.2%) |
| **1 other long-term condition** | **No RA** | 1.45 (1.39-1.51) | 1.42 (1.36-1.48) | 1.40 (1.34-1.47) | 1.39 (1.33-1.46) | 5785 (3.6%) |
| | **RA** | 2.01 (1.64-2.48) | 1.88 (1.53-2.32) | 1.72 (1.38-2.14) | 1.44 (1.14-1.81) | 91 (5.4%) |
| **2-3 other long-term conditions** | **No RA** | 2.03 (1.95-2.11) | 1.92 (1.84-2.00) | 1.84 (1.77-1.92) | 1.83 (1.75-1.91) | 7914 (5.9%) |
| | **RA** | 3.32 (2.87-3.84) | 2.99 (2.59-3.46) | 2.79 (2.40-3.24) | 2.48 (2.12-2.90) | 194 (10.0%) |
| **≥4 other long-term conditions** | **No RA** | 3.39 (3.22-3.57) | 3.04 (2.88-3.20) | 2.71 (2.56-2.86) | 2.69 (2.54-2.85) | 2605 (10.8%) |
| | **RA** | 4.68 (3.80-5.78) | 3.95 (3.19-4.89) | 3.52 (2.81-4.40) | 3.30 (2.61-4.16) | 90 (14.4%) |

Table 3 Relationship between long term conditions and major adverse cardiovascular events in participants with and without self-reported RA using age-adjusted multivariate Cox's proportional hazards regression analysis. Unless otherwise shown, Cox's proportional hazards $p<0.01$.

**Risk of MACE**

| **Comorbidity status (reference: *No RA and no other long-term conditions*)** | **Adjusted for sex and Townsend score** | **Adjusted for sex, Townsend score,** | **Adjusted for sex, Townsend score,** | **Adjusted for sex, Townsend score,** | **Number of MACE (%)** |
|---|---|---|---|---|---|

For peer review only

| | | HR (95% CI) | alcohol status and smoking status HR (95% CI) | alcohol status, smoking status, BMI, and physical activity HR (95% CI) | alcohol status, smoking status, BMI, physical activity and rheumatoid factor status HR (95% CI) | |
|---|---|---|---|---|---|---|
| No other long-term conditions | RA | 1.79 (1.33-2.39) | 1.69 (1.26-2.27) | 1.64 (1.21-2.20) | 1.63 (1.21-2.21) | 46 (3.4%) |
| 1 other long-term condition | No RA | 1.30 (1.24-1.36) | 1.28 (1.22-1.34) | 1.26 (1.20-1.320 | 1.24 (1.19-1.31) | 4512 (2.8%) |
| | RA | 2.08 (1.66-2.61) | 1.91 (1.52-2.41) | 1.87 (1.48-2.35) | 1.68 (1.31-2.15) | 78 (4.6%) |
| 2-3 other long-term conditions | No RA | 1.86 (1.78-1.94) | 1.78 (1.70-1.86) | 1.67 (1.60-1.75) | 1.66 (1.59-1.74) | 6208 (4.6%) |
| | RA | 2.72 (2.28-3.24) | 2.49 (2.09-2.98) | 2.19 (1.82-2.64) | 2.09 (1.73-2.54) | 131 (6.7%) |
| ≥4 other long-term conditions | No RA | 3.04 (2.87-3.22) | 2.76 (2.60-2.93) | 2.40 (2.26-2.56) | 2.37 (2.23-2.53) | 1980 (8.2%) |
| | RA | 4.79 (3.79-6.04) | 4.07 (3.21-5.16) | 3.52 (2.73-4.52) | 3.39 (2.61-4.40) | 73 (11.7%) |

For peer review only

Table 4 Risk of all-cause mortality for individual index conditions in patients with RA and no index condition, RA with index condition, RA with no index condition and RA and index condition. Age-adjusted Cox's proportional hazards models were adjusted for sex, Townsend score, smoking status, alcohol intake frequency, BMI, physical activity level and rheumatoid factor status. Cox's proportional hazards p<0·01, except for those labelled with + indicating p>0.01. Index conditions labelled * have interaction term p<0.01

| | Risk of all-cause mortality | | | |
|---|---|---|---|---|
| | No RA, no index condition | No RA, with index condition HR, (95% CI) | RA, no index condition HR, (95% CI) | RA and index condition HR, (95% CI) |

| Index condition | | | | |
|---|---|---|---|---|
| Hypertension | 1 | 1.24 (1.21-1.28) | 1.29 (1.11-1.48) | 1.59 (1.37-1.86) |
| Coronary heart disease | 1 | 1.57 (1.50-1.65) | 1.26 (1.12-1.42) | 2.07 (1.63-2.63) |
| Diabetes | 1 | 1.68 (1.60-1.75) | 1.33 (1.18-1.48) | 1.83 (1.37-2.44) |
| Asthma | 1 | 1.10 (1.05-1.15) | 1.27 (1.13-1.42) | 1.56 (1.22-2.00) |
| Dyspepsia | 1 | 1.01 (0.97-1.06)$^{+}$ | 1.27 (1.14-1.43) | 1.45 (1.10-1.90) |
| Cancer | 1 | 2.50 (2.41-2.59) | 1.35 (1.20-1.52) | 3.04 (2.39-3.86) |
| Depression | 1 | 1.27 (1.20-1.35) | 1.29 (1.15-1.44) | 1.71 (1.21-2.42) |
| Thyroid disorder | 1 | 1.05 (0.98-1.12)$^{+}$ | 1.32 (1.18-1.47) | 1.14 (0.80-1.62)$^{+}$ |
| COPD | 1 | 2.11 (1.98-2.49) | 1.26 (1.13-1.42) | 2.68 (2.00-3.58) |
| Epilepsy | 1 | 1.81 (1.42-1.82) | 1.29 (1.15-1.43) | 2.86 (1.43-5.73) |
| Migraine | 1 | 0.85 (0.76-0.94) | 1.29 (1.16-1.44) | 1.09 (0.55-2.19)$^{+}$ |
| Psoriasis/Eczema | 1 | 1.05 (0.98-1.14)$^{+}$ | 1.27 (1.14-1.42) | 1.88 (1.20-2.95) |
| Prostate disease | 1 | 0.83 (0.76-0.90) | 1.30 (1.17-1.45) | 0.90 (0.43-1.90)$^{+}$ |
| Osteoporosis | 1 | 1.26 (1.14-1.39) | 1.25 (1.12-1.40) | 2.20 (1.55-3.12) |
| Atrial fibrillation | 1 | 1.40 (1.45-1.57) | 1.30 (1.17-1.45) | 1.32 (0.50-3.52)$^{+}$ |
| Anxiety | 1 | 1.22 (1.10-1.35) | 1.30 (1.16-1.44) | 1.48 (0.67-3.30)$^{+}$ |
| Inflammatory bowel disease | 1 | 1.37 (1.20-1.57) | 1.30 (1.17-1.44) | 1.30 (0.54-3.11)$^{+}$ |
| Heart failure | 1 | 2.69 (2.22-3.25) | 1.29 (1.16-1.43) | 5.14 (2.14-12.38) |

For peer review only

Table 5 Risk of MACE for individual index conditions in patients with RA and no index condition, RA with index condition, RA with no index condition and RA and index condition. Age-adjusted Cox's proportional hazards models were adjusted for sex, Townsend score, smoking status, alcohol intake frequency, BMI, physical activity level and rheumatoid factor status. Cox's proportional hazards p<0·01, except for those labelled with $^{+}$ indicating p>0.01. Index conditions labelled * have interaction term p<0.01

| | Risk of MACE | | | |
|---|---|---|---|---|
| | No RA, no index condition | No RA, with index condition HR, (95% CI) | RA, no index condition HR, (95% CI) | RA and index condition HR, (95% CI) |

| **Index condition** | | | | |
|---|---|---|---|---|
| Hypertension | 1 | 1.50 (1.44-1.55) | 1.48 (1.25-1.75) | 1.97 (1.66-2.33) |
| Coronary heart disease | 1 | 1.89 (1.80-1.98) | 1.43 (1.45-1.63) | 2.28 (1.76-2.98) |
| Diabetes | 1 | 1.67 (1.58-1.75) | 1.49 (1.31-1.69) | 1.69 (1.19-2.39) |
| Asthma | 1 | 1.12 (1.06-1.18) | 1.43 (1.25-1.63) | 1.47 (1.09-1.98) |
| Dyspepsia | 1 | 1.14 (1.08-1.20) | 1.39 (1.22- 1.58) | 1.85 (1.30-2.34) |
| Cancer | 1 | 1.11 (1.04-1.17) | 1.43 (1.26-1.62) | 1.44 (0.98-2.11)$^{+}$ |
| Depression | 1 | 1.25 (1.17-1.34) | 1.39 (1.22-1.58) | 2.06 (1.41-3.00) |
| Thyroid disorder | 1 | 1.14 (1.03-1.23) | 1.37 (1.20-1.55) | 2.10 (1.50-2.93) |
| COPD | 1 | 1.49 (1.37-1.62) | 1.40 (1.24-1.59) | 1.97 (1.33-2.92) |
| Epilepsy | 1 | 1.50 (1.30-1.73) | 1.41 (1.21-1.60) | 2.21 (0.83-5.88)$^{+}$ |
| Migraine | 1 | 0.99 (0.89-1.12)$^{+}$ | 1.40 (1.23-1.58) | 2.08 (1.12-3.87) |
| Psoriasis/Eczema | 1 | 1.05 (0.96-1.14)$^{+}$ | 1.42 (1.26-1.61) | 1.23 (0.64-2.37)$^{+}$ |
| Prostate disease | 1 | 0.92 (0.83-1.00)$^{+}$ | 1.41 (1.25-1.60) | 1.27 (0.64-2.54)$^{+}$ |
| Osteoporosis* | 1 | 1.34 (1.18-1.53) | 1.25 (1.10-1.41) | 3.17 (2.17-4.64) |
| Atrial fibrillation | 1 | 1.41 (1.25-1.60) | 1.72 (1.53-1.93) | 2.67 (1.99-5.95) |
| Anxiety | 1 | 1.28 (1.14-1.43) | 1.40 (1.24-1.59) | 2.73 (1.30-5.72) |
| Inflammatory bowel disease | 1 | 1.09 (0.92-1.29)$^{+}$ | 1.42 (1.26-1.60) | 1.11 (0.36-3.44)$^{+}$ |
| Heart failure | 1 | 2.64 (2.15-3.24) | 1.41 (1.25-1.59) | 3.45 (1.11-10.70)$^{+}$ |

663

For peer review only

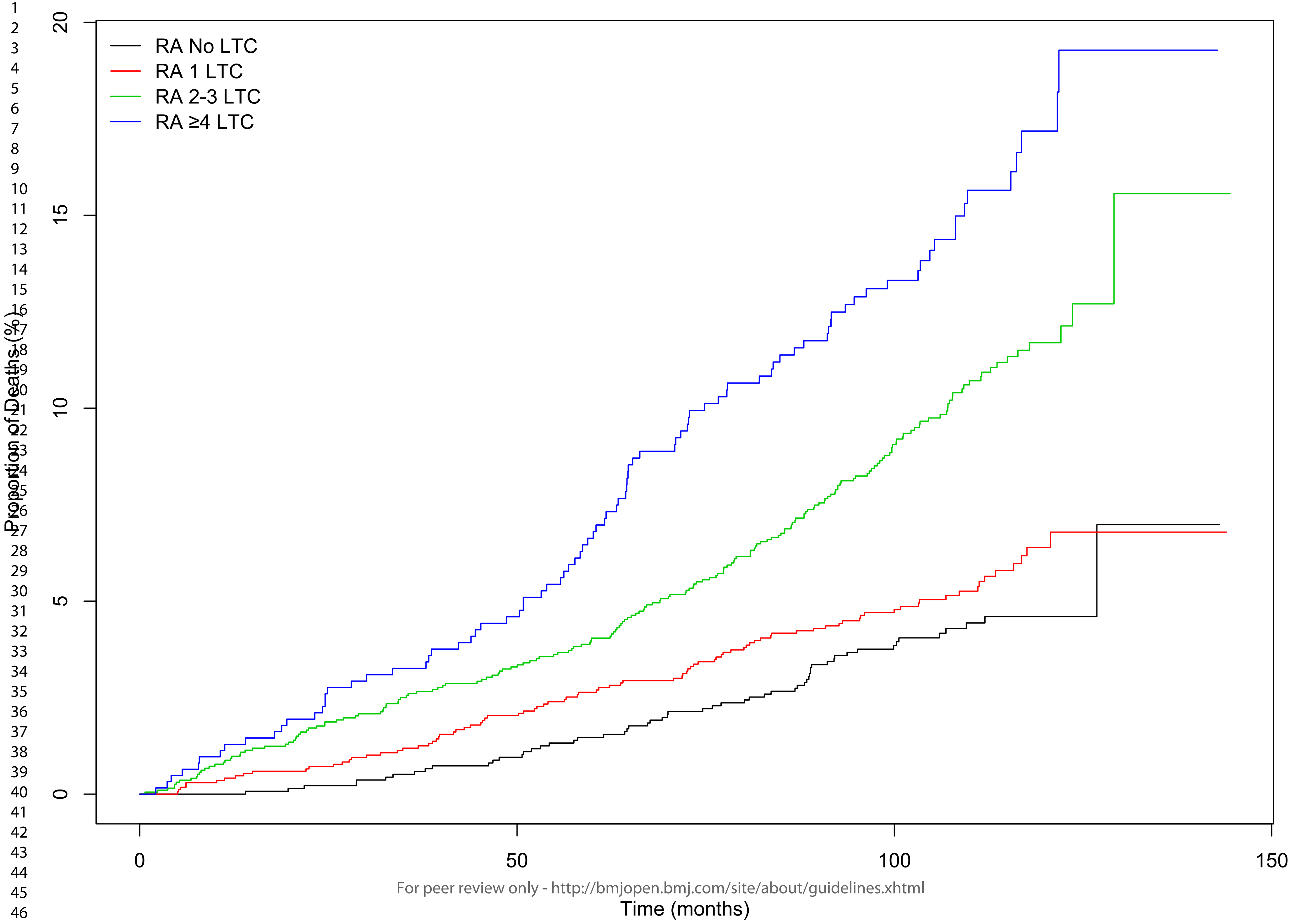


For peer review only - http://bmjopen.bmj.com/site/about/guidelines.xhtml

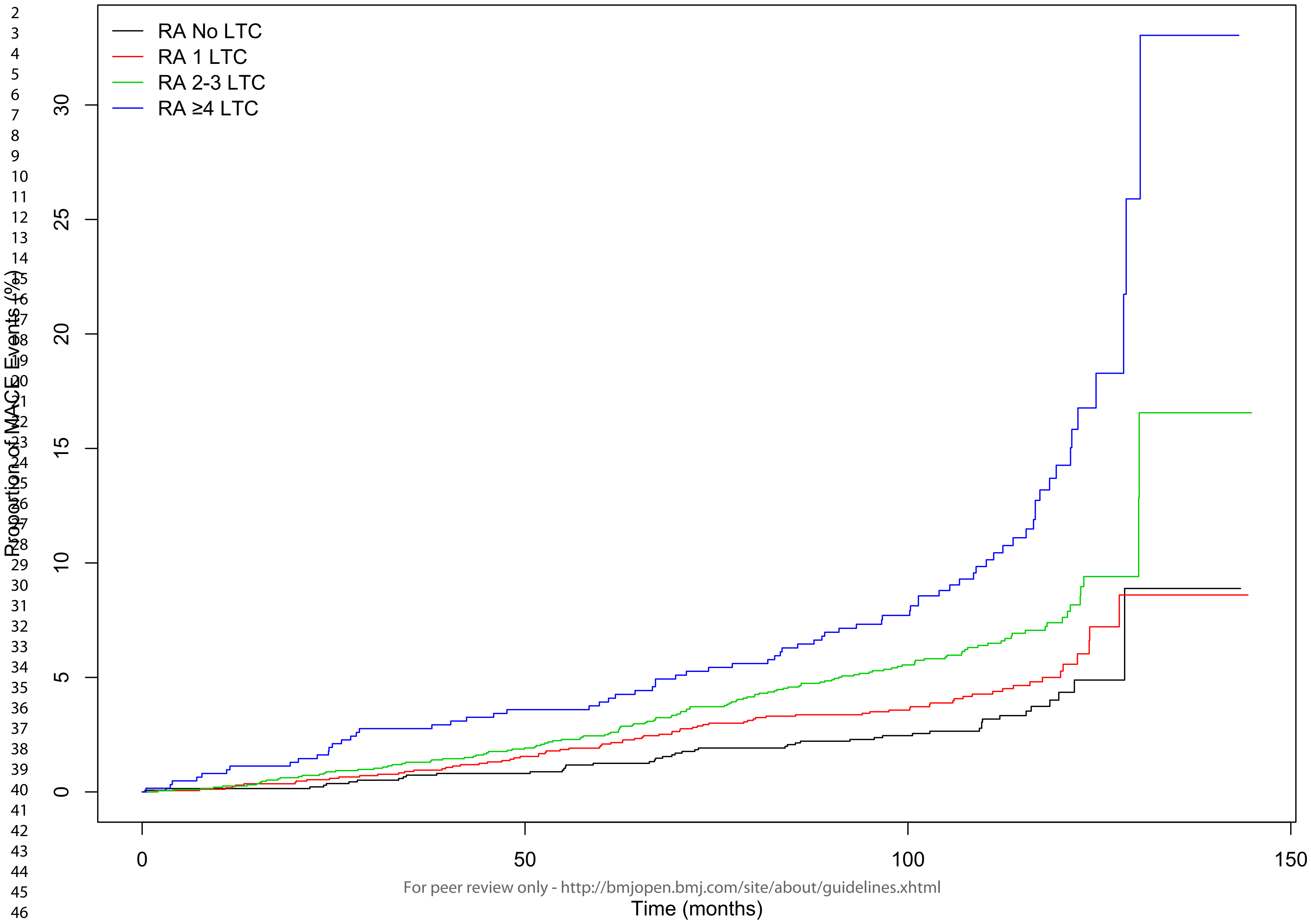
RA No LTC
RA 1 LTC
RA 2-3 LTC
RA ≥4 LTC
Proportion of MACE Events (%)
0
5
10
15
20
25
30
0
50
100
150
Time (months)

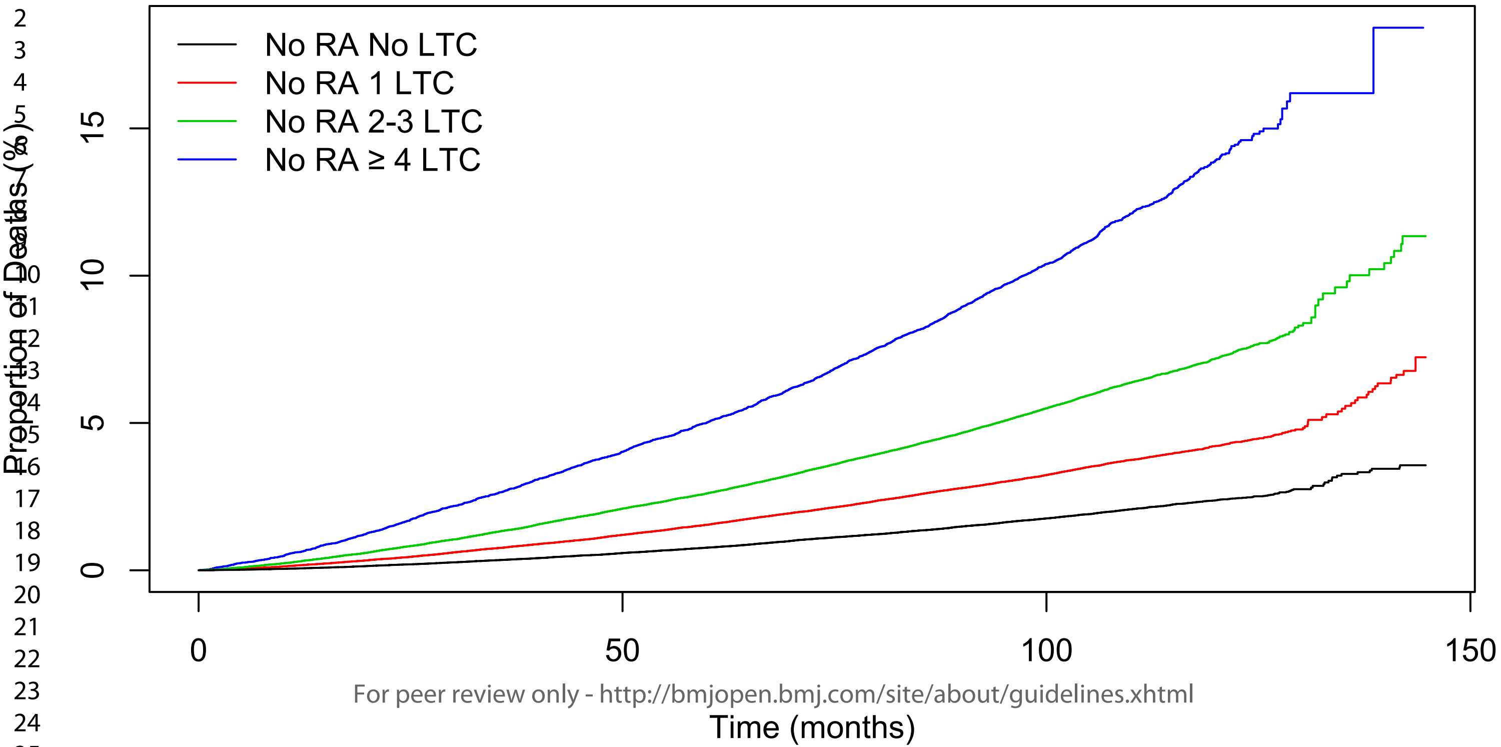


For peer review only - http://bmjopen.bmj.com/site/about/guidelines.xhtml

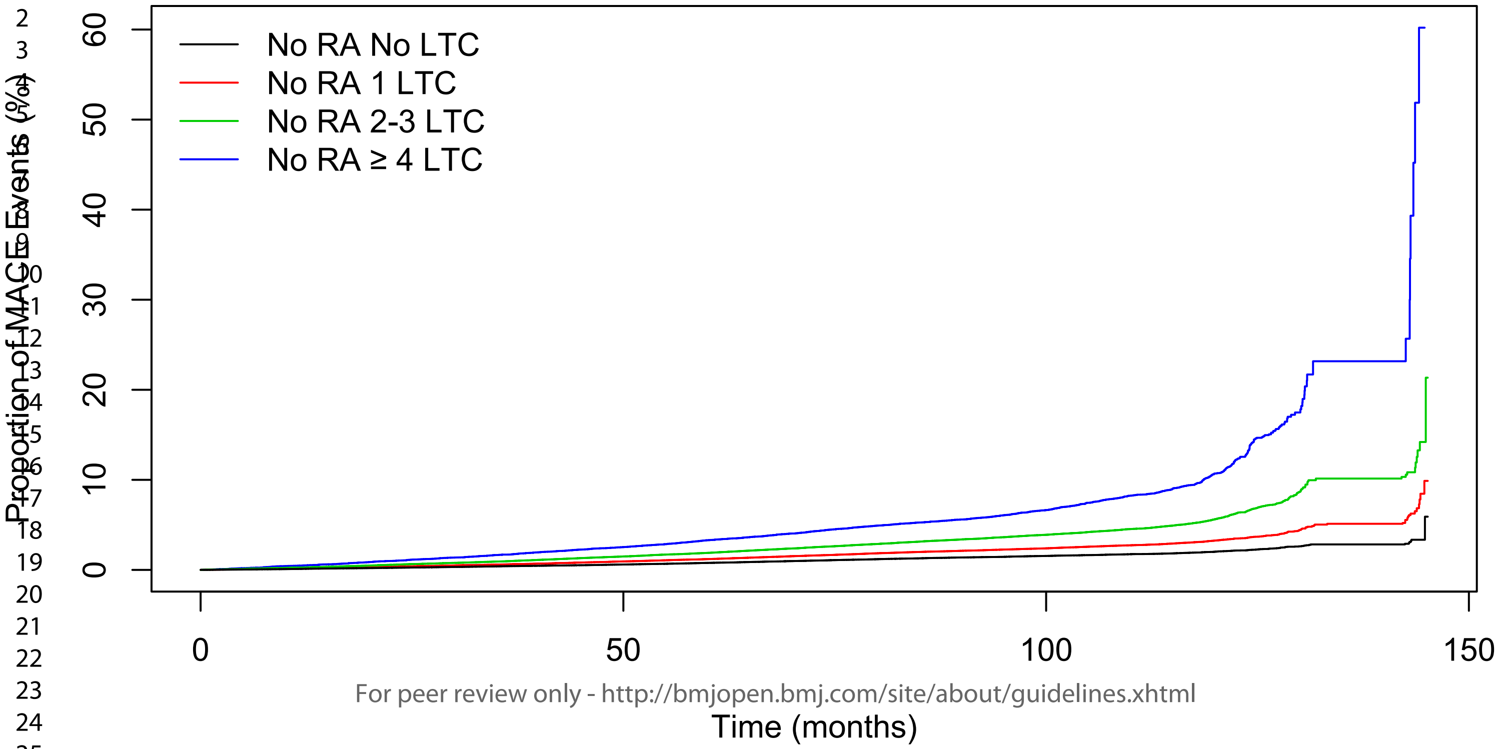


For peer review only - http://bmjopen.bmj.com/site/about/guidelines.xhtml

*Tables*

Supplementary table 1 – Proportion of long term conditions in participants with and without RA. P value determined using χ2 testing.

| **Condition** | **Prevalence in RA participants (%)** | **Prevalence in non-RA participants (%)** | **p value** |
|---|---|---|---|
| **Hypertension** | **35·6** | **26·4** | **<0·01** |
| **Asthma** | **15·4** | **11·6** | **<0·01** |
| **Dyspepsia** | **11·3** | **7·7** | **<0·01** |
| **Thyroid disorder** | **9·5** | **5·8** | **<0·01** |
| **Cancer** | **8·7** | **7·7** | **<0·01** |
| **Coronary heart disease** | **8·2** | **4·5** | **<0·01** |
| **Diabetes** | **7·6** | **5·0** | **<0·01** |
| **Depression** | **7·0** | **5·6** | **<0·01** |
| **Osteoporosis** | **4·9** | **1·5** | **<0·01** |
| **Chronic obstructive pulmonary disease** | **4·4** | **1·6** | **<0·01** |
| Psoriasis/eczema | 4·1 | 3·5 | 0·03 |
| **IBS** | **3·3** | **2·3** | **<0·01** |
| Migraine | 3·2 | 2·9 | 0·04 |
| **Stroke/TIA** | **3·1** | **1·7** | **<0·01** |
| **Diverticular disease** | **2·2** | **1·1** | **<0·01** |
| Anxiety | 1·7 | 1·8 | 0·47 |
| **IBD** | **1·4** | **0·8** | **<0·01** |
| Prostate disease | 1·3 | 1·6 | 0·06 |
| **Pernicious anaemia** | **1·2** | **0·3** | **<0·01** |
| Glaucoma | 1·2 | 1·1 | 0·26 |
| Epilepsy | 1·2 | 0·8 | 0·38 |
| Endometriosis | 0·9 | 0·8 | 0·39 |
| Atrial fibrillation | 0·9 | 0·7 | 0·14 |
| **Peripheral vascular disease** | **0·9** | **0·3** | **<0·01** |
| **Chronic bronchitis** | **0·8** | **0·3** | **<0·01** |
| Chronic sinusitis | 0·8 | 0·6 | 0·34 |
| **Meniere's disease** | **0·7** | **0·3** | **<0·01** |
| Chronic kidney disease | 0·5 | 0·3 | 0·01 |
| **Chronic liver disease** | **0·4** | **0·2** | **<0·01** |
| Schizophrenia | 0·4 | 0·4 | 0·68 |
| Chronic fatigue syndrome | 0·4 | 0·4 | 0·42 |
| Alcohol problems | 0·4 | 0·2 | 0·02 |
| Viral hepatitis | 0·3 | 0·3 | 0·91 |
| Heart failure | 0·3 | 0·2 | 0·18 |
| Polycystic ovary syndrome | 0·2 | 0·1 | 0·08 |
| Multiple sclerosis | 0·2 | 0·4 | 0·03 |
| Parkinson's disease | 0·1 | 0·2 | 0·71 |
| Constipation | 0·1 | 0·1 | 0·81 |
| Dementia | 0·1 | 0·02 | 0·17 |
| Anorexia/bulimia | 0·1 | 0·1 | 0·80 |

For peer review only

| | | | |
|---|---|---|---|
| Psychoactive substance misuse | 0·03 | 0·02 | 0·30 |

For peer review only

Supplementary table 2 –Medications, primary care read codes and hospitalisation codes used for RA self-report verification

| **Medications** | **Primary care read codes** | **Hospitalisation ICD-10 codes** |
|---|---|---|
| Depomedrone | 14G1 | M05 |
| Triamcinilone | F3712 | M06 |
| Methylprednisolone | F3964 | |
| Prednisolone | G5yA. | |
| Prednisone | G5y8. | |
| Auranofin | H570. | |
| Azathioprine | N04.. | |
| Hydroxychloroquine | N040. | |
| leflunomide | N0400 | |
| Methotrexate | N0401 | |
| Myocrisin | N0402 | |
| Penicillamine | N0403 | |
| Sulfasalazine | N0404 | |
| Abatacept | N0405 | |
| Adalimumab | N0406 | |
| Certolizumab | N0407 | |
| Etanercept | N0408 | |
| Golimumab | N0409 | |
| Infliximab | N040A | |
| Rituximab | N040B | |
| Tocilizumab | N040C | |
| | N040D | |
| | N040E | |
| | N040F | |
| | N040G | |
| | N040H | |
| | N040J | |
| | N040K | |
| | N040L | |
| | N040M | |
| | N040N | |
| | N040P | |
| | N040Q | |
| | N040R | |
| | N040S | |
| | N040T | |
| | N041. | |
| | N042. | |
| | N0421 | |
| | N0422 | |
| | N042z | |
| | N043. | |
| | N0430 | |

For peer review only

N0431
N0432
N0433
N043z
N047.
N04X.
N04y2
N0455
Nyu10
Nyu11
Nyu12
Nyu1G

For peer review only

Supplementary table 3 – Proportion of rheumatoid arthritis related hospitalisation, medication or primary care read code in participants who self-report rheumatoid arthritis.

| *Rheumatoid arthritis self-report* | *Any rheumatoid arthritis hospitalisation, medication or primary care read code* | | ***Total*** |
|---|---|---|---|
| | No | Yes | |
| No | 141152<br>74.4 % | 48634<br>25.6 % | 189786<br>100 % |
| Yes | 513<br>12.2 % | 3683<br>87.8 % | 4196<br>100 % |
| ***Total*** | 141665<br>73 % | 52317<br>27 % | 193982<br>100 % |

For peer review only

Supplementary table 4 – Demographic factors, lifestyle factors, number of long-term conditions and rheumatoid factor status in patients with and without RA. Unless indicated, p<0·01. Chi squared test used for categorical variables, Kruskal-Wallis test used for continuous variables. SD = standard deviation. RA defined here as RA self-report plus hospitalisation, medication or primary care read code related to rheumatoid arthritis.

| | **Participants with RA (%) (N=3683)** | **Participants without RA (%) (N=498857)** |
|---|---|---|
| **Mean Age (years (SD)); missing values =0 (0%)** | 59.2 (7.1) | 56.5 (8.1) |
| **Age (years); missing values = 0 (0%)** | | |
| 37-49 | 413<br>11.2 % | 117470<br>23.5 % |
| 50-59 | 1161<br>31.5 % | 165992<br>33.3 % |
| 60-73 | 2109<br>57.3 % | 215388<br>43.2 % |
| **Sex; missing values = 0 (0%)** | | |
| Female | 2672<br>72.5 % | 270729<br>54.3 % |
| Male | 1011<br>27.5 % | 228121<br>45.7 % |
| **Townsend score; missing values = 623 (0·12%)** | | |
| 0-20 | 672<br>18.3 % | 99991<br>20.1 % |
| 20-40 | 666<br>18.1 % | 99430<br>20 % |
| 40-60 | 735<br>20 % | 99663<br>20 % |
| 60-80 | 760<br>20.7 % | 99615<br>20 % |
| 80-100 | 847<br>23 % | 99531<br>20 % |
| **Smoking status; missing values = 2950 (0·59%)** | | |
| Never | 1679<br>46 % | 271857<br>54.8 % |
| Current or Previous | 1973<br>54 % | 224074<br>45.2 % |
| **Frequency of alcohol intake; missing values = 1502 (0·30%)** | | |
| Never or special occasions only | 1218<br>33.1 % | 97442<br>19.6 % |
| One to three times a month | 453<br>12.3 % | 55405<br>11.1 % |
| One to four times a week | 1504<br>40.9 % | 243237<br>48.9 % |
| Daily or almost daily | 504<br>13.7 % | 101268<br>20.4 % |
| **BMI (kg/m$^2$); missing values = 5820 (1·15%)** | | |
| underweight <18.5 | 34<br>0.9 % | 2592<br>0.5 % |

For peer review only

| | | |
|---|---|---|
| normal weight 18.5-24.9 | 1084<br>30 % | 156353<br>31.7 % |
| overweight 25-29.9 | 1425<br>39.5 % | 212799<br>43.2 % |
| obese >=30s | 1067<br>29.6 % | 121359<br>24.6 % |
| **Physical activity; missing values = 7156 (1·42%)** | | |
| none | 595<br>16.6 % | 32254<br>6.6 % |
| low | 286<br>8 % | 18652<br>3.8 % |
| medium | 2596<br>72.4 % | 390922<br>79.5 % |
| high | 107<br>3 % | 49965<br>10.2 % |
| **Number of long-term conditions; missing values = 1845 (0·36%)** | | |
| 0 | 922<br>25.2 % | 174293<br>35.1 % |
| 1 | 1103<br>30.1 % | 163244<br>32.8 % |
| 2-3 | 1255<br>34.3 % | 135091<br>27.2 % |
| ≥4 | 379<br>10.4 % | 24401<br>4.9 % |
| **Rheumatoid Factor (IU/ml); missing values = 33,066 (6·6%)** | | |
| <20 | 1801<br>52.4 % | 449067<br>96.4 % |
| ≥20 | 1639<br>47.6 % | 16960<br>3.6 % |

For peer review only

Supplementary Table 5 – Relationship between long term conditions and all-cause mortality in participants with and without RA using age-adjusted multivariate Cox's proportional hazards regression analysis. Unless otherwise shown, Cox's proportional hazards p<0·01. RA defined here as RA self-report plus hospitalisation, medication or primary care read code related to rheumatoid arthritis.

| Risk of all-cause mortality | | | |
|---|---|---|---|
| **Comorbidity status (reference: *No RA and no other long-term conditions*)** | | **Adjusted for sex, Townsend score, alcohol status, smoking status, BMI, physical activity and rheumatoid factor status HR (95% CI)** | **Number of deaths (%)** |
| **No other long-term conditions** | **RA** | 1.50 (1.09 – 2.07) | 44 (4.8%) |
| **1 other long-term condition** | **No RA** | 1.39 (1.33 - 1.46) | 5810 (3.6%) |
| | **RA** | 1.42 (1.07 - 1.88) | 66 (5.9%) |
| **2-3 other long-term conditions** | **No RA** | 1.83 (1.75 - 1.91) | 7966 (5.9%) |
| | **RA** | 2.75 (2.29 - 3.30) | 142 (11.3%) |
| **≥4 other long-term conditions** | **No RA** | 2.70 (2.55 - 2.86) | 2461 (10.8%) |
| | **RA** | 2.98 (2.19 - 4.04) | 54 (14.2%) |

For peer review only

Supplementary Table 6 – Relationship between long term conditions and major adverse cardiovascular events in participants with and without RA using age-adjusted multivariate Cox's proportional hazards regression analysis. Unless otherwise shown, Cox's proportional hazards p<0·01. RA defined here as RA self-report plus hospitalisation, medication or primary care read code related to rheumatoid arthritis.

| Risk of MACE | | | |
|---|---|---|---|
| **Comorbidity status (reference: *No RA and no other long-term conditions*)** | | **Adjusted for sex, Townsend score, alcohol status, smoking status, BMI, physical activity and rheumatoid factor status HR (95% CI)** | **Number of MACE (%)** |
| **No other long-term conditions** | **RA** | 1.63 (1.13 - 2.36) | 32 (3.5%) |
| **1 other long-term condition** | **No RA** | 1.24 (1.18 - 1.30) | 4530 (2.8%) |
| | **RA** | 1.95 (1.46 - 2.59) | 60 (5.4%) |
| **2-3 other long-term conditions** | **No RA** | 1.66 (1.58 - 1.74) | 6244 (4.6%) |
| | **RA** | 2.50 (2.00 - 3.12) | 95 (7.6%) |
| **≥4 other long-term conditions** | **No RA** | 2.38 (2.23 - 2.54) | 2007 (8.2%) |
| | **RA** | 3.30 (2.36 - 4.61) | 46 (12.1%) |

For peer review only

Supplementary Table 7 – Table 4 Risk of all-cause mortality for individual index conditions in patients with RA and no index condition, RA with index condition, RA with no index condition or RA and index condition. Age-adjusted Cox's proportional hazards models were adjusted for sex, Townsend score, smoking status, alcohol intake frequency, BMI, physical activity level and level of rheumatoid factor. Unless otherwise shown, Cox's proportional hazards p<0·01. Index conditions labelled * have interaction term p>0.01. RA defined here as RA self-report plus hospitalisation, medication or primary care read code related to rheumatoid arthritis.

| | **Risk of all-cause mortality** | | | |
|---|---|---|---|---|
| **Index condition** | **No RA, no index condition HR, (95% CI), p** | **No RA, with index condition HR, (95% CI), p** | **RA, no index condition HR, (95% CI), p** | **RA and index condition HR, (95% CI), p** |
| Hypertension | 1 | 1.24 1.20-1.28 | 1.27 1.07-1.52 | 1.69 1.41-2.02 |
| Coronary heart disease | 1 | 1.58 1.50-1.65 | 1.30 1.13-1.50 | 2.08 1.55-2.79 |
| Diabetes | 1 | 1.68 1.60-1.76 | 1.37 1.20-1.57 | 1.76 1.22-2.54 |
| Asthma | 1 | 1.10 1.05-1.15 | 1.32 1.14-1.52 | 1.48 1.10-2.00 |
| Dyspepsia | 1 | 1.02 0.97-1.07 p=0.42 | 1.31 1.15-1.50 | 1.46 1.04-2.06 |
| Cancer | 1 | 2.50 2.41-2.60 | 1.43 1.25-1.65 | 2.72 1.99-3.70 |
| Depression | 1 | 1.28 1.20-1.35 | 1.32 1.16-1.51 | 1.79 1.17-2.75 |
| Thyroid disorder | 1 | 1.05 0.99-1.12 p=0.12 | 1.36 1.19-1.55 | 1.14 0.76-1.72 p=0.53 |
| COPD | 1 | 2.12 1.98-2.26 | 1.32 1.15-1.50 | 2.53 1.77-3.63 |
| Epilepsy | 1 | 1.62 1.43-1.84 | 1.33 1.17-1.51 | 2.15 0.80-5.72 p=0.13 |
| Migraine | 1 | 0.85 0.76-0.94 | 1.33 1.17-1.51 | 1.02 0.38-2.71 p=0.97 |
| Psoriasis /Eczema | 1 | 1.06 0.94-1.14 p=0.15 | 1.30 1.14-1.49 | 2.08 1.23-3.50 |
| Prostate disease | 1 | 0.83 0.75-0.90 | 1.32 1.16-1.51 | 1.33 0.55-3.19 p=0.52 |
| Osteoporosis | 1 | 1.27 1.16-1.40 | 1.29 1.13-1.48 | 2.09 1.38-3.14 |
| Atrial fibrillation | 1 | 1.40 1.25-1.58 | 1.34 1.18-1.52 | 0.99 0.25-3.98 p=0.99 |
| Anxiety | 1 | 1.23 1.11-1.36 | 1.34 1.18-1.53 | 0.72 0.18-2.89 p=0.64 |
| Inflammatory bowel disease | 1 | 1.38 1.21-1.58 | 1.35 1.18-1.53 | 0.63 0.16-2.51 p=0.51 |
| Heart failure | 1 | 2.71 2.25-3.28 | 1.32 1.16-1.51 | 4.34 1.39-13.43 |

For peer review only

Supplementary Table 8 – Risk of MACE for individual index conditions in patients with RA and no index condition, RA with index condition, RA with no index condition or RA and index condition. Age-adjusted Cox's proportional hazards models were adjusted for sex, Townsend score, smoking status, alcohol intake frequency, BMI, physical activity level and level of rheumatoid factor. Unless otherwise shown, p<0·01. Index conditions labelled * have interaction term p>0.01. RA defined here as RA self-report plus hospitalisation, medication or primary care read code related to rheumatoid arthritis.

| | **Risk of MACE** | | | |
|---|---|---|---|---|
| **Index condition** | **No RA, no index condition HR, (95% CI), p** | **No RA, with index condition HR, (95% CI), p** | **RA, no index condition HR, (95% CI), p** | **RA and index condition HR, (95% CI), p** |
| Hypertension | 1 | 1.49 1.44-1.55 | 1.55 1.26-1.90 | 2.26 1.85-2.76 |
| Coronary heart disease | 1 | 1.89 1.80-1.98 | 1.60 1.37-1.88 | 2.31 1.65-3.22 |
| Diabetes | 1 | 1.66 1.58-1.75 | 1.62 1.39-1.90 | 1.66 1.58-1.75 |
| Asthma | 1 | 1.12 1.06-1.17 | 1.57 1.34-1.84 | 1.67 1.19-2.36 |
| Dyspepsia | 1 | 1.14 1.08-1.20 | 1.55 1.33-1.82 | 1.80 1.23-2.64 |
| Cancer | 1 | 1.11 1.05-1.17 | 1.59 1.37-1.85 | 1.42 0.87-2.33 p=0.16 |
| Depression | 1 | 1.25 1.17-1.34 | 1.53 1.31-1.78 | 2.38 1.52-3.74 |
| Thyroid disorder | 1 | 1.14 1.06-1.23 | 1.50 1.28-1.75 | 2.32 1.59-3.36 |
| COPD | 1 | 1.50 1.38-1.63 | 1.58 1.36-1.84 | 1.81 1.09-3.00 |
| Epilepsy | 1 | 1.50 1.31-1.74 | 1.56 1.35-1.81 | 1.74 0.44-6.97 p=0.43 |
| Migraine | 1 | 1.00 0.90-1.12 p=0.96 | 1.54 1.33-1.79 | 2.41 1.08-5.37 |
| Psoriasis /Eczema | 1 | 1.05 0.96-1.14 p=0.29 | 1.56 1.34-1.80 | 1.72 0.86-3.44 p=0.12 |
| Prostate disease | 1 | 0.91 0.83-1.00 p=0.05 | 1.53 1.32-1.78 | 2.53 1.20-5.31 p=0.01 |
| Osteoporosis* | 1 | 1.27 1.12-1.43 | 1.48 1.28-1.73 | 3.15 2.03-4.90 |
| Atrial fibrillation | 1 | 1.72 1.53-1.93 | 1.56 1.35-1.81 | 2.78 1.04-7.43 p=0.04 |
| Anxiety | 1 | 1.29 1.15-1.44 | 1.56 1.35-1.81 | 2.29 0.86-6.10 p=0.09 |
| Inflammatory bowel disease | 1 | 1.09 0.92-1.29 p=0.30 | 1.57 1.36-1.82 | 0.90 0.23-3.63 p=0.89 |
| Heart failure | 1 | 2.67 2.18-3.28 | 1.57 1.35-1.81 | 1.71 1.35-12.17 p=0.59 |

For peer review only

# Reporting checklist for cross sectional study.

Based on the STROBE cross sectional guidelines.

## Instructions to authors

Complete this checklist by entering the page numbers from your manuscript where readers will find each of the items listed below.

Your article may not currently address all the items on the checklist. Please modify your text to include the missing information. If you are certain that an item does not apply, please write "n/a" and provide a short explanation.

Upload your completed checklist as an extra file when you submit to a journal.

In your methods section, say that you used the STROBE cross sectionalreporting guidelines, and cite them as:

von Elm E, Altman DG, Egger M, Pocock SJ, Gotzsche PC, Vandenbroucke JP. The Strengthening the Reporting of Observational Studies in Epidemiology (STROBE) Statement: guidelines for reporting observational studies.

For peer review only

| | | Reporting Item | Page Number |
|---|---|---|---|
| **Title and abstract** | | | |
| Title | #1a | Indicate the study’s design with a commonly used term in the title or the abstract | 1 |
| Abstract | #1b | Provide in the abstract an informative and balanced summary of what was done and what was found | 2 |

### Introduction

| | | | |
|---|---|---|---|
| Background / rationale | #2 | Explain the scientific background and rationale for the investigation being reported | 5 |
| Objectives | #3 | State specific objectives, including any prespecified hypotheses | 5 |

### Methods

| | | | |
|---|---|---|---|
| Study design | #4 | Present key elements of study design early in the paper | 6 |
| Setting | #5 | Describe the setting, locations, and relevant dates, including periods of recruitment, exposure, follow-up, and data collection | 6 |
| Eligibility criteria | #6a | Give the eligibility criteria, and the sources and methods of selection of participants. | n/a (data collected by UK Biobank) |
| | #7 | Clearly define all outcomes, exposures, predictors, potential confounders, and effect modifiers. Give diagnostic criteria, if applicable | 6 |
| Data sources / measurement | #8 | For each variable of interest give sources of data and details of methods of assessment (measurement). Describe comparability of assessment methods if there is more than one group. Give information separately for for exposed and unexposed groups if applicable. | 6-7 |

For peer review only

| | | | |
|---|---|---|---|
| Bias | #9 | Describe any efforts to address potential sources of bias | n/a (data collected by UK Biobank) |
| Study size | #10 | Explain how the study size was arrived at | 6 |
| Quantitative variables | #11 | Explain how quantitative variables were handled in the analyses. If applicable, describe which groupings were chosen, and why | 6-7 |
| Statistical methods | #12a | Describe all statistical methods, including those used to control for confounding | 8 |
| Statistical methods | #12b | Describe any methods used to examine subgroups and interactions | 8 |
| Statistical methods | #12c | Explain how missing data were addressed | 8 |
| Statistical methods | #12d | If applicable, describe analytical methods taking account of sampling strategy | 8 |
| Statistical methods | #12e | Describe any sensitivity analyses | 8 |
| **Results** | | | |
| Participants | #13a | Report numbers of individuals at each stage of study—eg numbers potentially eligible, examined for eligibility, confirmed eligible, included in the study, completing | 9 |

For peer review only

| | | | |
|---|---|---|---|
| | | follow-up, and analysed. Give information separately for for exposed and unexposed groups if applicable. | |
| Participants | #13b | Give reasons for non-participation at each stage | 9 |
| Participants | #13c | Consider use of a flow diagram | n/a (not applicable here) |
| Descriptive data | #14a | Give characteristics of study participants (eg demographic, clinical, social) and information on exposures and potential confounders. Give information separately for exposed and unexposed groups if applicable. | 9 |
| Descriptive data | #14b | Indicate number of participants with missing data for each variable of interest | 9 |
| Outcome data | #15 | Report numbers of outcome events or summary measures. Give information separately for exposed and unexposed groups if applicable. | 10-13 |
| Main results | #16a | Give unadjusted estimates and, if applicable, confounder-adjusted estimates and their precision (eg, 95% confidence interval). Make clear which confounders were adjusted for and why they were included | 10-13 |
| Main results | #16b | Report category boundaries when continuous variables were categorized | 10-13 |

For peer review only

| | | | |
|---|---|---|---|
| Main results | #16c | If relevant, consider translating estimates of relative risk into absolute risk for a meaningful time period | 10-13 |
| Other analyses | #17 | Report other analyses done—e.g., analyses of subgroups and interactions, and sensitivity analyses | 13 |
| **Discussion** | | | |
| Key results | #18 | Summarise key results with reference to study objectives | 13 |
| Limitations | #19 | Discuss limitations of the study, taking into account sources of potential bias or imprecision. Discuss both direction and magnitude of any potential bias. | 15 |
| Interpretation | #20 | Give a cautious overall interpretation considering objectives, limitations, multiplicity of analyses, results from similar studies, and other relevant evidence. | 14 |
| Generalisability | #21 | Discuss the generalisability (external validity) of the study results | 16-17 |
| **Other Information** | | | |
| Funding | #22 | Give the source of funding and the role of the funders for the present study and, if applicable, for the original study on which the present article is based | 17 |

Notes:

- 6a: n/a (data collected by UK Biobank)
- 9: n/a (data collected by UK Biobank)

For peer review only

- 13c: n/a (not applicable here) The STROBE checklist is distributed under the terms of the Creative Commons Attribution License CC-BY. This checklist was completed on 25. March 2020 using https://www.goodreports.org/, a tool made by the EQUATOR Network in collaboration with Penelope.ai

For peer review only
